# Structural bioinformatic studies of eight integral transmembrane NADPH oxidases and their AlphaFold 3 predicted QTY analogs with reduced hydrophobicity

**DOI:** 10.1371/journal.pone.0347525

**Published:** 2026-06-10

**Authors:** Tutu Hu, Rick Cheng, Edward Chen, Shuguang Zhang

**Affiliations:** 1 Tabor Academy, Marion, Massachusetts, United States of America; 2 The Lawrenceville School, Lawrenceville, New Jersey, United States of America; 3 Media Lab, Massachusetts Institute of Technology, Cambridge, Massachusetts, United States of America; Chung-Ang University, KOREA, REPUBLIC OF

## Abstract

The NADPH oxidase (NOX) family comprises integral membrane-bound enzymes responsible for generating reactive oxygen species (ROS), with critical roles in immune defense, vascular regulation, and cellular signaling. However, their intrinsic hydrophobicity and membrane association created longstanding challenges for extensive research. In this study, we applied the QTY code – a simple protein design strategy that replaces hydrophobic residues leucine (L), isoleucine (I), valine (V), and phenylalanine (F) with hydrophilic yet structurally compatible residues glutamine (Q), threonine (T) and tyrosine (Y) – to generate QTY analogs of NOX1, NOX2, NOX3, NOX4, NOX5, DUOX1, DUOXA1 and CYBA with reduced hydrophobicity. Using AlphaFold 3, we predicted and superposed the structures of native and QTY-engineered analog proteins. Our results show strong structural resemblance between each pair, with root mean square deviation (RMSD) values below 1Å for six out of eight proteins examined. In addition, QTY substitution significantly reduced surface hydrophobicity, indicating improved water-solubility while preserving 3D structural fold integrity. Our findings demonstrate the potential of QTY-designed NOX variants with reduced hydrophobicity as surrogates for use in structural biology, monoclonal antibody discoveries, drug discovery, and other applications where native membrane proteins present experimental limitations.

## Introduction

Transmembrane proteins are abundant (~26–30%) and are essential for mediating metabolism, cell signaling, transport, and other vital cellular functions [1]. Despite their importance, they remain poorly studied due to experimental challenges. For instance, while transmembrane proteins are essential in mediating the metastatic process, they are inadequately characterized [2].

Traditional protein structure determination relies on X-ray crystallography and NMR spectroscopy. Recently, cryo-electron microscopy (CryoEM) has emerged as the mainstream technique for studying protein structures at higher resolutions [3]. In this study, we incorporated CryoEM-determined structures for those proteins for which such data were available, with resolutions ranging from 2.30Å to 3.20Å; structural information for the remaining proteins was not yet available at the time of writing. Crystallization of integral proteins is particularly challenging due to their inherent hydrophobicity [4]. Detergents are often required to solubilize these proteins, but their use is expensive, especially in the large quantities required for structural studies [5]. Therefore, we present a structural bioinformatics approach to study transmembrane proteins.

We focus on six integral membrane proteins from the NOX (NADPH oxidase 1–5) family/ DUOX (dual oxidase) family, one protein from the DUOXA (dual oxidase maturation factor) family, and one protein from the p22^phox^ (cytochrome b-245 light chain) family. These protein families are involved in electron transport across plasma membranes and serve as a source of reactive oxygen species (ROS) across diverse cell and tissue types [6], playing a crucial role in biological processes. Specifically, the DUOXA family facilitates DUOX maturation, while p22^phox^ functions as a scaffold protein that assembles subunits necessary for NOX-mediated ROS generation [7,8].

The NOX/DUOX family comprises complex, multi-domain proteins that require the assembly of other proteins to function. Characterized for their role in producing ROS for microbial killing in innate immunity, NOX/DUOX proteins participate in diverse physiological functions including host defense, post-translational protein processing, and cell signaling [7,9]. The NOX/DUOX family includes seven catalytic homologs: NOX1, NOX2, NOX3, NOX4, NOX5, and the dual oxidases DUOX1 and DUOX2. Each homolog acts as a catalytic subunit of the NADPH oxidase complex responsible for ROS production.

The structure of a prototypical NOX enzyme, particularly NOX2 found in phagocytes, includes both membrane-bound and cytosolic components. The complete, active enzyme is assembled from several key components: three cytosolic proteins (p40^phox^, p47^phox^ and p67^phox^), a membrane bound complex known as cytochrome b558 (composed of the catalytic NOX2 subunit gp91phox and the auxiliary p22^phox^ subunit), and a small GTP-binding protein Rac1, which acts as a molecular switch to initiate activation [10]. Upon activation, the cytosolic components translocate to the membrane and interact with cytochrome b558, forming the active enzyme complex capable of producing superoxide by transferring electrons from NADPH to molecular oxygen [10].

All NADPH oxidases have a homologous catalytic subunit responsible for transferring electrons across the cell membrane and subsequent ROS production [10]. However, the requirement of subunits varies among family members: NOX1–3 require p22^phox^ and the recruitment of cytosolic subunits p47, p67, p40, and Rac1, whereas NOX4 only requires p22^phox^ [10].

While generally similar in structure, these different isoforms are found in different cell types and tissues throughout the human body, serving a diverse range of functions.

NOX1 is primarily expressed in the colon but can also be found in the stomach, uterus, prostate, and vascular smooth muscle cells [11,12]. While the exact physiological function of NOX1 remains unclear, it is hypothesized to play a role in host defense through bacterial killing by ROS production, as well as in signaling pathways where ROS functions as a signaling molecule [11,12]. Defects in NOX1 have been linked to very early onset inflammatory bowel disease (VEOIBD), which encompasses Crohn’s disease and ulcerative colitis [13].

NOX2, the most well-studied NADPH oxidase, is expressed in phagocytic cells such as neutrophils and macrophages and plays a vital role in the human immune system. Like most other NADPH oxidases, NOX2 remains largely dormant under normal circumstances, becoming only activated during respiratory burst–a rapid release of ROS aimed at destroying microbial pathogens. Mutations in NOX2 have been linked to chronic granulomatous disease (CGD) [10].

NOX3 is expressed almost exclusively in the inner ear, where it functions in both the auditory and vestibular systems and is linked to the biosynthesis of otoconia [14].

NOX4 is primarily expressed in the kidney, although it is widely expressed and can also be found in many cell types throughout the body, including the heart, pancreas, osteoclasts, and vasculature [10]. It is thought to be constitutively active, with studies reporting that it produces hydrogen peroxide rather than superoxide as its main ROS product [15,16]. Unlike other isoforms, NOX4 does not require cytosolic activators beyond p22 [10].

The NOX5 protein, a member of the NOX/DUOX family, is expressed in thyroid, spermatocytes of testis, B- and T-lymphocyte-rich areas of spleen, and lymph nodes [16]. NOX5 contains four EF hand motifs with four Ca^2+^ binding sites at its N-terminus, regulated by intracellular Ca^2+^ concentration [17,18]. NOX5 has a NADPH binding site at the C-terminus [19]. Increase in NOX5 is associated with different oxidative stress-related pathologies, including cancer and cardiovascular diseases [20]. However, because rodents lack the genes encoding NOX5 and human NOX proteins have yet to be crystallized, NOX5’s functionality remains poorly studied [19,20]. Upon Ca^2+^ activation, NOX5 functions as a proton channel [21].

DUOX1, another member of the NOX/DUOX family, is a peroxidase that catalyzes the hydrogen peroxide production [22]. DUOX1 is found in tissues including the thyroid and respiratory tract, and plays a crucial role in thyroid hormone synthesis [22]. DUOX1 expression is often associated with disease pathophysiology [23]. DUOX1 also contains an N-terminal extracellular peroxidase homologous domain with two Ca^++^ EF-hand binding sites [24]. DUOX1 produces H_2_O_2_ extracellularly, which is essential for thyroid hormone synthesis [24].

DUOXA1, a member of the DUOXA family, is a maturation factor for DUOX1, together forming an active enzyme complex [22]. Structurally, the DUOXA1 protein comprises five helical transmembrane regions [23]. DUOXA1 facilitates DUOX1 maturation by binding to immature DUOX1 in the endoplasmic reticulum to assist in proper folding and glycosylation [23]. Together with DUOX1, this enzyme complex produces H_2_O_2_, a key component in thyroid hormone synthesis [24].

The p22^phox^ protein family, when coupled with NOX2, forms the phagocyte NADPH oxidase protein complex [25]. This complex produces superoxide anions, which are essential for innate immunity [25]. p22^phox^, a phosphorylated protein, directly promotes NADPH oxidase activity [26]. Moreover, NOX3 biosynthesis requires p22^phox^, which promotes NOX3 glycosylation and maturation [27].

CYBA (Cytochrome b-245 light chain) protein, a member of the p22^phox^ family, is a subcomponent of the NOX2 enzyme [28,29]. CYBA is expressed in phagocytes, where it is responsible for producing superoxide in response to bacterial and fungal infections [30].

Given their diverse biological roles and implications in a wide range of diseases, the NADPH oxidase enzymes are of considerable biomedical interest. While there have been many advancements in protein structure technology, such as CryoEM, detailed structural studies of NADPH remain limited due to the inherent properties of membrane proteins, notably their partially hydrophobic surfaces. Consequently, detergents are needed for protein solubilization during experimentation, which are expensive [4].

To address this challenge, our current study applies the QTY code design, a simple protein engineering method that replaces hydrophobic residues without dramatically altering the protein structure. L, I, V, and F are replaced with the hydrophilic but structurally similar amino acids: Q, T, and Y, respectively [31,32]. A previous study has shown that applying the QTY code to several chemokine receptors does not significantly impact their thermostabilities, α-helical structures, or ligand-binding activities [31].

AlphaFold 3 is the latest generation of deep learning models developed by Google DeepMind for predicting protein structures, interactions, and complexes with unprecedented accuracy. Unlike its predecessors, AlphaFold 3 extends beyond predicting individual protein structures to model ligands, ions, nucleic acids, and modified residues [33]. This enhanced capability enables high-confidence structural predictions of both native and QTY-variant forms of the NOX enzymes in our study–proteins that are challenging to study experimentally due to their membrane-embedded nature. However, the AlphaFold 3 model has limitations, underscoring the need for experimental validation of computational results.

The application of AlphaFold 3 to QTY code has expanded across various protein families. Previous studies used AlphaFold 2 to predict QTY protein analog folding in native chemokine receptors [34], glucose transporters [35], human solute carrier transporters [36], ABC transporters [32], serotonin, dopamine, and norepinephrine transporters [37], and glutamate transporters [38]. Additionally, reverse QTY code has been applied to human albumin to improve its alpha helices’ hydrophobicity [39]. The CXCR4^QTY^ construct has found practical application in biometric sensor building [40].

The advancement of AlphaFold 3 has significantly improved prediction accuracy while expanding its capability to predict proteins’ interactions with ligands, RNA, and other molecules [41]. Recent application of the QTY code with AlphaFold 3 predictions has been successfully used in predicting the binding mechanisms of FACE1 and STEA4 [42].

## Results & Discussion

### NADPH oxidase protein sequence alignments and other characteristics

**Fig 1:**
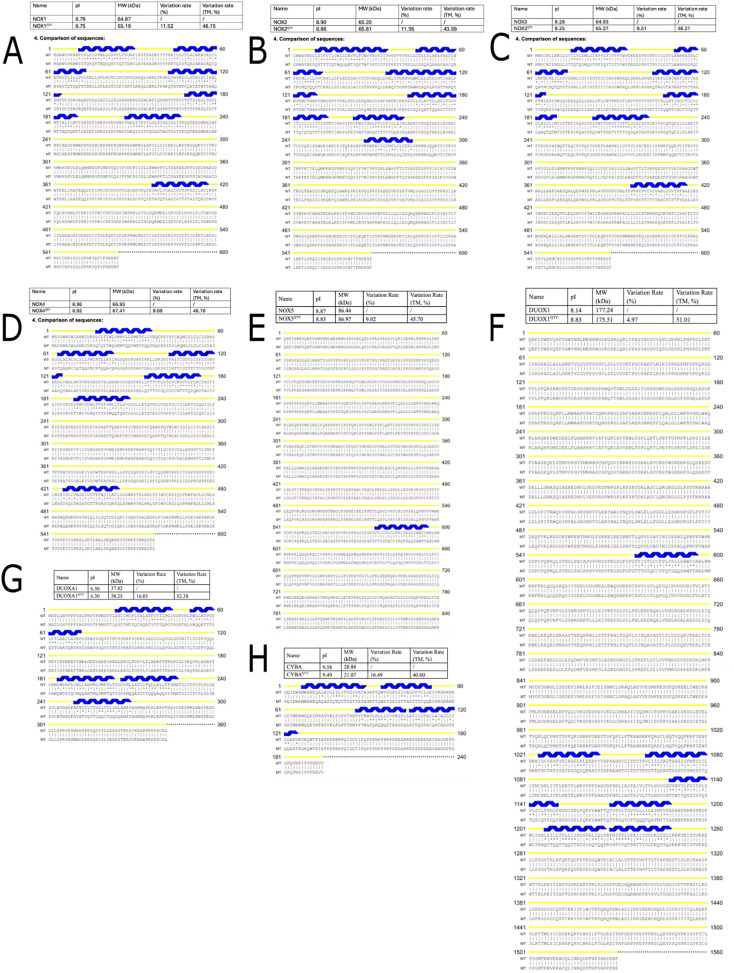
Protein sequence alignments of eight native NADPH oxidases with their QTY variants with reduced hydrophobicity. The symbols | and * indicate whether amino acids are identical or different, respectively. Q replaces L, T replaces V and I, and Y replaces **F.** The alpha helices (colored in blue) are shown above the protein sequences. The characteristics of native and QTY variants listed are isoelectric point (pI), molecular weight (MW), total variation % and transmembrane variation %. The alignments are **A)** NOX1 vs NOX1^QTY^, **B)** NOX2 vs NOX2^QTY^, **C)** NOX3 vs NOX3^QTY^, **D)** NOX4 vs NOX4^QTY^, **E)** NOX5 vs NOX5^QTY^, **F)** DUOX1 vs DUOX1^QTY^, **G)** DUOXA1 vs DUOXA1^QTY^, **H)** CYBA vs CYBA^QTY^. Compared to the native, the QTY variants show significant changes, ranging from 40.00% to 52.38%, in the TM region without significant changes in pI and MW.

The protein sequences for eight NADPH oxidases were aligned with their QTY variants (Fig 1, Figure S1, Figure S2, Figure S3 in S1 File). Overall amino acid sequence changes ranged from 4.97% to 16.49% (Fig 1, Table 1), while transmembrane region change ranges from 40.00% to 52.38% (Fig 1, Table 1). Notably, the protein isoelectric point (pI) remains roughly unchanged, as the substituted amino acids Q (glutamine), T (threonine), and Y (tyrosine) are all neutral amino acids, exerting no effect on the protein acidity or basicity.

**Table 1:** Protein characteristics of eight NADPH oxidases and their QTY variants.

Name	RMSD (Å)	pI	MW (kDa)	TM Variation (%)	Overall variation (%)	Intrinsic Solubility Score
NOX1^AF^	–	8.79	64.87	–	–	−3.61
NOX1^QTY^	0.53^*^	8.75	65.19	48.15	11.52	−0.88
NOX2^CryoEM^	–	8.90	65.20	–	–	−3.87
NOX2^QTY^	0.56	8.86	65.61	43.59	11.95	−0.87
NOX3^AF^	–	8.28	64.93	–	–	−3.84
NOX3^QTY^	0.59^*^	8.25	65.27	48.21	9.51	−1.33
NOX4^AF^	–	8.96	66.93	–	–	−3.70
NOX4^QTY^	0.46^*^	8.92	67.41	48.70	9.69	−1.02
NOX5^CryoEM^	–	8.87	86.44	–	–	−2.45
NOX5^QTY^	4.96	8.73	88.18	45.70	9.02	−0.05
DUOX1^CryoEM^	–	8.14	177.24	–	–	−3.00
DUOX1^QTY^	1.16	8.03	178.57	51.01	4.97	−1.27
DUOXA1^CryoEM^	–	6.30	37.82	–	–	−2.79
DUOXA1^QTY^	0.36	6.30	38.69	52.38	16.03	0.51
CYBA^CryoEM^	–	9.58	20.88	–	–	1.90
CYBA^QTY^	0.66	9.41	21.10	40.00	16.49	−0.40

* RMSD calculated against AlphaFold 3 predicted native structures due to the lack of experimentally determined CryoEm native structures. Abbreviations: pI - isoelectric point; MW – molecular weight; TM – transmembrane; RMSD – root-mean-square deviation; AF – AlphaFold 3; (-) – not applicable. The eight NADPH oxidases are listed in the same order as Fig 1. RMSDs were calculated after missing residues in the native AlphaFold/Cryo-EM structures were cut out. The QTY variants substitutions show significant change in the transmembrane (TM) region, ranging from 40.00% to 52.38%, while the overall structural changes are between 4.97% to 16.49%.

The molecular weight (MW) changes were minimal due to the similar masses of substituted amino acid pairs. Leucine (MW: 131.17 Da) is lighter than Q (MW: 146.14 Da), isoleucine (MW 131.17 Da) is slightly heavier than T (MW: 119.12 Da), valine (MW: 117.15 Da) is slightly lighter than T (MW: 119.12 Da), and phenylalanine (MW: 165.19 Da) is slightly lighter than Y (MW: 181.19 Da). These effects result in negligible overall molecular weight variations.

### Accuracy assessment in AlphaFold 3 predictions

AlphaFold 3 uses several metrics to measure its protein folding accuracy: predicted local distance difference test (pLDDT), predicted aligned matrix (PAE), and predicted template modeling score (pTM).

**Fig 2:**
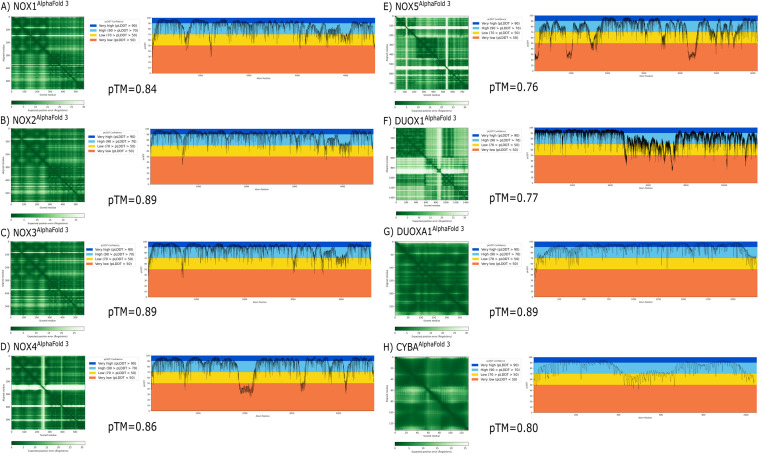
AlphaFold 3 predicted QTY variants with reduced hydrophobicity and pLDDT interval. pLDDT confidence profiles, PAE matrices, and pTM scores were generated for eight human transmembrane proteins. Predicted aligned error (PAE) matrices show predominantly low error (dark green), indicating high confidence in residue positioning, with few regions of increased error. Transmembrane regions were predicted with predominantly high to very high confidence (blue and dark blue shaded regions), while some regions show lower confidence (yellow to orange). In contrast to the others, NOX5 and DUOX1 display extended regions of reduced confidence, indicating increased structural uncertainty. Overall high pTM scores indicate high prediction accuracy and strong resemblance to known experimentally determined protein structures. **A)** NOX1^AlphaFold 3^, **B)** NOX2^AlphaFold 3^, **C)** NOX3^AlphaFold 3^, **D)** NOX4^AlphaFold 3^, **E)** NOX5^AlphaFold 3^, **F)** DUOX1^AlphaFold 3^, **G)** DUOXA1^AlphaFold 3^, **H)** CYBA^AlphaFold 3^.

pLDDT metric quantifies confidence in folding at individual residue positions [41]. The dark blue regions, where the pLDDT score > 90, represent highly confident folding. Blue regions, with pLDDT score < 90 and > 70, signify confident regions. The yellow regions, pLDDT < 70 and > 50, demonstrate moderate confidence in the prediction, and orange regions, pLDDT < 50, show low confidence. Our results reveal that transmembrane regions in QTY variants achieve high confidence predictions, with only few terminal ribbon-like unstructured loops exhibiting lower confidence scores. These unstructured loops were removed from the final models to enhance clarity (Fig 2).

PAE measures the error between each pair of amino acid residues [43] represented as a symmetrical matrix where rows and columns correspond to residue pairs. Diagonal elements reflect local confidence in residue placement. Darker green regions in the PAE matrix indicate low PAE values. Our results predominantly consist of darker green regions, with the exception occurring in unstructured loop regions (Fig 2).

pTM measures the overall protein folding quality by assessing the similarity between the true structure and the predicted structure [43]. pTM ranges from 0 to 1, in which 1 indicates a perfect structural alignment. Our results achieved an average pTM score of 0.84 (Fig 2), indicating high accuracy and strong resemblance to known experimentally determined protein structures.

### Superpositions of native NADPH oxidase structures and their AlphaFold 3 predicted QTY variants with reduced hydrophobicity

**Fig 3:**
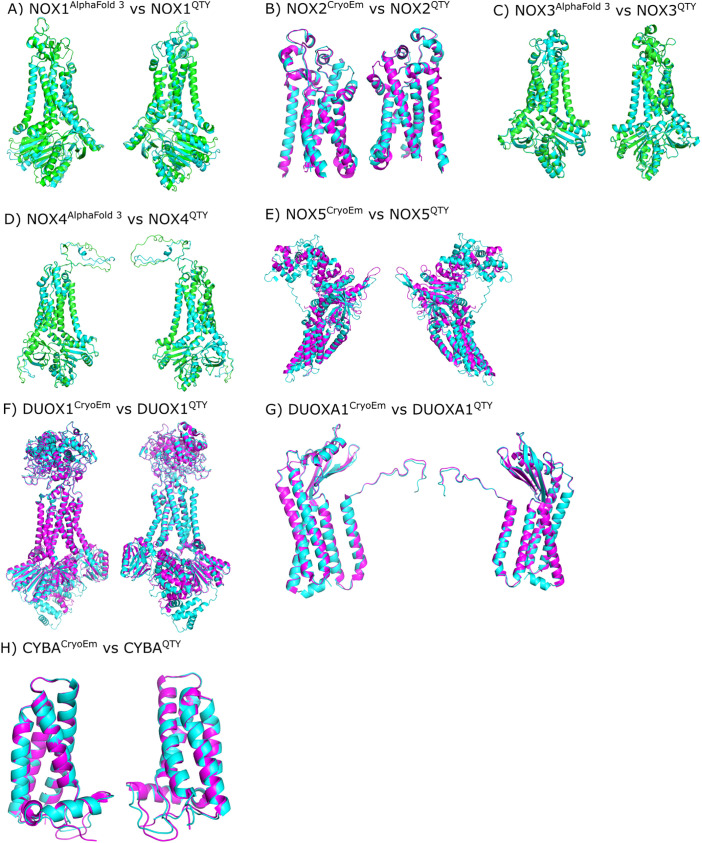
Superpositions of native NADPH oxidase structures and their AlphaFold 3 predicted QTY variants with reduced hydrophobicity. NADPH AlphaFold 3 predicted reduced hydrophobicity QTY variants were compared to their CryoEM determined native structures, when available. NOX1, NOX3, and NOX4 were superposed with their AlphaFold 3 predicted native structures because they lack experimentally determined CryoEm structures at the time of writing. AlphaFold 3 predicted native structures are shown in green, and CryoEM determined structures are shown in magenta, while their AlphaFold predicted QTY variants are displayed in cyan. All CryoEM structures were obtained from the Protein Data Bank (PDB). The similarities between structures as seen in the superpositions show that QTY variants and the native proteins have comparable structures. Unstructured loops in the AlphaFold 3 predicted variants have been removed. **A)** NOX1^AlphaFold 3^ vs NOX1^QTY^, **B)** NOX2^CryoEM^ vs NOX2^QTY^, **C)** NOX3^AlphaFold 3^ vs NOX3^QTY^, **D)** NOX4^AlphaFold 3^ vs NOX4^QTY^, **E)** NOX5^CryoEM^ vs NOX5^QTY^, **F)** DUOX1^CryoEM^ vs DUOX1^QTY^, **G)** DUOXA1^CryoEM^ vs DUOXA1^QTY^, **H)** CYBA^CryoEM^ vs CYBA^QTY^.

We superposed five experimentally determined CryoEM NADPH oxidase structures with their AlphaFold 3-predicted QTY analogs: NOX2 (PDB: 7U8G) [10], NOX5 (PDB: 8U85) [21], DUOX1 (PDB: 7D3E) [44], DUOXA1 (PDB: 7D3F) [44], CYBA (PDB: 8WEJ) [25]. NOX1, NOX3, and NOX4 were excluded due to the absence of experimentally determined structures at the time of writing. The superpositions of the transmembrane enzymes and their respective QTY variants are: NOX2^CryoEM^ vs NOX2^QTY^, NOX5^CryoEM^ vs NOX5^QTY^, DUOX1^CryoEM^ vs DUOX1^QTY^, DUOXA1^CryoEM^ vs DUOXA1^QTY^, and CYBA^CryoEM^ vs CYBA^QTY^ (Fig 3).

The QTY variants generally superposed well with their respective native counterparts. The RMSDs range from 0.36Å to 4.96Å, with all pairs except NOX5 vs NOX5^QTY^ and DUOX1 vs DUOX1^QTY^ achieving RMSD below 1Å (Table 1, Fig 1). Despite experiencing a significant percentage of amino acid substitutions (40.00%−52.38%) in the transmembrane region, the proteins maintained similar structures (Fig 3), confirming notable structural conservation between native NADPH oxidases and their predicted QTY variants with reduced hydrophobicity.

The elevated RMSD score for NOX5^CryoEM^ vs NOX5^QTY^ pair likely reflects reduced confidence in the AlphaFold 3 prediction. Specifically, residues R196-R222 and S678-S705 contain alpha helices with confidence scores between the moderate (50 < pLDDT < 70) and low (pLDDT < 50) ranges (Fig 2). Therefore, the high RMSD of NOX5 may be prone to an inaccurate AlphaFold 3 result.

To assess proteins that lacked experimental structures (NOX1, NOX3, NOX4), we superposed the AlphaFold 3 predicted native structures with their AlphaFold 3 predicted QTY variants with reduced hydrophobicity (Fig 3). As shown in Fig 3, the AlphaFold 3 predicted structures superpose very well. The visual evaluation is also supported by RMSD values: a) NOX1^Native^ vs NOX1^QTY^ (RMSD = 0.53 Å), c) NOX3^Native^ vs NOX3^QTY^ (RMSD = 0.59 Å), d) NOX4^Native^ vs NOX4^QTY^ (RMSD = 0.46 Å). These results confirm high structural similarity between native NADPH oxidases and their predicted QTY variants with reduced hydrophobicity.

### Superpositions of CryoEM structures with AlphaFold 3 predicted native structures and their QTY analogs with reduced hydrophobicity

**Fig 4:**
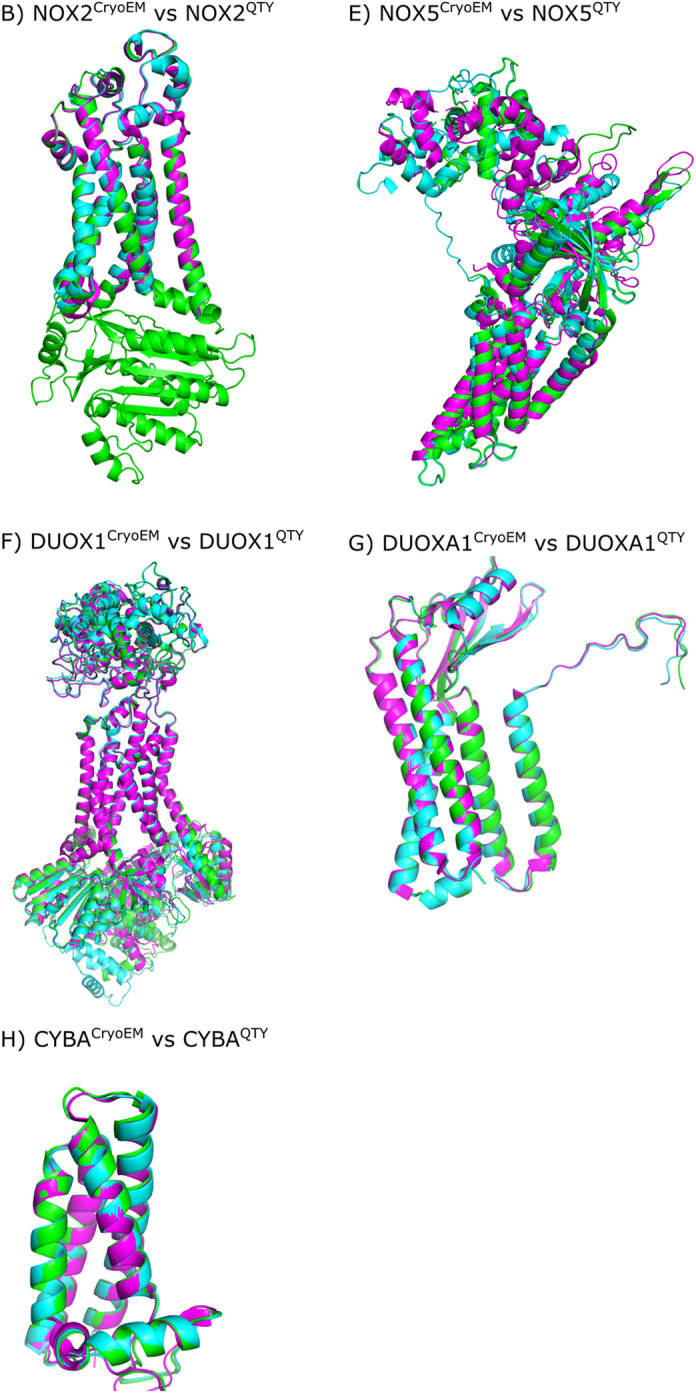
Superpositions of CryoEM structures with AlphaFold 3 predicted NADPH oxidases and their QTY variants with reduced hydrophobicity. Superpositions of i) the experimentally determined CryoEM native structures (magenta), **ii)** AlphaFold 3 predicted native structures (green), **iii)** AlphaFold 3 predicted QTY variants (cyan). These three different kinds of structures are superposed very well. Differences and variation are largely insignificant. The quality of superpositions demonstrates both the accuracy of AlphaFold 3 as well as the feasibility of utilizing reduced hydrophobicity QTY variants. NOX1, NOX3, and NOX4 are not displayed as they lack experimentally determined CryoEM structures at the time of writing. **A)** NOX2^CryoEM^ vs NOX2^QTY^, **B)** NOX5^CryoEM^ vs NOX5^QTY^, **C)** DUOX1^CryoEM^ vs DUOX1^QTY^, **D)** DUOXA1^CryoEM^ vs DUOXA1^QTY^, **E)** CYBA^CryoEM^ vs CYBA^QTY^.

We performed comprehensive superpositions incorporating i) the experimentally determined CryoEM native NADPH oxidases with ii) AlphaFold 3 predicted native NADPH oxidases and iii) AlphaFold 3 predicted QTY variants with reduced hydrophobicity. Excellent superpositions are demonstrated across all proteins except for NOX5, demonstrating both AlphaFold 3’s accurate predictions and the possible utility of QTY analogs with reduced hydrophobicity of NADPH oxidases in medicine and drug design (Fig 4). The low quality of the NOX5 alignment likely arises from the low folding confidence of the AlphaFold 3 model, highlighting the limitation that protein prediction models may not accurately reflect true protein structures. Accordingly, the QTY analog of NOX5 should be interpreted with caution, serving as a warrant case for future QTY analog investigation.

### Analysis of the hydrophobic surface of native NADPH oxidases and their QTY analogs with reduced hydrophobicity

**Fig 5:**
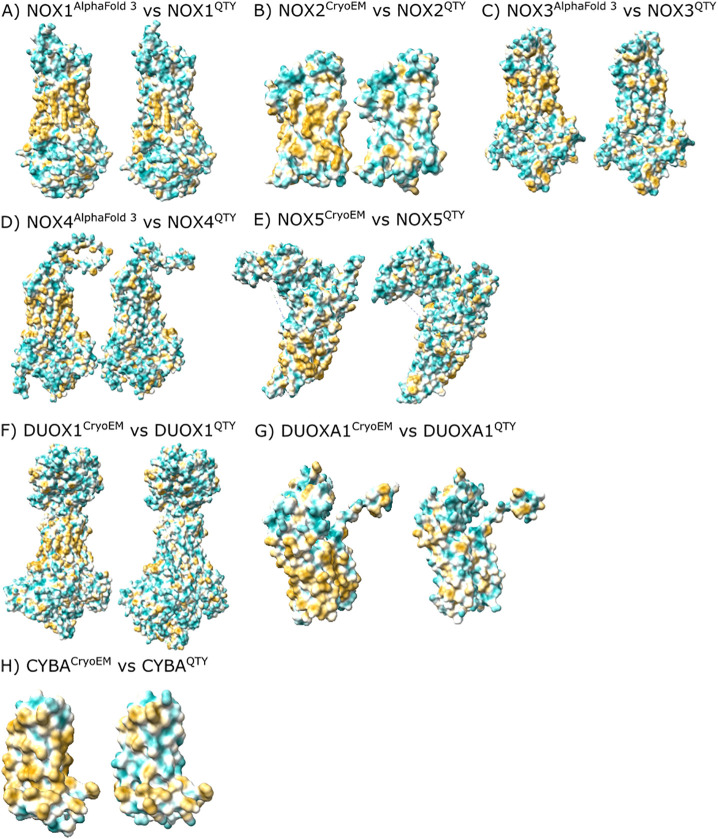
Hydrophobic surface of eight NADPH oxidases and their QTY variants with reduced hydrophobicity. The native NADPH oxidases have hydrophobic residues L, I, V, and F in the transmembrane regions. After applying the QTY code, the variant proteins have had these hydrophobic residues substituted for hydrophilic ones. Q replaces L, T replaces V and I, and Y replaces **F.** Hydrophobic regions (yellow) become hydrophilic (cyan) as a result of applying the QTY code. Unstructured loops have been removed for clarity of direct comparisons. **A)** NOX1^AlphaFold 3^ vs NOX1^QTY^, **B)** NOX2^CryoEM^ vs NOX2^QTY^, **C)** NOX3^Alpha Fold 3^ vs NOX3^QTY^, **D)** NOX4^AlphaFold 3^ vs NOX4^QTY^, **E)** NOX5^CryoEM^ vs NOX5^QTY^, **F)** DUOX1^CryoEM^ vs DUOX1^QTY^, **G)** DUOXA1^CryoEM^ vs DUOXA1^QTY^, **H)** CYBA^CryoEM^ vs CYBA^QTY^.

The eight transmembrane NADPH oxidases included in our study are hydrophobic and insoluble in water. Experimental characterization requires detergent-mediated isolation from lipid layers, in which the detergent disrupts the hydrophobic bonds that link the enzymes to the lipid bilayer. Without the proper detergents, proteins aggregate and precipitate, losing their biological functionality. Yellow-colored hydrophobic surfaces represent areas in the transmembrane domain that traverse the hydrophobic lipid bilayer (Fig 5). This region contains hydrophobic, nonpolar amino acids including Leucine (L), Isoleucine (I), Valine (V), Phenylalanine (F), Methionine (M), Tryptophan (W), and Alanine (A), which exclude water through lipid interactions.

QTY code, replacing hydrophobic residues (L, I/V, F) with hydrophilic, polar counterparts (Q, T, Y), significantly reduces hydrophobic surface areas. This is seen in the decreased yellow regions coupled and increase in blue regions exhibited in the QTY variants compared to their native structures (Fig 5). Importantly, the QTY code substitution also did not compromise the alpha-helical structures of the NADPH oxidases. This is consistent with findings from previous studies, notably one on QTY variants of chemokine and cytokine receptors that showed QTY analogs keeping their thermostabilities, ligand binding activities, and alpha-helical structures despite becoming hydrophilic [31].

**Fig 6:**
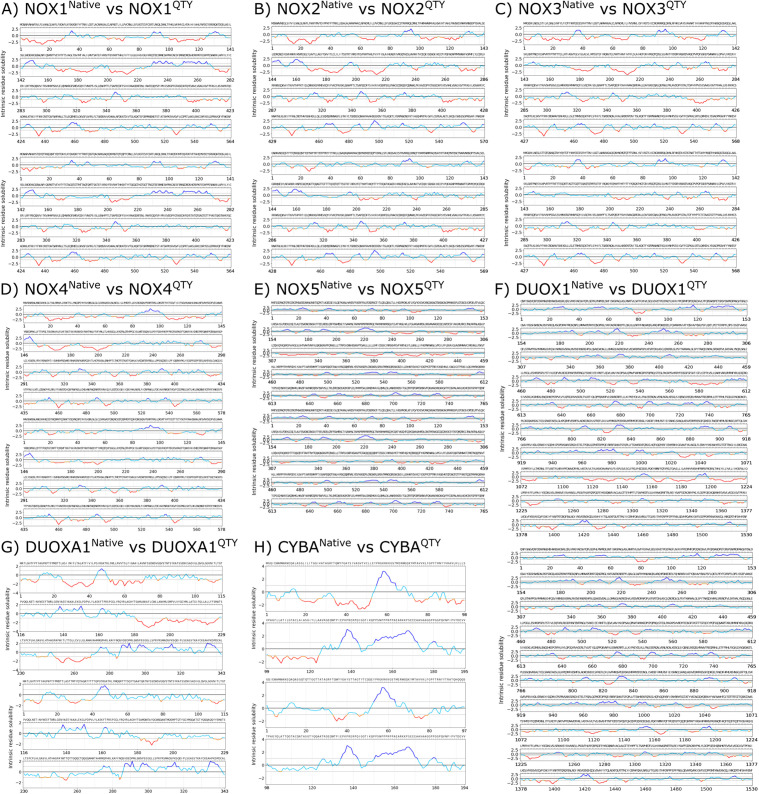
The CamSol intrinsic profiles of eight NADPH oxidases and their QTY analogs with reduced hydrophobicity. The red regions represent highly insoluble aggregation-prone regions of proteins, while blue regions represent highly soluble and aggregation-resistant regions. All eight proteins showed reduced red and increased blue regions after application of the QTY code, suggesting reduced hydrophobicity. **A)** NOX1^Native^ vs NOX1^QTY^, **B)** NOX2^Native^ vs NOX2^QTY^, **C)** NOX3^Native^ vs NOX3^QTY^, **D)** NOX4^Native^ vs NOX4^QTY^, **E)** NOX5^Native^ vs NOX5^QTY^, **F)** DUOX1^Native^ vs DUOX1^QTY^, **G)** DUOXA1^Native^ vs DUOXA1^QTY^, **H)** CYBA^Native^ vs CYBA^QTY^.

At each residue, CamSol intrinsic solubility profiles show a significant reduction in extended aggregation-prone regions across all eight proteins’ QTY analogs, as shown by fewer and shorter stretches of red sections with strongly negative CamSol scores and more stretches of blue sections with strongly positive CamSol scores (Fig 6). All eight proteins’ QTY analogs showed an increase in overall solubility score, suggesting reduced hydrophobicity. This shift towards positive intrinsic solubility values demonstrates a reduced propensity for hydrophobic-driven aggregation and improved compatibility with aqueous environments.

### AlphaFold 3 predictions

This study relied extensively on AlphaFold 3, released by DeepMind in May 2024. Unlike AlphaFold 2, this advanced model predicts diverse biomolecules besides proteins including ligands, ions, modified residues and nucleic acids [33]. AlphaFold 3 outperforms traditional docking methods such as Vina and RoseTTAFold All-Atom [33].

The AlphaFold 3 server (https://alphafoldserver.com) provides free, fast, and user-friendly access with comprehensive guidance for protein folding prediction and analysis. All our QTY analogs were predicted using this platform.

Despite being a powerful tool, AlphaFold 3 exhibits limitations. The model occasionally suffers from hallucinations–plausible but nonexistent structures [33]. Specifically in this study, the low confidence of NOX5^QTY^ Alpha Fold 3 folding made proceeding superposition analysis challenging. While hallucination is less frequent compared to AlphaFold 2, the issue pertains. Additionally, AlphaFold 3 struggles with intrinsically disordered regions and multi-state conformations [45]. Many proteins exist in multiple conformation or lack stable forms under specific physiological conditions [46,47]. We look for the AlphaFold 4 release in near future that may address the limitations.

In our result, AlphaFold 3 produces generally high pLDDT values, suggesting confident predictions. However, since pLDDT derives from training data, applicability under actual biological or experimental conditions may vary [48]. As a result, while AlphaFold 3 provides useful structural insights, experimental validation remains crucial for confirming computational predictions.

### Transmembrane NADPH oxidases investigated in this study

We investigated QTY analogs across the NADPH oxidase family and proteins supporting the oxidases’ structure and function. Results demonstrate QTY code efficacy through excellent structural superpositions between native structures and their QTY analogs. The eight studied proteins (NOX1, NOX2, NOX3, NOX4, NOX5, DUOX1, DUOXA1, and CYBA) all show medical relevance. While each homologue has a unique biological role, the NADPH oxidase family collectively regulates ROS production and impacts cancer progression.

The QTY analogs predicted in this study with reduced hydrophobicity offer several potential applications. Firstly, the QTY analogs can be purified in large quantities, facilitating drug design. Additionally, our results show a generalized pipeline for analyzing and implementing QTY code across diverse transmembrane proteins.

## Conclusion

Natural proteins can be divided into two distinct classes: hydrophilic proteins (Class I) and hydrophobic proteins (Class II) [49]. Their constituent helices divide into three chemically distinct yet structurally similar types: i) the type I alpha helix is hydrophilic and highly water-soluble, containing hydrophilic amino acids D, E, N, Q, K, R, S, T, and Y, typically found in water-soluble proteins, ii) the type II alpha helix is hydrophobic and often spans across membranes, containing hydrophobic amino acids L, I, V, F, M, P, W, and A, and is often found in the transmembrane segments of membrane proteins, iii) the type III alpha helix is composed of nearly equal amounts of hydrophobic and hydrophilic amino acids, which are often separated into hydrophobic and hydrophilic faces [50].

In this study, we successfully applied the QTY code to eight NADPH oxidase transmembrane proteins, generating QTY analogs with reduced hydrophobicity. We employed AlphaFold 3 to predict the QTY variant structure, performed structural superposition with the native NADPH oxidase structure, and conducted bioinformatic analysis of structural properties and hydrophobicity.

Our analyses suggest that QTY analogs exhibit overall structural similarity to their native proteins in AlphaFold 3 predicted models despite various amino acid substitutions. Application of the QTY code to NADPH oxidases markedly reduced predicted hydrophobic surface regions while largely preserving the global fold. These results indicate that hydrophilic variants of NADPH oxidases could potentially serve as soluble surrogates for structural and functional studies, particularly in aqueous environments where native membrane proteins present experimental challenges. However, because these observations rely on computational structure predictions, experimental validation will be required to determine whether the QTY variants maintain structural stability and functional activity.

Beyond basic research applications, reduced hydrophobicity QTY variants may offer a potential approach for generating soluble models of membrane proteins that could assist future studies in biotechnology or drug discovery. If experimentally validated, such hydrophilic variants could potentially aid efforts such as antibody discovery or the design of inhibitors targeting disease-associated NOX activity, helping to circumvent some experimental challenges associated with native membrane-bound forms.

Future studies should examine whether QTY analogs can maintain their interaction with other proteins successfully despite reduced hydrophobicity. For instance, NOX2’s interaction with p22 relies on hydrophobic electrostatic forces [10]. The balance between preserving the original structure and interaction, and reducing hydrophobicity for experimental purposes, will be of interest in future studies. Additionally, different protein folding tools can be used to validate AlphaFold 3’s prediction of the QTY analog to mitigate its hallucination effects. For instance, D-I-TASSER, a deep learning model building on top of the threading model I-TASSER, has been shown to outperform AlphaFold 3 and AlphaFold 2 in CASP protein prediction [49].

Overall, our findings show that QTY protein analogs maintain high structural similarity to the native protein structures. The QTY code successfully reduced the hydrophobicity of transmembrane proteins while maintaining their original structure. These NADPH oxidase QTY analogs may serve useful for cancer treatment monitoring via ROS control.

## Methods

### The rationale of the QTY code

Purifying transmembrane proteins presents significant challenges, requiring detergents and extensive time investment. The QTY code stems from the structural similarity in the density maps of amino acids Q and L, T and I/V, and Y and F [31]. This code significantly reduces the hydrophobicity of transmembrane proteins through substantial amino acid substitutions.

The QTY code aims to engineer transmembrane proteins into their analogs with reduced hydrophobicity by substituting hydrophobic amino acids with hydrophilic ones: L with Q, I and V with T, and F with Y [32]. Despite extensive sequence modification in the eight transmembrane proteins, their QTY variants maintain similar pI and molecular weights (MW) (Table 2).

**Table 2:** Characteristics of the eight human NADPH oxidases in this study.

Name (Uniprot ID)	Structure (Å, PDB ID)	Tissue Expression	Medical Relevance
NOX1 (Q9Y5S8)	AlphaFold 3 Predicted	Colon	Superoxide production; Crohn’s disease, ulcerative colitis
NOX2 (p04839)	CryoEM (3.20 Å, 7U8G)	Phagocytes	Superoxide production
NOX3 (Q9HBY0)	AlphaFold 3 Predicted	Inner ear	Otoconia morphogenesis
NOX4 (Q9NPH5)	AlphaFold 3 Predicted	Ubiquitous	H_2_O_2_ production
NOX5 (Q96PH1)	CryoEM (3.20 Å, 8U85)	Spermatocytes, spleen, lymph nodes	ROS production
DUOX1 (Q9NRD9)	CryoEM (2.30 Å, 7D3F)	Thyrocytes, epithelial cells	H_2_O_2_ production, innate immunity
DUOXA1 (Q1HG43)	CryoEM (2.30 Å, 7D3F)	Thyroid gland	DUOX maturation
CYBA (P13498)	CryoEM (2.79 Å, 8WEJ)	Granulocyte	Superoxide production

The NADPH oxidase protein names, Uniprot IDs, and CryoEM structure in Å with PDB ID are listed when applicable. The lists of tissue location, medical relevance, and function are not exhaustive.

### Protein sequences and other characteristics

The native protein sequences for the studied NOX enzymes NOX1, NOX2, NOX3, NOX4, NOX5, DUOX1, DUOXA1, and CYBA were obtained from UniProt (https://www.uniprot.org). The QTY code was applied to the native proteins using the Protein Solubilizing Server (https://pss.sjtu.edu.cn/). The MWs, pI values, TM variation, overall variation, and sequence alignments for the proteins are computed via Expasy (https://web.expasy.org/compute_pi/).

### AlphaFold 3

The protein structures NOX1^QTY^, NOX2^QTY^, NOX3^QTY^, NOX4^QTY^, NOX5^QTY^, DUOX1^QTY^, DUOXA1^QTY^, and CYBA^QTY^ were predicted using AlphaFold 3 (https://alphafoldserver.com). First, the QTY sequence was generated from the Protein Solubilizing Server described above and then entered into AlphaFold 3 to generate the predicted structure. QTY protein analog structures are predicted using the AlphaFold 3 Server v3.0.1. Each prediction is run in the monomer mode, using the server’s default MSA generation and recycle settings. Each prediction is run with one random seed (NOX1 = 740489916; NOX2 = 153345029; NOX3 = 1088284846; NOX4 = 2115711105; NOX5 = 1970948703, DUOX1 = 1666590488; DUOXA1 = 393896960; CYBA = 1060114576). Structure templates are enabled, and no additional user-defined parameters are applied. We have uploaded our QTY-designed FASTA sequences as well as our AlphaFold 3 output data and predicted structure files to a GitHub repository for reproducibility purposes. The sequences and files can be accessed at this link: https://github.com/rickhcheng/qty_proteins.

### Superposed structures

Native structures for proteins lacking an experimentally determined structure in this study (NOX1, NOX3, NOX4) were generated using the AlphaFold 3 model (https://alphafoldserver.com). Native structures for proteins with experimentally determined structures were obtained from the PDB. NOX2 (PDB: 7U8G), NOX5 (PDB:8U85), DUOX1 (PDB:7D3E), DUOXA1 (PDB:7D3F), and CYBA (PDB:8WEJ). QTY variant structures were also predicted using AlphaFold 3 (https://alphafoldserver.com). The structures were then superposed with the structure of the native protein and the RMSDs were calculated using PyMOL (https://pymol.org/).

### Structure visualization

Superpositions between the QTY variants and their native protein structures were done using PyMOL (https://pymol.org/). Protein hydrophobicity figures were generated using UCSF Chimera (https://www.rbvi.ucsf.edu/chimera/).

### Hydrophobicity Analysis

CamSol Intrinsic prediction (https://www-cohsoftware.ch.cam.ac.uk/index.php/camsolintrinsic) is used to assess proteins’ intrinsic solubility and aggregated propensity. One score will be assigned to each residue, creating a solubility profile where scores higher than 1 denote highly soluble regions; scores lower than –1 denote poorly soluble ones. An overall solubility score will be assigned, where higher scores indicate better solubility.

## Supporting information

S1 File The Support information provides: 1) enlarged individual alignment of each membrane proteins from figure 1 so readers can see clearly the sequence alignment, 2) the 2D topology of each membrane protein and the transmembrane domain of alpha-helices, 3) bioinformatics of membrane protein hydrophobicity before and after application of the QTY code. The Support figures further provides information on how QTY code works and why is works. These figures provide readers better understanding the QTY code. **S1 Fig. The enlarged protein sequence alignments of eight native NADPH oxidases with their water-soluble QTY variants from Figure 1.** The symbols | and * indicate whether amino acids are identical or different, respectively. Q replaces L, T replaces V and I, and Y replaces F. The alpha helices (colored in blue) are shown above the protein sequences. The characteristics of native and QTY variants listed are isoelectric focusing (pI), molecular weight (MW), total variation % and transmembrane variation %. The alignments are a) NOX1 vs NOX1^QTY^, b) NOX2 vs NOX2^QTY^, c) NOX3 vs NOX3^QTY^, d) NOX4 vs NOX4^QTY^, e) NOX5 vs NOX5^QTY^, f) DUOX1 vs DUOX1^QTY^, g) DUOXA1 vs DUOXA1^QTY^, h) CYBA vs CYBA^QTY^. Compared to the native, the QTY variants show significant changes, ranging from 40.00% to 52.38%, in the TM region without significant changes in pI and MW. **S2 Fig. Membrane topology of eight NADPH oxidases.** Topological structures were generated via Protter. The cell membrane is colored orange boarded by black lines. Topologies include: a) NOX1, b) NOX2, c) NOX3, d) NOX4, e) NOX5, f) DUOX1, g) DUOXA1, h) CYBA. **S3 Fig. Transmembrane helix predictions of native and QTY analogs.** Predictions were generated using DeepTMHMM. Predictions include a) NOX1^Native^ vs NOX1^QTY^, b) NOX2^Native^ vs NOX2^QTY^, c) NOX3^Native^ vs NOX3^QTY^, d) NOX4^Native^ vs NOX4^QTY^, e) NOX5^Native^ vs NOX5^QTY^, f) DUOX1^Native^ vs DUOX1^QTY^, g) DUOXA1^Native^ vs DUOXA1^QTY^, h) CYBA^Native^ vs CYBA^QTY^.
(PDF)
