# Supplementary material for: Structural bioinformatic studies of eight integral transmembrane NADPH oxidases and their AlphaFold 3 predicted QTY analogs with reduced hydrophobicity
Source: PLoS One. 2026 Jun 10;21(6):e0347525. doi: 10.1371/journal.pone.0347525 (PMC13252790; doi:10.1371/journal.pone.0347525)
Supplement: S1 File — The Support figures further provides information on how QTY code works and why is works. These figures provide readers better understanding the QTY code. S1 Fig. The enlarged protein sequence alignments of eight native NADPH oxidases with their water-soluble QTY variants from Figure 1. The symbols | and * indicate whether amino acids are identical or different, respectively. Q replaces L, T replaces V and I, and Y replaces F. The alpha helices (colored in blue) are shown above the protein sequences. The characteristics of native and QTY variants listed are isoelectric focusing (pI), molecular weight (MW), total variation % and transmembrane variation %. The alignments are a) NOX1 vs NOX1QTY, b) NOX2 vs NOX2QTY, c) NOX3 vs NOX3QTY, d) NOX4 vs NOX4QTY, e) NOX5 vs NOX5QTY, f) DUOX1 vs DUOX1QTY, g) DUOXA1 vs DUOXA1QTY, h) CYBA vs CYBAQTY. Compared to the native, the QTY variants show significant changes, ranging from 40.00% to 52.38%, in the TM region without significant changes in pI and MW. S2 Fig. Membrane topology of eight NADPH oxidases. Topological structures were generated via Protter. The cell membrane is colored orange boarded by black lines. Topologies include: a) NOX1, b) NOX2, c) NOX3, d) NOX4, e) NOX5, f) DUOX1, g) DUOXA1, h) CYBA. S3 Fig. Transmembrane helix predictions of native and QTY analogs. Predictions were generated using DeepTMHMM. Predictions include a) NOX1Native vs NOX1QTY, b) NOX2Native vs NOX2QTY, c) NOX3Native vs NOX3QTY, d) NOX4Native vs NOX4QTY, e) NOX5Native vs NOX5QTY, f) DUOX1Native vs DUOX1QTY, g) DUOXA1Native vs DUOXA1QTY, h) CYBANative vs CYBAQTY. (PDF) [file pone.0347525.s001.pdf]

Supporting Information

PONE-D-25-44849R2 - [EMID:2252d8002e2e4629]

Tutu Hu, Rick Cheng, Edward Chen, Shuguang Zhang

The Supporting Information Caption: The Support information provides: 1) enlarged individual alignment of each membrane proteins from figure 1 so readers can see clearly the sequence alignment, 2) the 2D topology of each membrane protein and the transmembrane domain of alpha-helices, 3) bioinformatics of membrane protein hydrophobicity before and after application of the QTY code. The Support figures further provides information on how QTY code works and why is works. These figures provide readers better understanding the QTY code.

a) NOX1 vs NOX1^{QTY}

Name	pI	MW (kDa)	Variation rate (%)	Variation rate (TM, %)
NOX1	8.79	64.87	/	/
NOX1^{QTY}	8.74	65.22	11.88	50.38

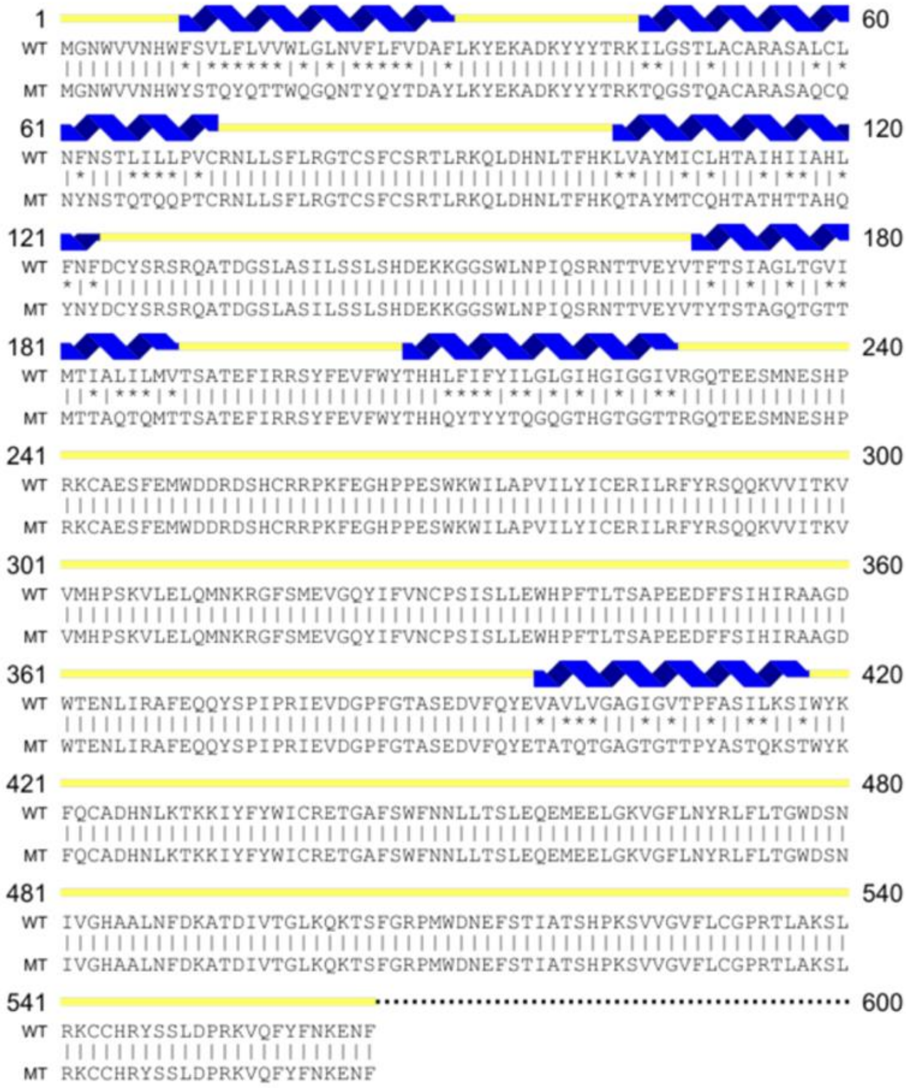

b) NOX2 vs NOX2^{QTY}

Name	pI	MW (kDa)	Variation rate (%)	Variation rate (TM, %)
NOX2	8.90	65.20	/	/
NOX2^{QTY}	8.86	65.61	11.95	43.59

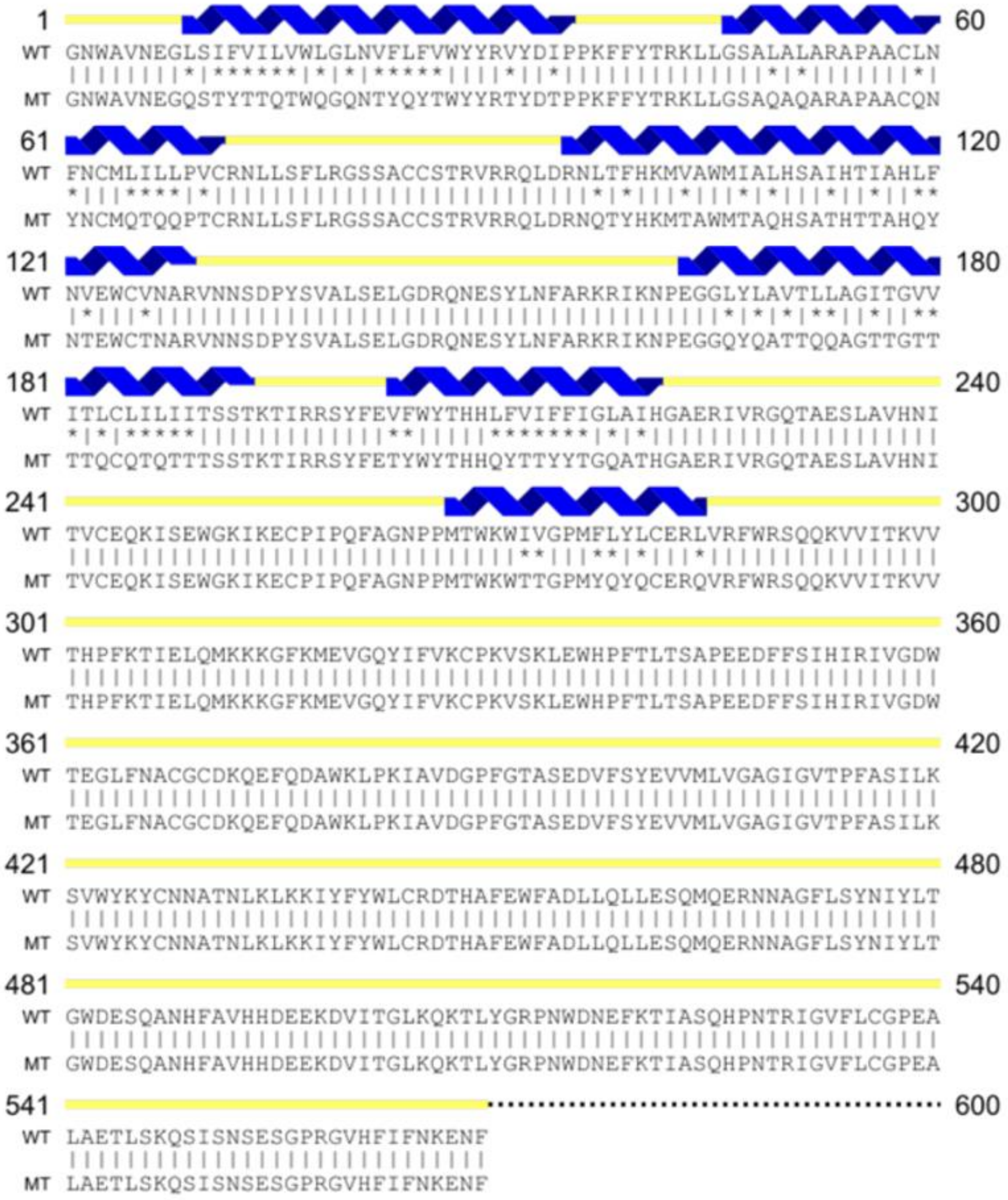

c) NOX3 vs NOX3^{QTY}

Name	pI	MW (kDa)	Variation rate (%)	Variation rate (TM, %)
NOX3	8.28	64.93	/	/
NOX3^{QTY}	8.24	65.27	10.56	47.62

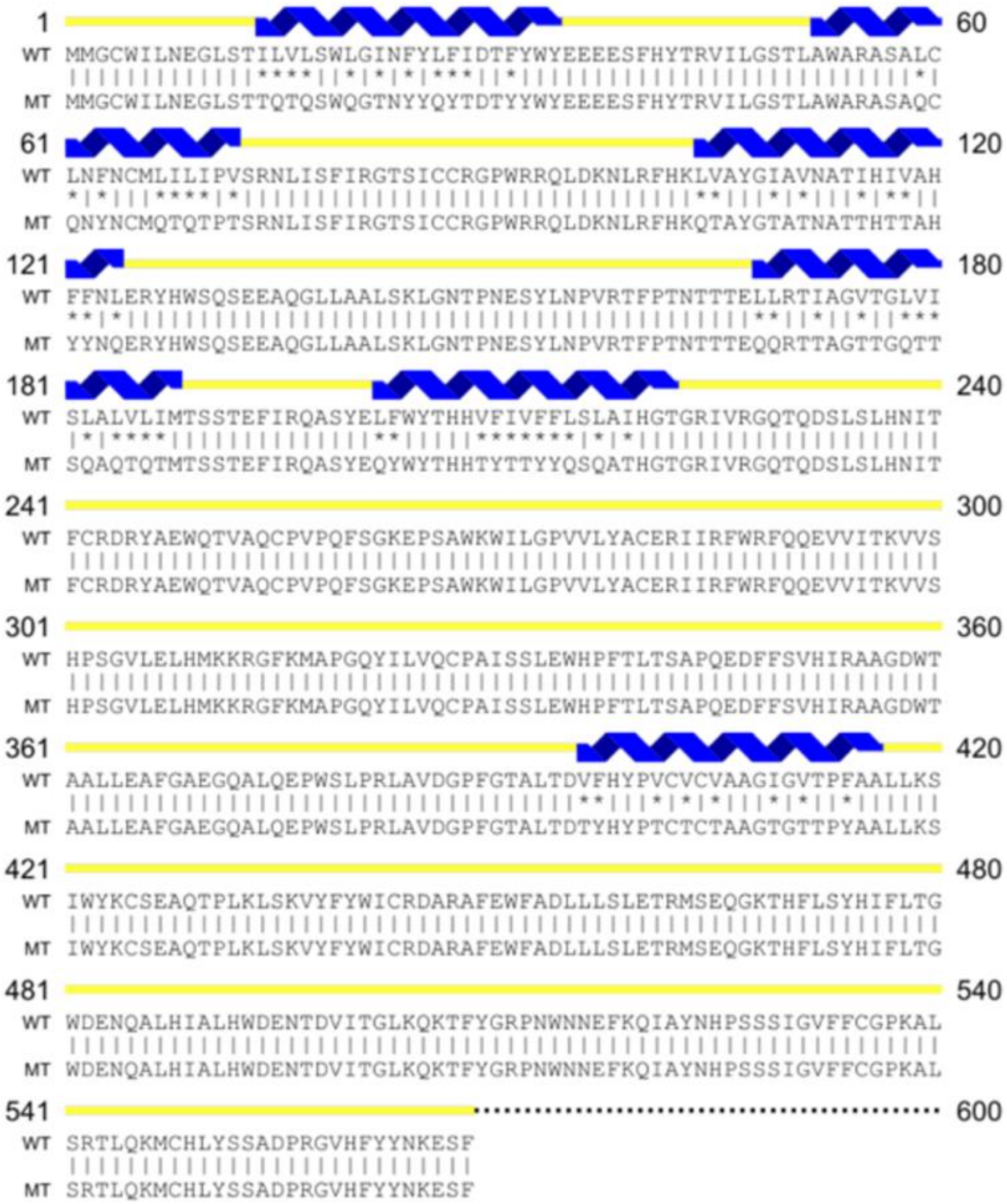

d) NOX4 vs NOX4^{QTY}

Name	pI	MW (kDa)	Variation rate (%)	Variation rate (TM, %)
NOX4	8.96	66.93	/	/
NOX4^{QTY}	8.91	67.45	10.90	50.00

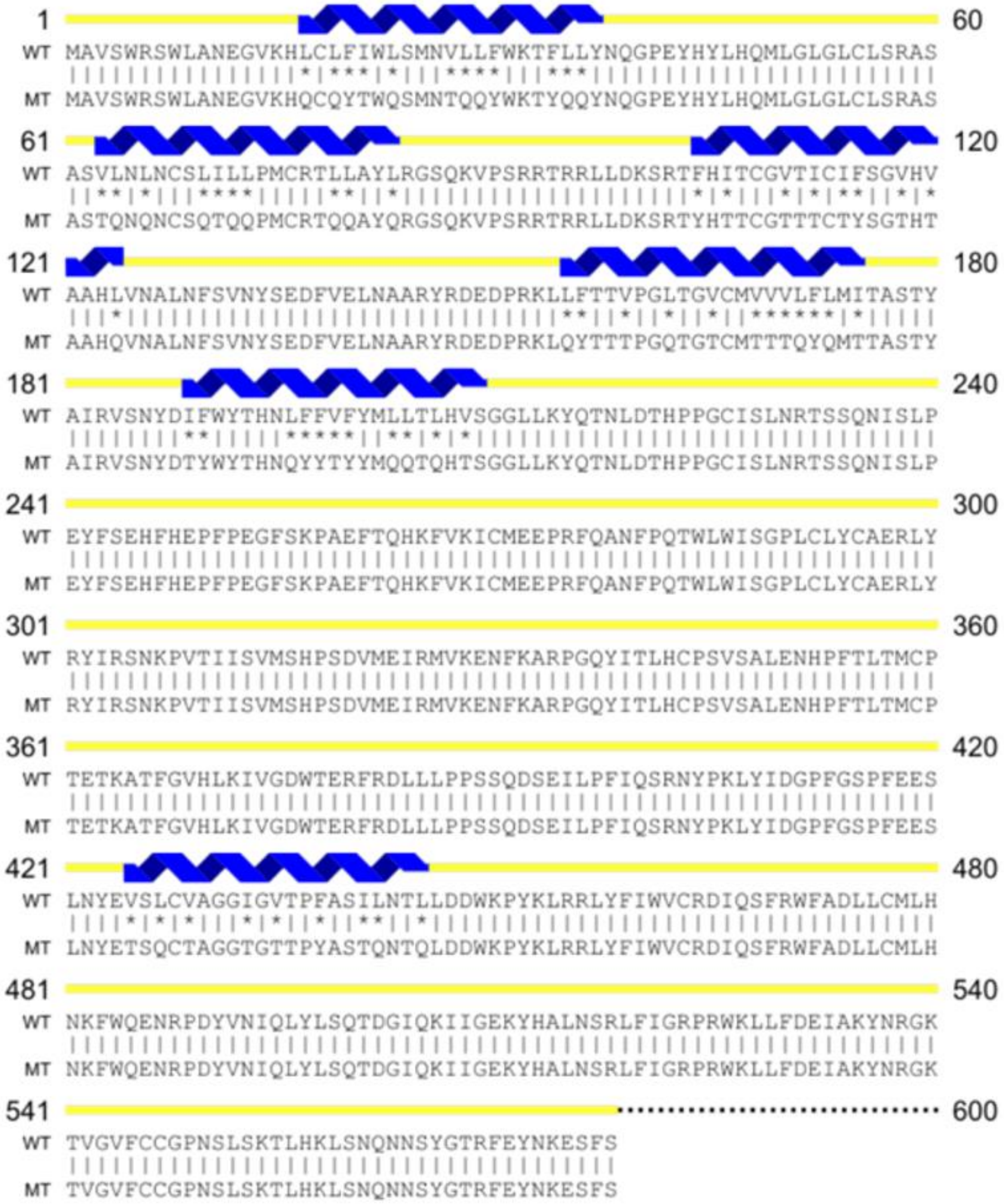

e) NOX5 vs NOX5^{QTY}

Name	pI	MW (kDa)	Variation Rate (%)	Variation Rate (TM, %)
NOX5	8.87	86.44	/	/
NOX5^{QTY}	8.83	86.97	9.02	45.70

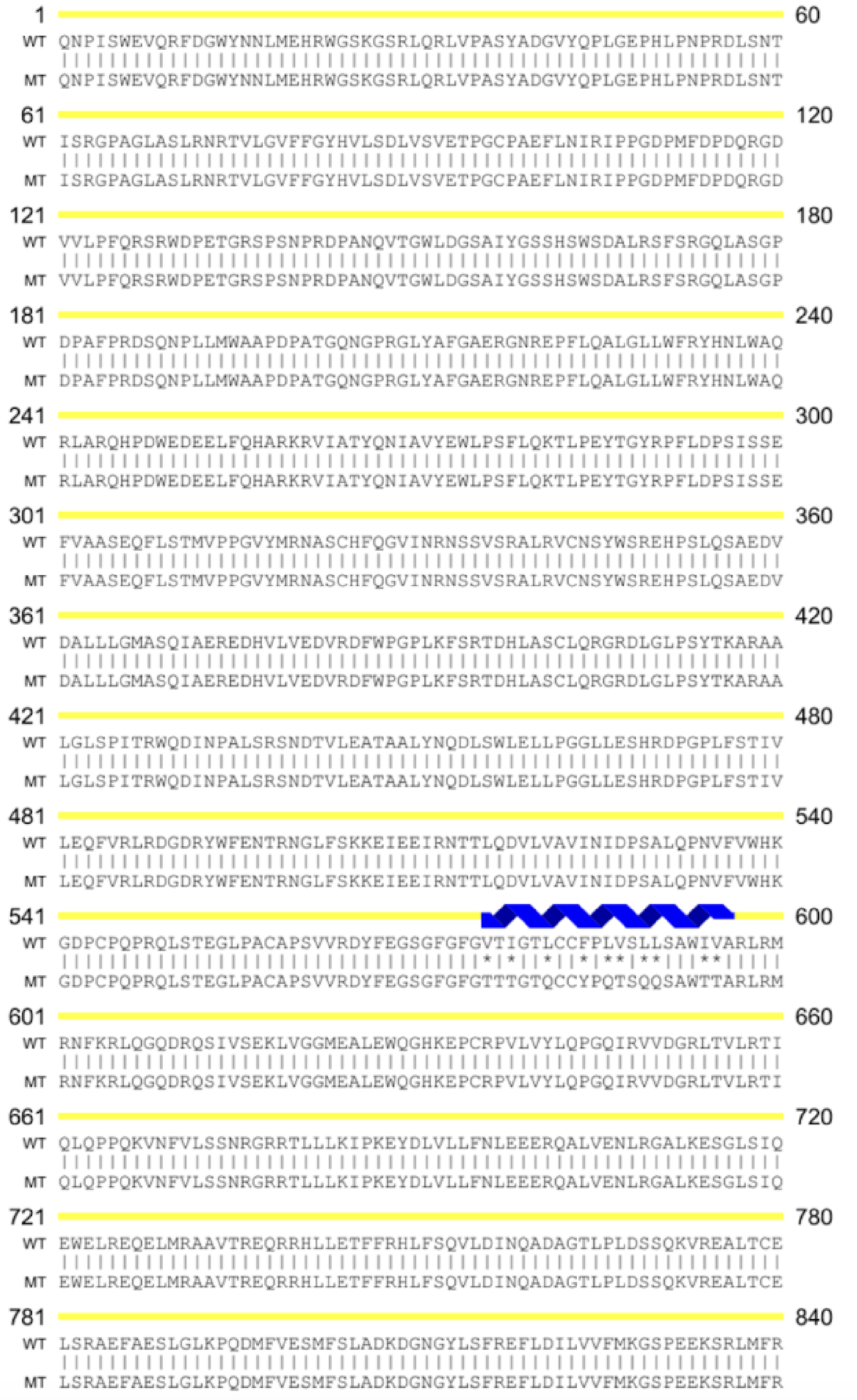

f) DUOX1 vs DUOX1^{QTY}

Name	pI	MW (kDa)	Variation Rate (%)	Variation Rate (TM, %)
DUOX1	8.14	177.24	/	/
DUOX1^{QTY}	8.83	175.51	4.97	51.01

```
1                                                                   60
WT ONPISWEVQRFDGWYNNLMEHRWGSKGSRLQRLVPASYADGVYQPLGEPHLPNPRDLSNT
   ||||||||||||||||||||||||||||||||||||||||||||||||||||||||||||
MT QNPISWEVQRFDGWYNNLMEHRWGSKGSRLQRLVPASYADGVYQPLGEPHLPNPRDLSNT
61                                                                 120
WT ISRGPAGLASLRNRTVLGVFFGYHVLSDLVSVETPGCPAEFLNIRIPPGDPMFDPDQRGD
   ||||||||||||||||||||||||||||||||||||||||||||||||||||||||||||
MT ISRGPAGLASLRNRTVLGVFFGYHVLSDLVSVETPGCPAEFLNIRIPPGDPMFDPDQRGD
121                                                                180
WT VVLPFQRSRWDPETGRSPSNPRDPANQVTGWLDGSAIYGSSHSWSDALRSFSRGQLASGP
   ||||||||||||||||||||||||||||||||||||||||||||||||||||||||||||
MT VVLPFQRSRWDPETGRSPSNPRDPANQVTGWLDGSAIYGSSHSWSDALRSFSRGQLASGP
181                                                                240
WT DPAFPRDSQNPLLMWAAPDPATGQNGPRGLYAFGAERGNREPFLQALGLLWFRYHNLWAQ
   ||||||||||||||||||||||||||||||||||||||||||||||||||||||||||||
MT DPAFPRDSQNPLLMWAAPDPATGQNGPRGLYAFGAERGNREPFLQALGLLWFRYHNLWAQ
241                                                                300
WT RLARQHPDWEDEELFQHARKRVIATYQNIAVYEWLPSFLQKTLPEYTGYRPFLDPSISSE
   ||||||||||||||||||||||||||||||||||||||||||||||||||||||||||||
MT RLARQHPDWEDEELFQHARKRVIATYQNIAVYEWLPSFLQKTLPEYTGYRPFLDPSISSE
301                                                                360
WT FVAASEQFLSTMVPPGVYMRNASCHFQGVINRNSSVSRALRVCNSYWSREHPSLQSAEDV
   ||||||||||||||||||||||||||||||||||||||||||||||||||||||||||||
MT FVAASEQFLSTMVPPGVYMRNASCHFQGVINRNSSVSRALRVCNSYWSREHPSLQSAEDV
361                                                                420
WT DALLLGMASQIAEREDHVLVEDVRDFWPGPLKFSRTDHLASCLQRGRDLGLPSYTKARAA
   ||||||||||||||||||||||||||||||||||||||||||||||||||||||||||||
MT DALLLGMASQIAEREDHVLVEDVRDFWPGPLKFSRTDHLASCLQRGRDLGLPSYTKARAA
421                                                                480
WT LGLSPITRWQDINPALSRSNDTVLEATAALYNQDLSWLELLPGGLLESHRDPGPLFSTIV
   ||||||||||||||||||||||||||||||||||||||||||||||||||||||||||||
MT LGLSPITRWQDINPALSRSNDTVLEATAALYNQDLSWLELLPGGLLESHRDPGPLFSTIV
481                                                                540
WT LEQFVRLRDGDRYWFENTRNGLFSKKEIEEIRNTTLQDVLVAVINIDPSALQPNVFVWHK
   ||||||||||||||||||||||||||||||||||||||||||||||||||||||||||||
MT LEQFVRLRDGDRYWFENTRNGLFSKKEIEEIRNTTLQDVLVAVINIDPSALQPNVFVWHK
541                                                                600
WT GDPCPQPRQLSTEGLPACAPSVVRDYFEGSGFGFGVTIGTLCCFPLVSLLSAWIVARLRM
   |||||||||||||||||||||||||||||||||||*|*||*||*|**|**|||**|||||
MT GDPCPQPRQLSTEGLPACAPSVVRDYFEGSGFGFGTTTGTQCCYPQTSQQSAWTTARLRM
601                                                                660
WT RNFKRLQGQDRQSIVSEKLVGGMEALEWQGHKEPCRPVLVYLQPGQIRVVDGRLTVLRTI
   ||||||||||||||||||||||||||||||||||||||||||||||||||||||||||||
MT RNFKRLQGQDRQSIVSEKLVGGMEALEWQGHKEPCRPVLVYLQPGQIRVVDGRLTVLRTI
661                                                                720
WT QLQPPQKVNFVLSSNRGRRTLLLKIPKEYDLVLLFNLEEERQALVENLRGALKESGLSIQ
   ||||||||||||||||||||||||||||||||||||||||||||||||||||||||||||
MT QLQPPQKVNFVLSSNRGRRTLLLKIPKEYDLVLLFNLEEERQALVENLRGALKESGLSIQ
721                                                                780
WT EWELREQELMRAAVTREQRRHLLETFFRHLFSQVLDINQADAGTLPLDSSQKVREALTCE
   ||||||||||||||||||||||||||||||||||||||||||||||||||||||||||||
MT EWELREQELMRAAVTREQRRHLLETFFRHLFSQVLDINQADAGTLPLDSSQKVREALTCE
781                                                                840
WT LSRAEFAESLGLKPQDMFVESMFSLADKDGNGYLSFREFLDILVVFMKGSPEEKSRLMFR
   ||||||||||||||||||||||||||||||||||||||||||||||||||||||||||||
MT LSRAEFAESLGLKPQDMFVESMFSLADKDGNGYLSFREFLDILVVFMKGSPEEKSRLMFR
```

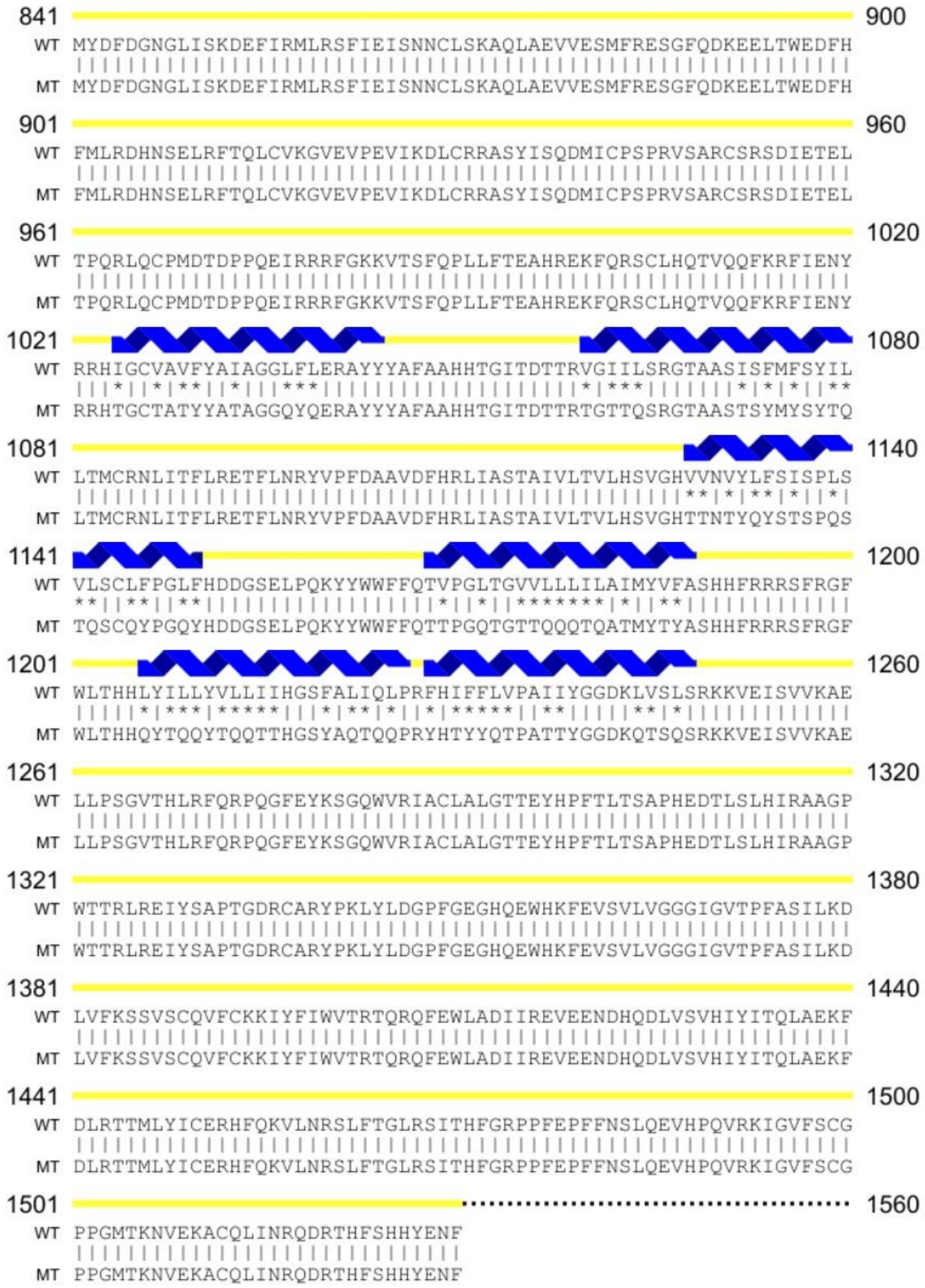

841 900
WT MYDFDGNGLISKDEFIRMLRSFIEISNNCLSKAQLAEVVESMFRESGFQDKEELTWEDFH
MT MYDFDGNGLISKDEFIRMLRSFIEISNNCLSKAQLAEVVESMFRESGFQDKEELTWEDFH
901 960
WT FMLRDHNSELRFTQLCVKGVEVPEVIKDLCRRASYISQDMICPSPRVSARCSRSDIETEL
MT FMLRDHNSELRFTQLCVKGVEVPEVIKDLCRRASYISQDMICPSPRVSARCSRSDIETEL
961 1020
WT TPQRLQCPMDTDPPQEIRRRFGKKVTSFQPLLFTEAHREKFQRSCLHQTVQQFKRFIENY
MT TPQRLQCPMDTDPPQEIRRRFGKKVTSFQPLLFTEAHREKFQRSCLHQTVQQFKRFIENY
1021 1080
WT RRHIGCVAVFYAIAGGLFLERAYYYAFAAHHTGITDTTRVGIILSRGTAASISFMFSYIL
MT RRHTGCTATYYATAGGQYQERAYYYAFAAHHTGITDTTRTGTTQSRGTAASTSYMYSYTQ
1081 1140
WT LTMCRNLITFLRETFLNRYVPFDAAVDFHRLIASTAIVLTVLHSVGHVVNVYLFSISPLS
MT LTMCRNLITFLRETFLNRYVPFDAAVDFHRLIASTAIVLTVLHSVGHTTNTYQYSTSPQS
1141 1200
WT VLSCLFPGLFHDDGSELPQKYYWWFFQTVPGLTGVVLLLILAIMYVFASHHFRRRSFRGF
MT TQSCQYPGQYHDDGSELPQKYYWWFFQTTPGQTGTTQQQTQATMYTYASHHFRRRSFRGF
1201 1260
WT WLTHHLYILLYVLLIIHGSFALIQLPRFHIFFLVPAIIYGGDKLVSLSRKKVEISVVKAE
MT WLTHHQYTQQYTQQTTHGSYAQTQQPRYHTYYQTPATTYGGDKQTSQSRKKVEISVVKAE
1261 1320
WT LLPSGVTHLRFQRPQGFEYKSGQWVRIACLALGTTEYHPFTLTSAPHEDTLSLHIRAAGP
MT LLPSGVTHLRFQRPQGFEYKSGQWVRIACLALGTTEYHPFTLTSAPHEDTLSLHIRAAGP
1321 1380
WT WTTRLREIYSAPTGDRCARYPKLYLDGPFGEGHQEWHKFEVSVLVGGGIGVTPFASILKD
MT WTTRLREIYSAPTGDRCARYPKLYLDGPFGEGHQEWHKFEVSVLVGGGIGVTPFASILKD
1381 1440
WT LVFKSSVSCQVFCKKIYFIWVTRTQRQFEWLADIIREVEENDHQDLVSVHIYITQLAEKF
MT LVFKSSVSCQVFCKKIYFIWVTRTQRQFEWLADIIREVEENDHQDLVSVHIYITQLAEKF
1441 1500
WT DLRTTMLYICERHFQKVLNRSLFTGLRSITHFGRPPFEPFFNSLQEVHPQVRKIGVFSCG
MT DLRTTMLYICERHFQKVLNRSLFTGLRSITHFGRPPFEPFFNSLQEVHPQVRKIGVFSCG
1501 1560
WT PPGMTKNVEKACQLINRQDRTHFSHHYENF
MT PPGMTKNVEKACQLINRQDRTHFSHHYENF

g) DUOXA1 vs DUOXA1^{QTY}

Name	pI	MW (kDa)	Variation Rate (%)	Variation Rate (TM, %)
DUOXA1	6.30	37.82	/	/
DUOXA1^{QTY}	6.30	38.25	16.03	52.38

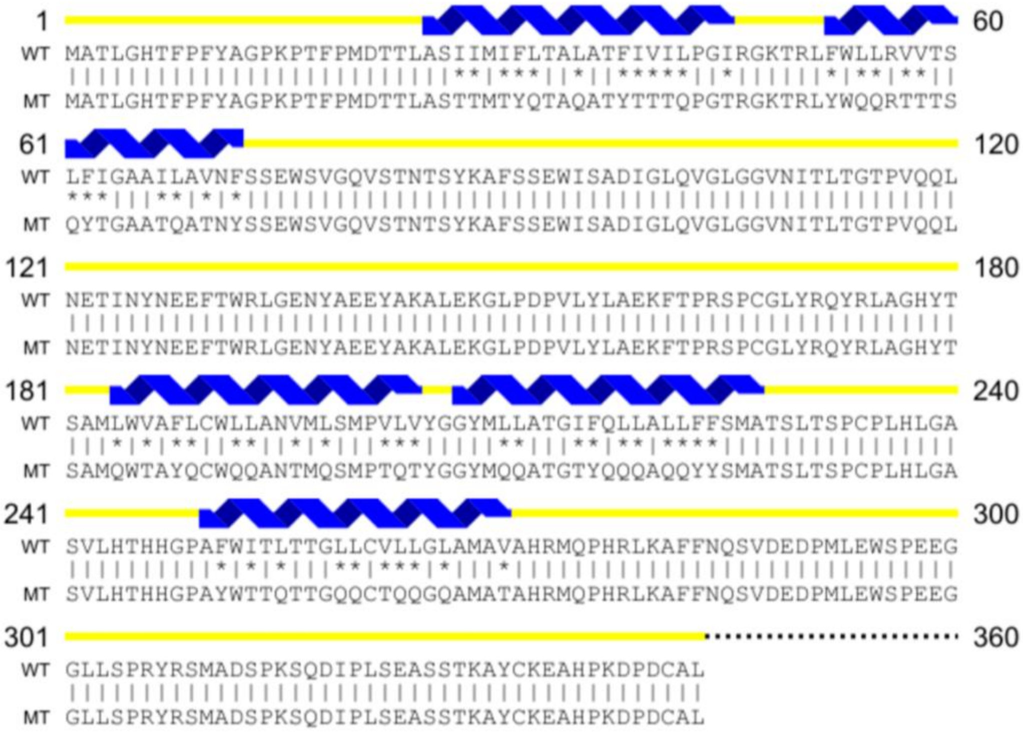

h) CYBA vs CYBA^{QTY}

Name	pI	MW (kDa)	Variation Rate (%)	Variation Rate (TM, %)
CYBA	9.58	20.88	/	/
CYBA^{QTY}	9.49	21.07	16.49	40.00

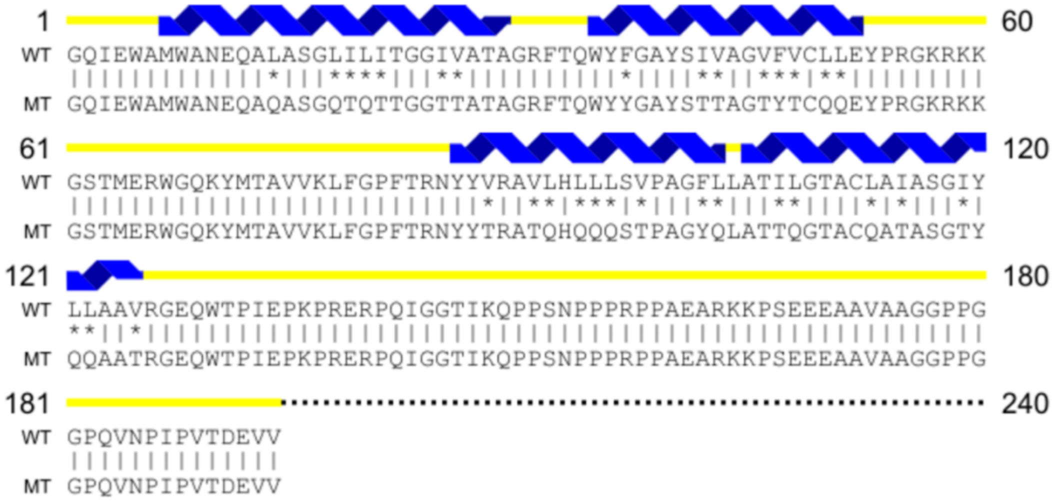

Figure S1. **The enlarged protein sequence alignments of eight native NADPH oxidases with their water-soluble QTY variants from Figure 1.** The symbols | and * indicate whether amino acids are identical or different, respectively. Q replaces L, T replaces V and I, and Y replaces F. The alpha helices (colored in blue) are shown above the protein sequences. The characteristics of native and QTY variants listed are isoelectric focusing (pI), molecular weight (MW), total variation % and transmembrane variation %. The alignments are a) NOX1 vs NOX1^{QTY}, b) NOX2 vs NOX2^{QTY}, c) NOX3 vs NOX3^{QTY}, d) NOX4 vs NOX4^{QTY}, e) NOX5 vs NOX5^{QTY}, f) DUOX1 vs DUOX1^{QTY}, g) DUOXA1 vs DUOXA1^{QTY}, h) CYBA vs CYBA^{QTY}. Compared to the native, the QTY variants show significant changes, ranging from 40.00% to 52.38%, in the TM region without significant changes in pI and MW.

A) NOX1$^{AlphaFold\ 3}$

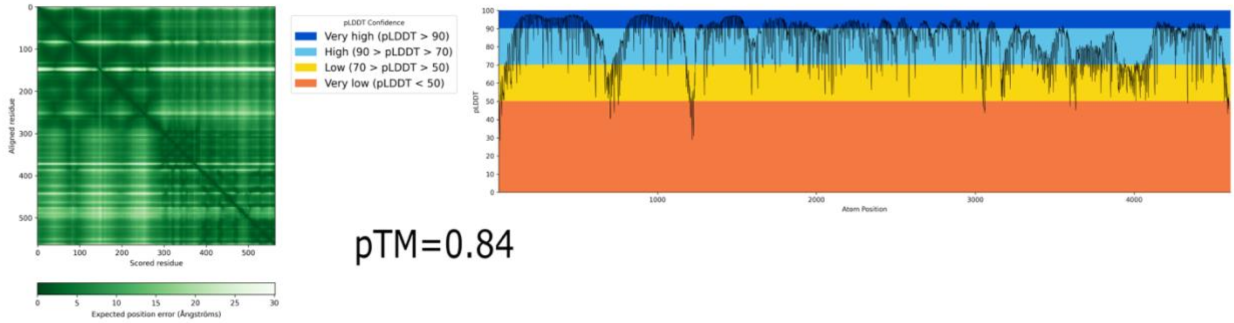

B) NOX2$^{AlphaFold\ 3}$

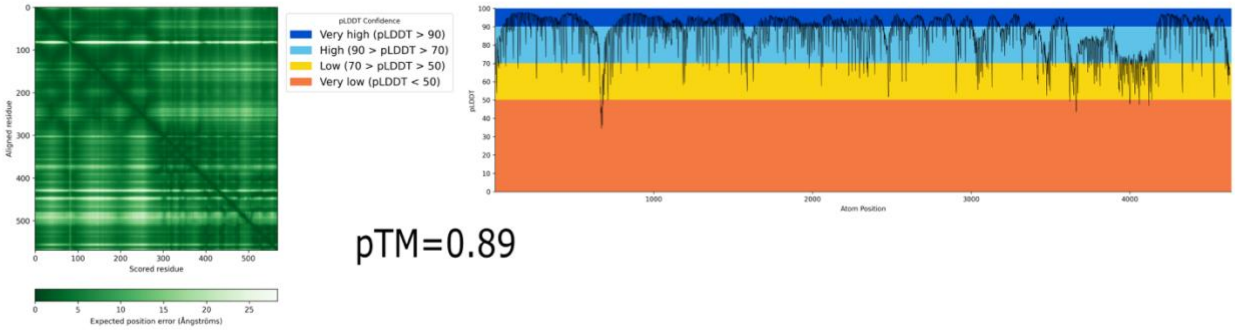

C) NOX3$^{AlphaFold\ 3}$

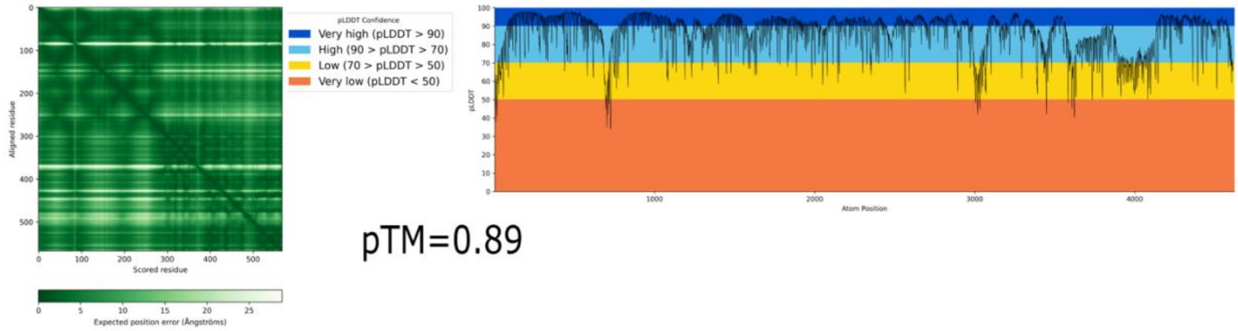

D) NOX4$^{AlphaFold\ 3}$

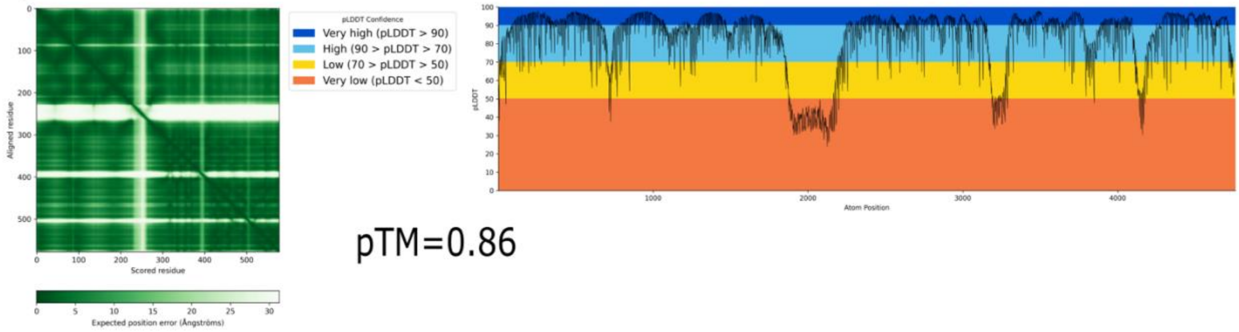

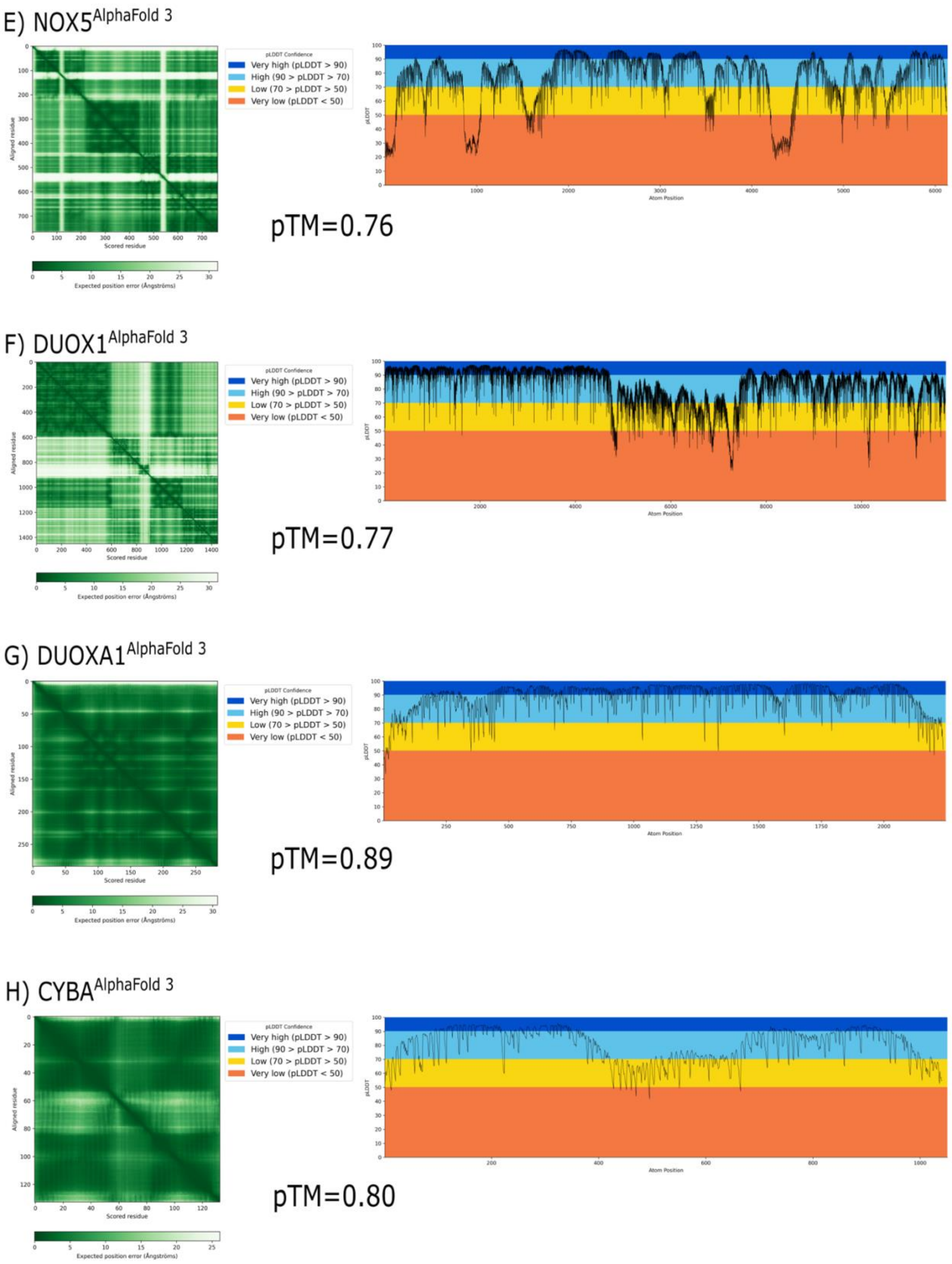

Figure S2. Enlarged panels from Figure 2. AlphaFold 3 predicted QTY variants with reduced hydrophobicity and pLDDT interval. pLDDT confidence profiles, PAE matrices, and pTM scores were generated for eight human transmembrane proteins. Predicted aligned error (PAE) matrices show predominantly low error (dark green), indicating high confidence in residue positioning, with

few regions of increased error. Transmembrane regions were predicted with predominantly high to very high confidence (blue and dark blue shaded regions), while some regions show lower confidence (yellow to orange). In contrast to the others, NOX5 and DUOX1 display extended regions of reduced confidence, indicating increased structural uncertainty. Overall high pTM scores indicate high prediction accuracy and strong resemblance to known experimentally determined protein structures. A) NOX1$^{\text{AlphaFold 3}}$, B) NOX2$^{\text{AlphaFold 3}}$, C) NOX3$^{\text{AlphaFold 3}}$, D) NOX4$^{\text{AlphaFold 3}}$, E) NOX5$^{\text{AlphaFold 3}}$, F) DUOX1$^{\text{AlphaFold 3}}$, G) DUOXA1$^{\text{AlphaFold 3}}$, H) CYBA$^{\text{AlphaFold 3}}$.

A) NOX1^{Native} vs NOX1^{QTY}

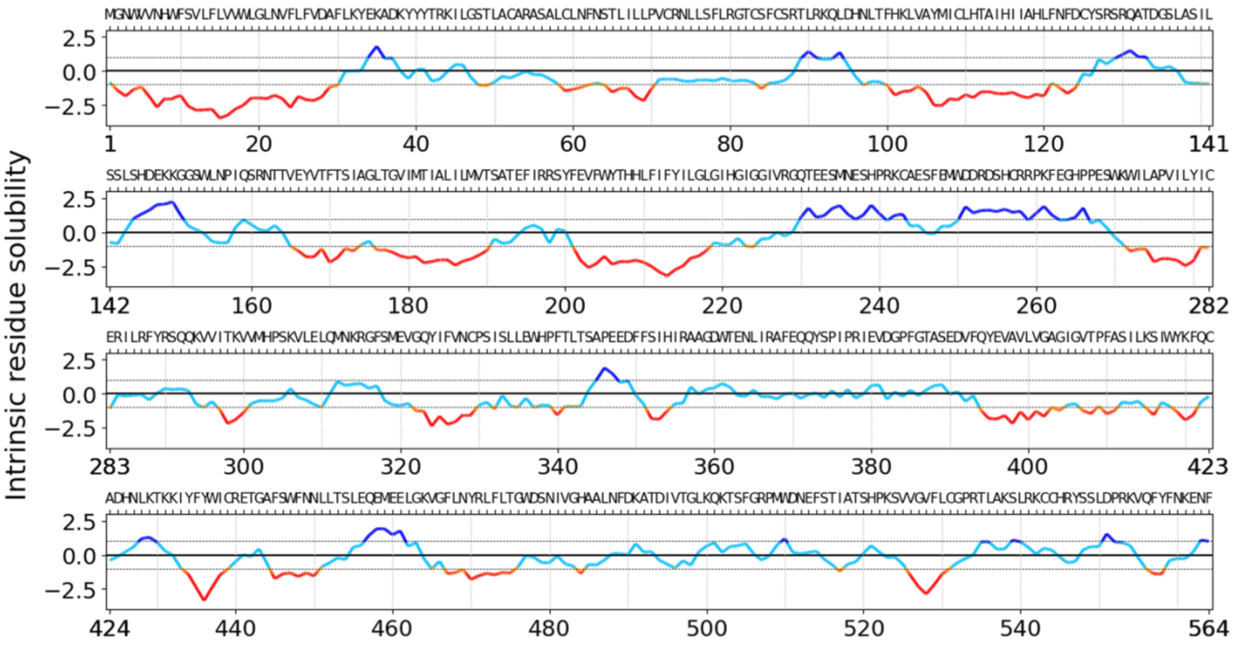

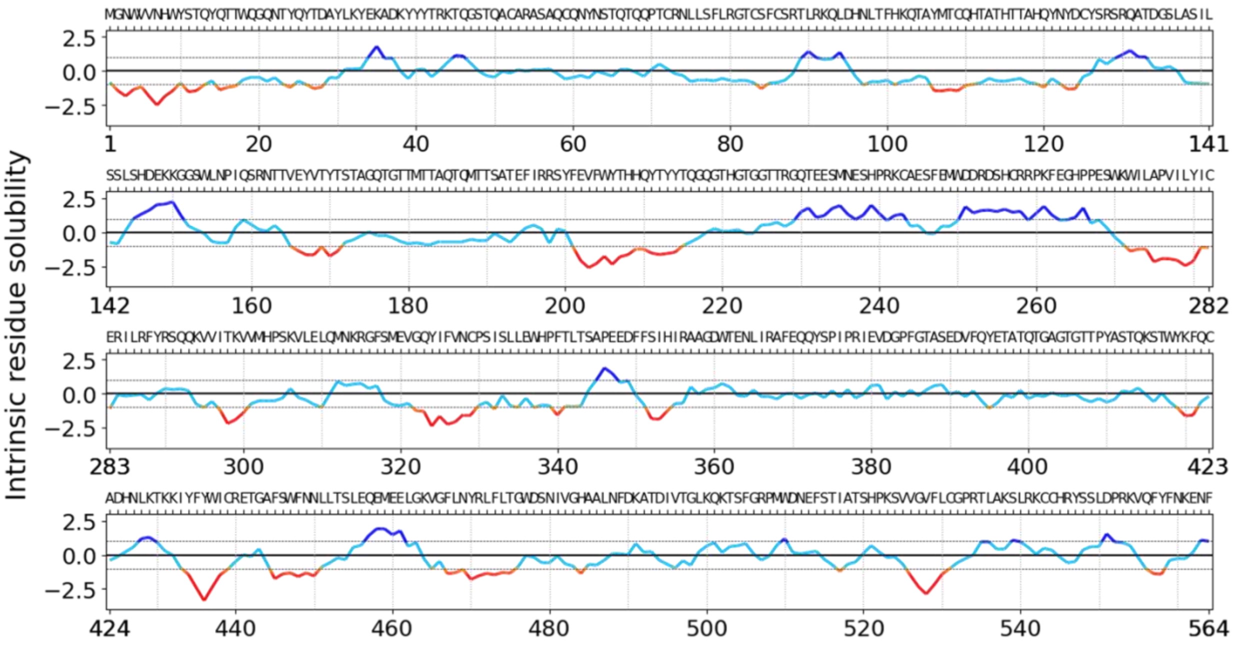

B) NOX2^{Native} vs NOX2^{QTY}

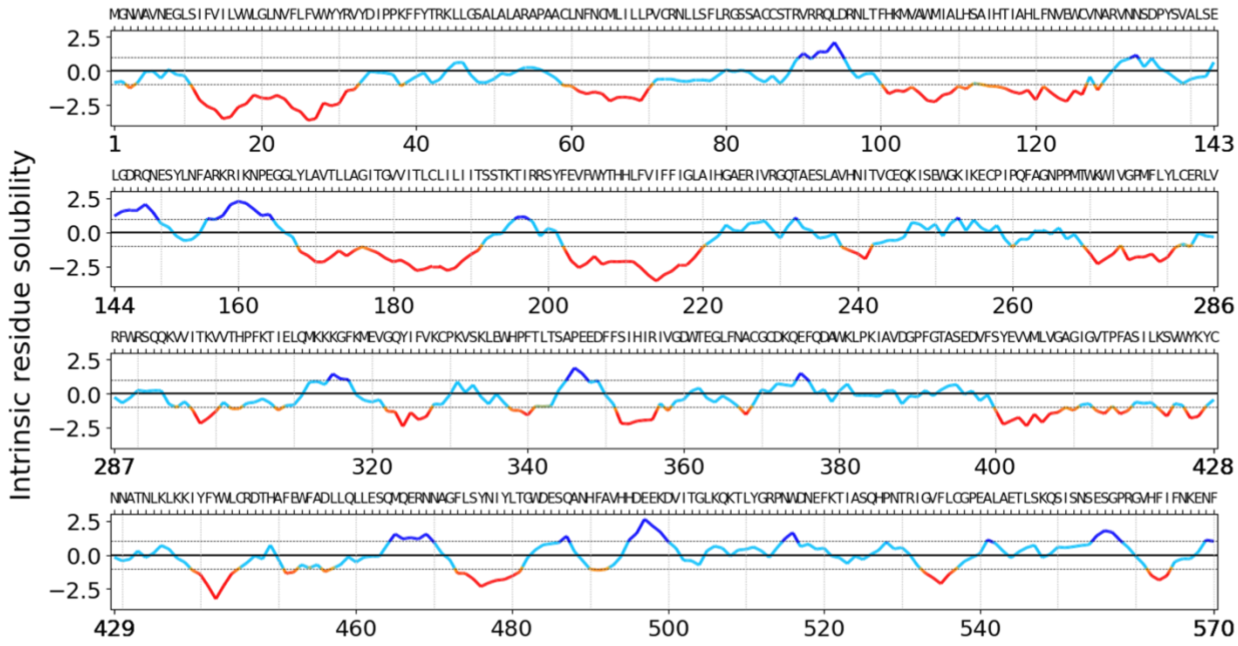

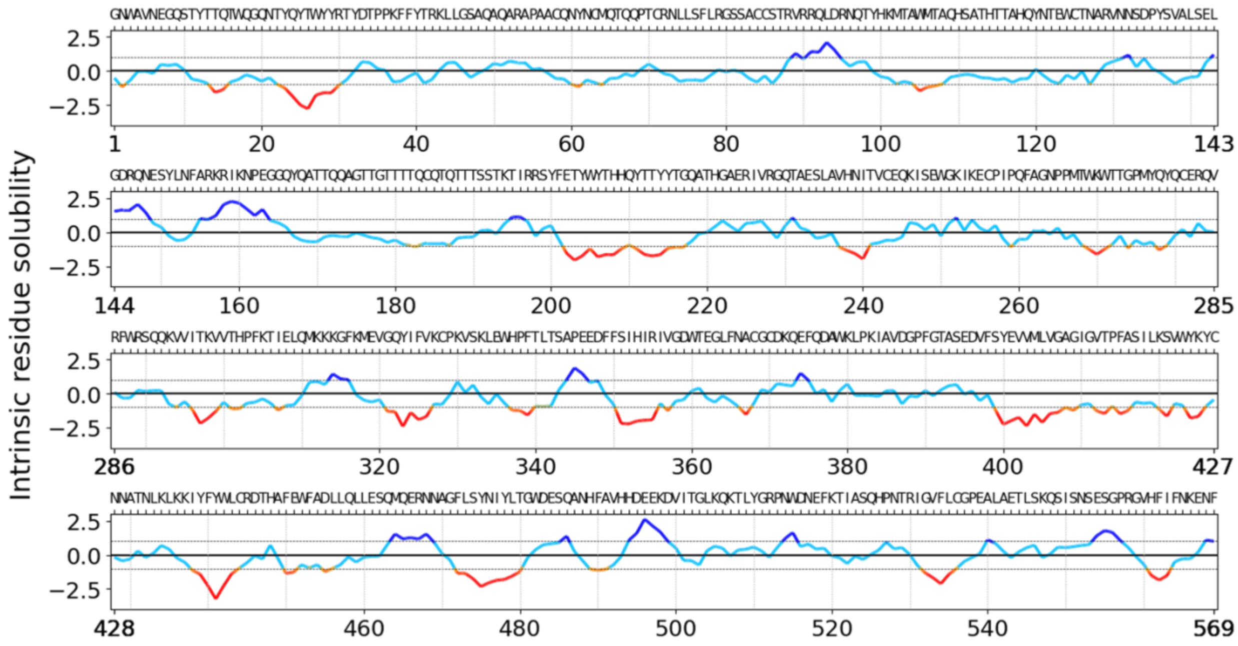

C) NOX3^{Native} vs NOX3^{QTY}

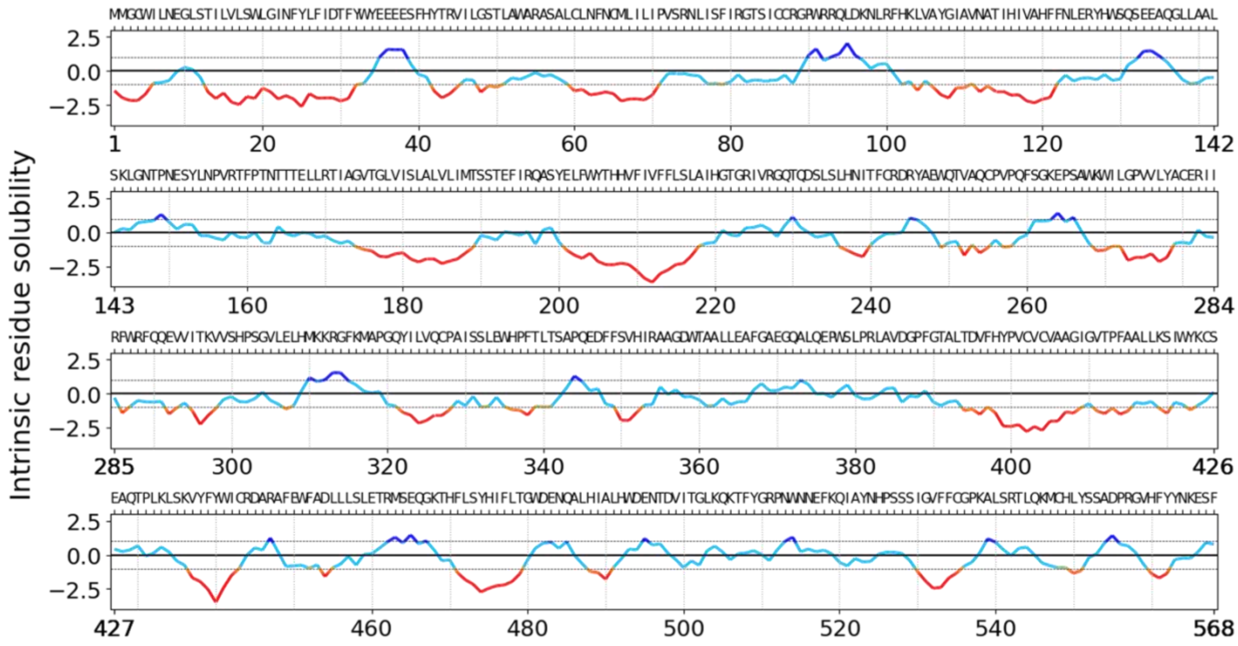

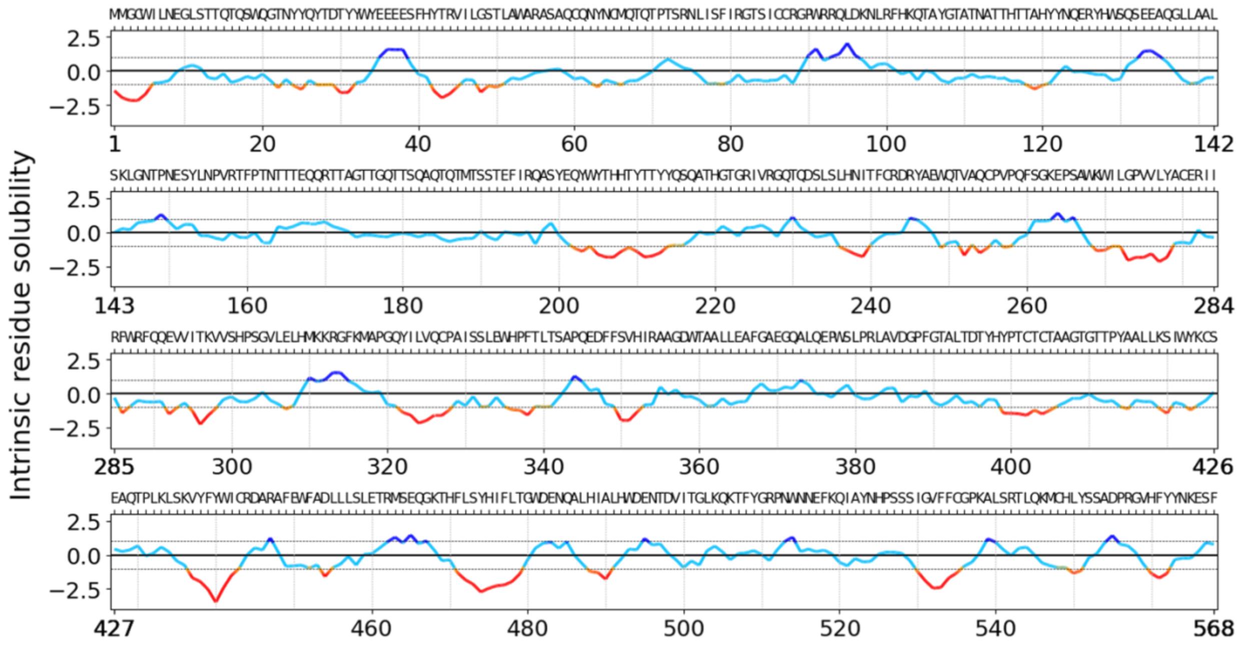

D) NOX4[Native] vs NOX4[QTY]

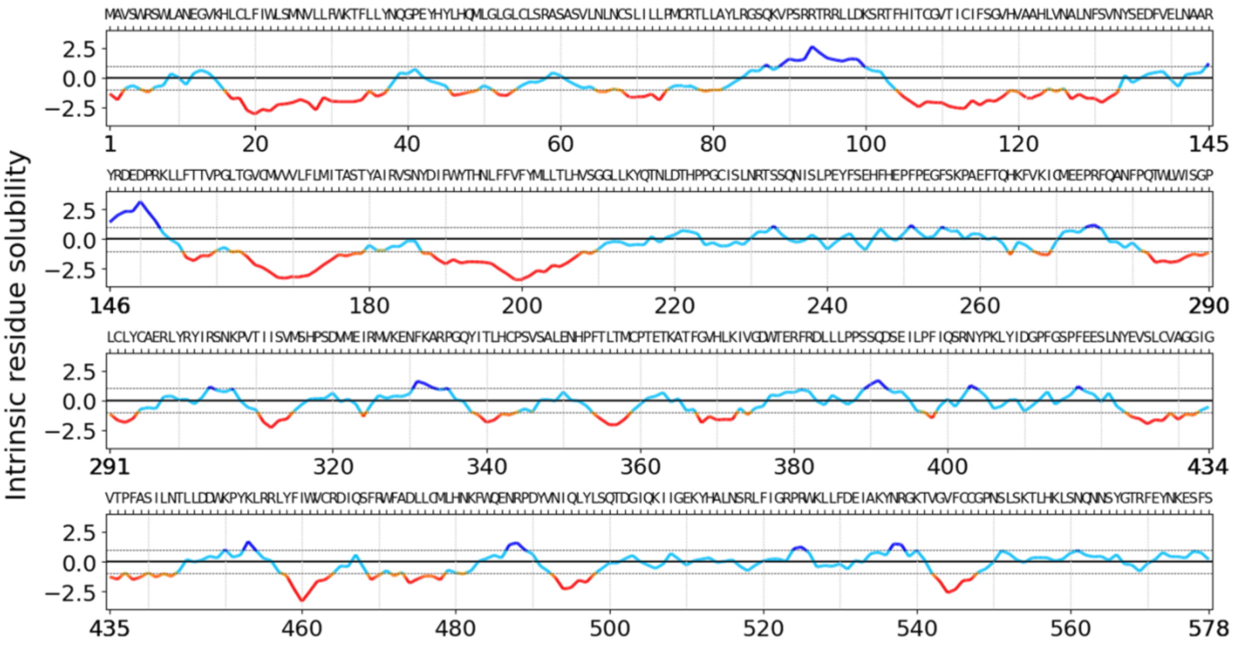

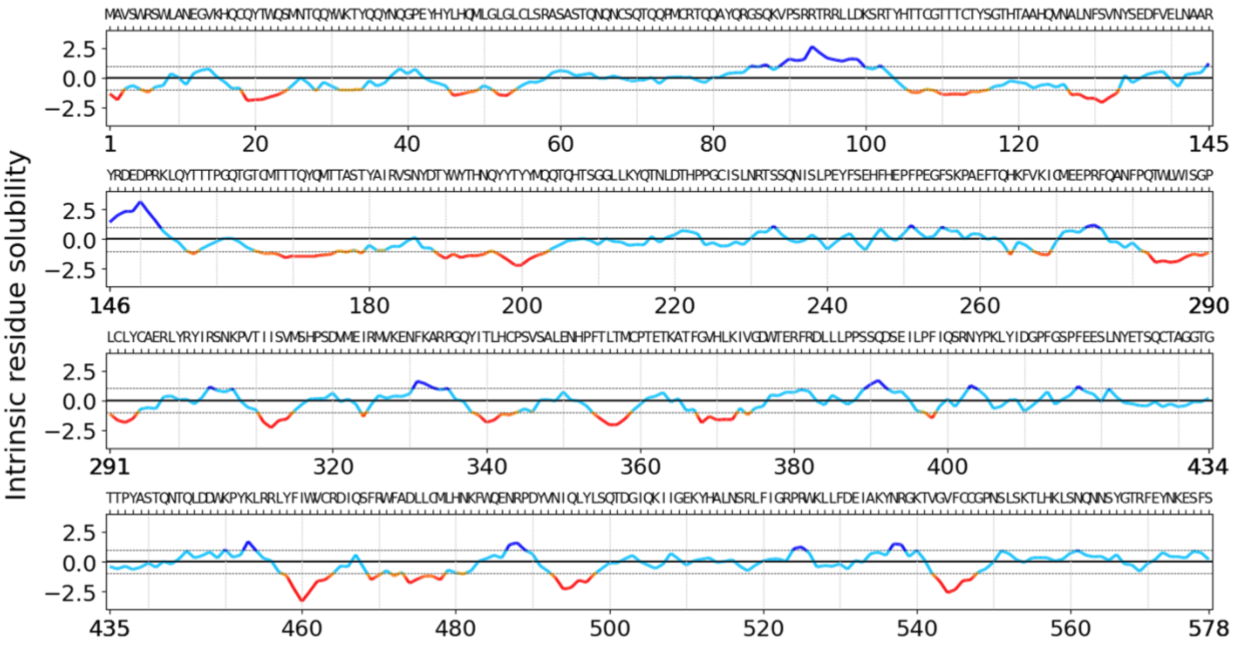

E) NOX5^{Native} vs NOX5^{QTY}

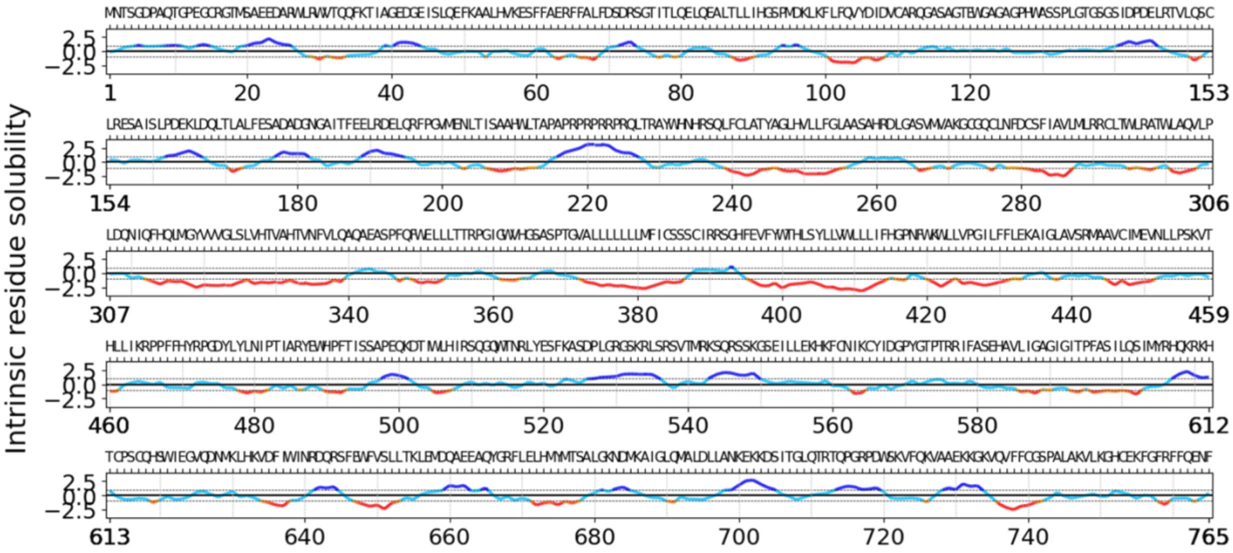

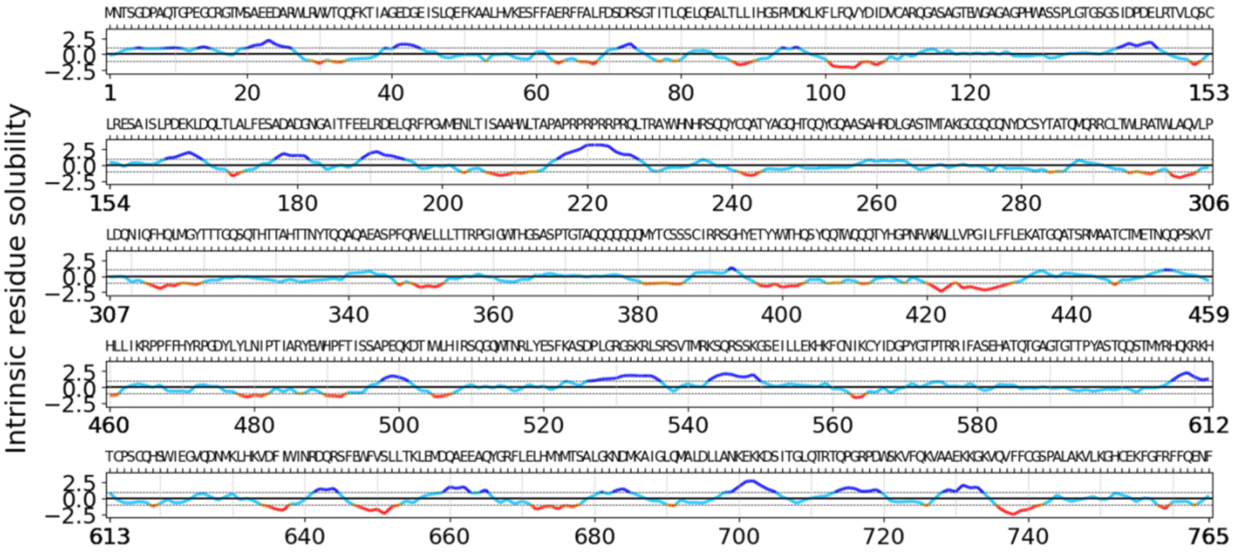

F) DUOX1[Native] vs DUOX1[QTY]

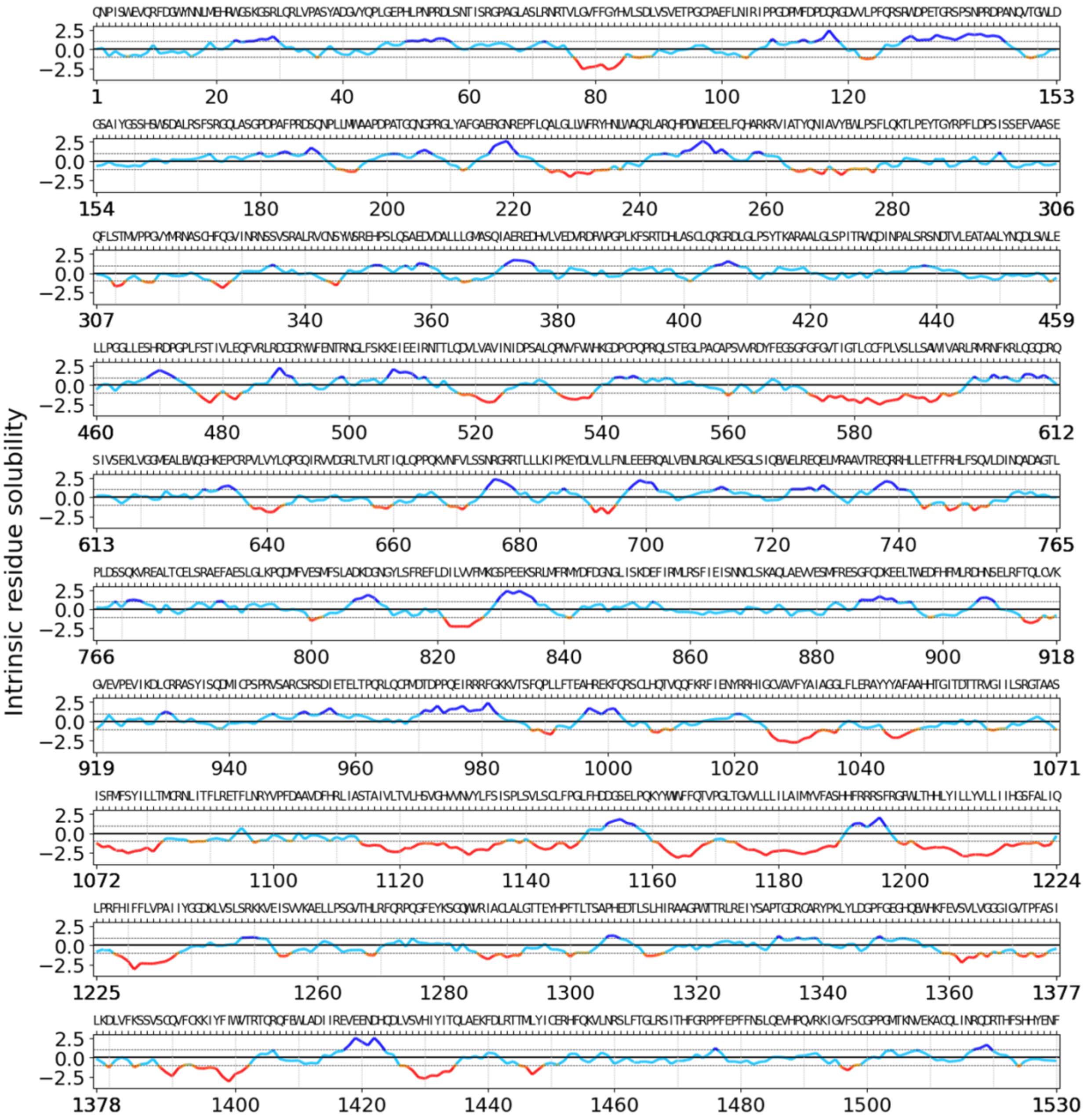

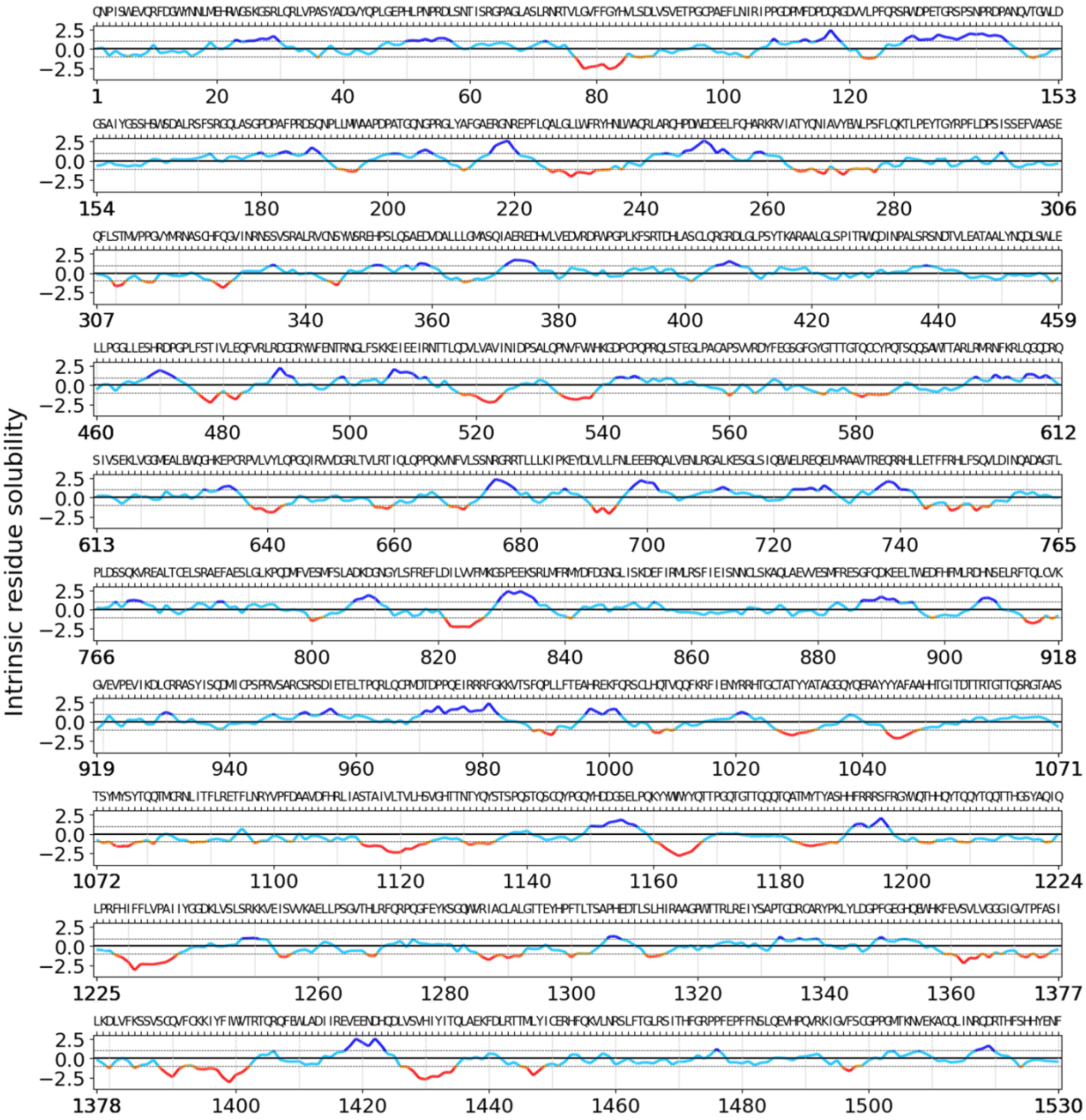
Intrinsic residue solubility

G) DUOXA1^{Native} vs DUOXA1^{QTY}

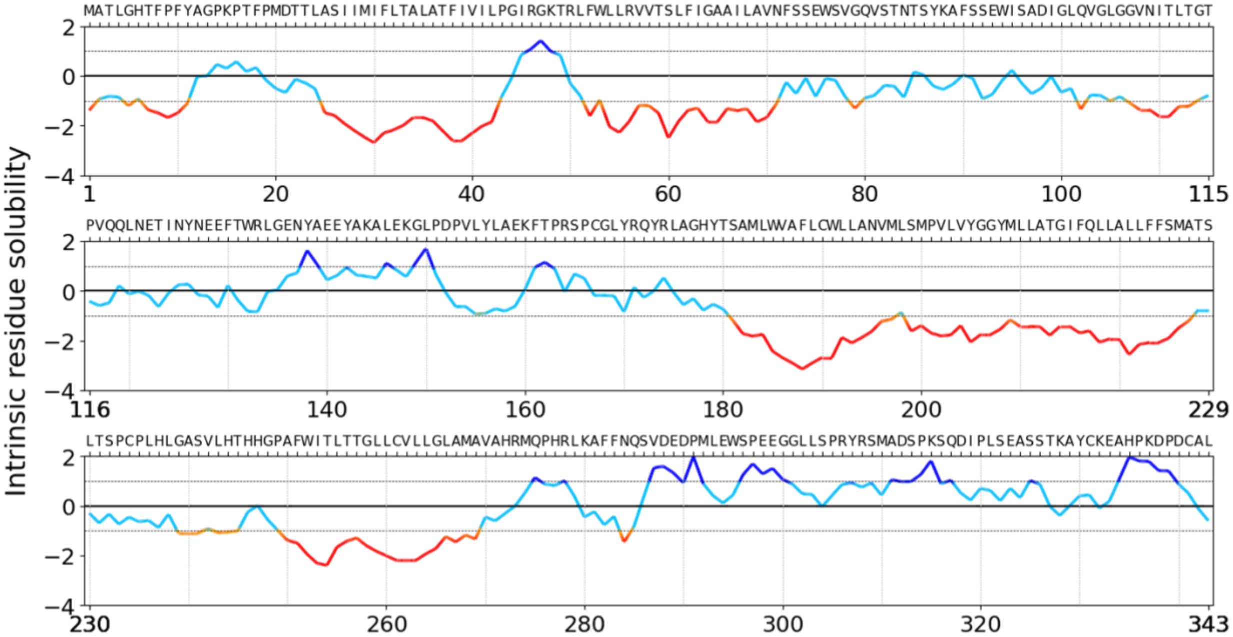

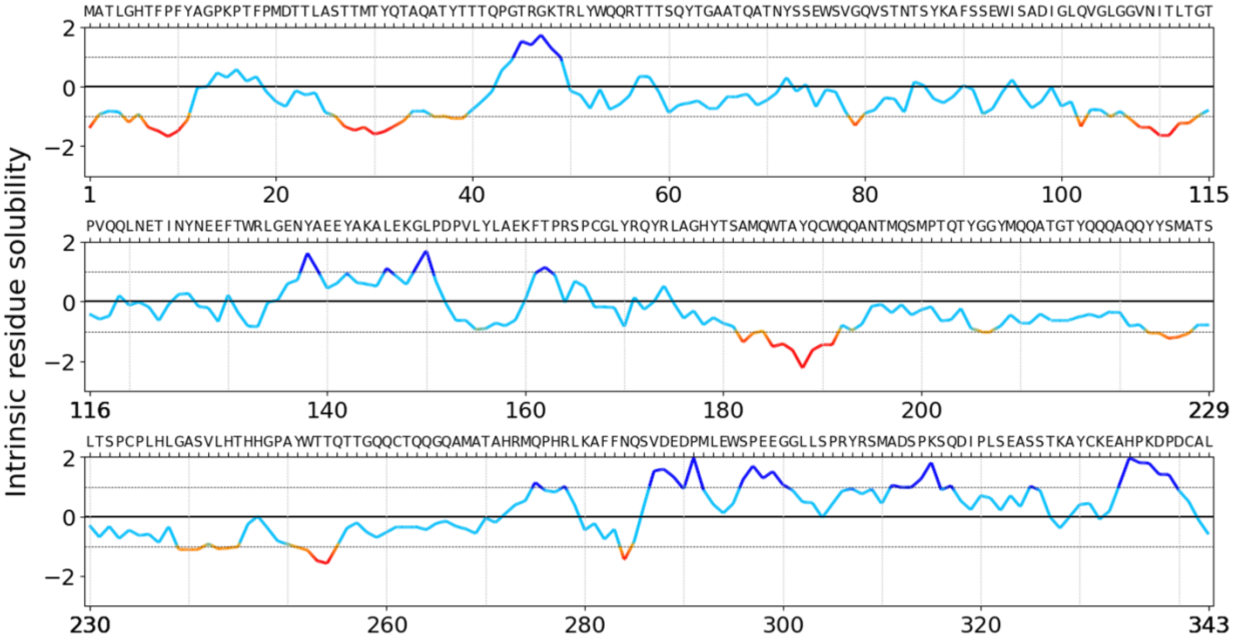

H) CYBA^{Native} vs CYBA^{QTY}

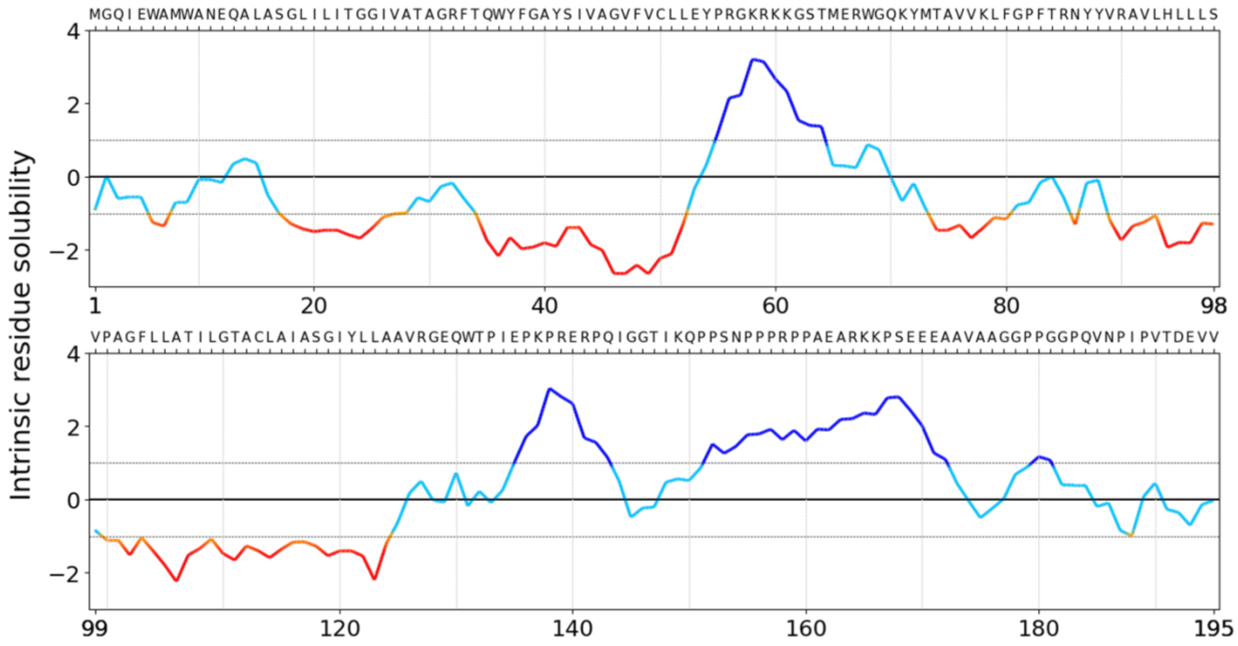

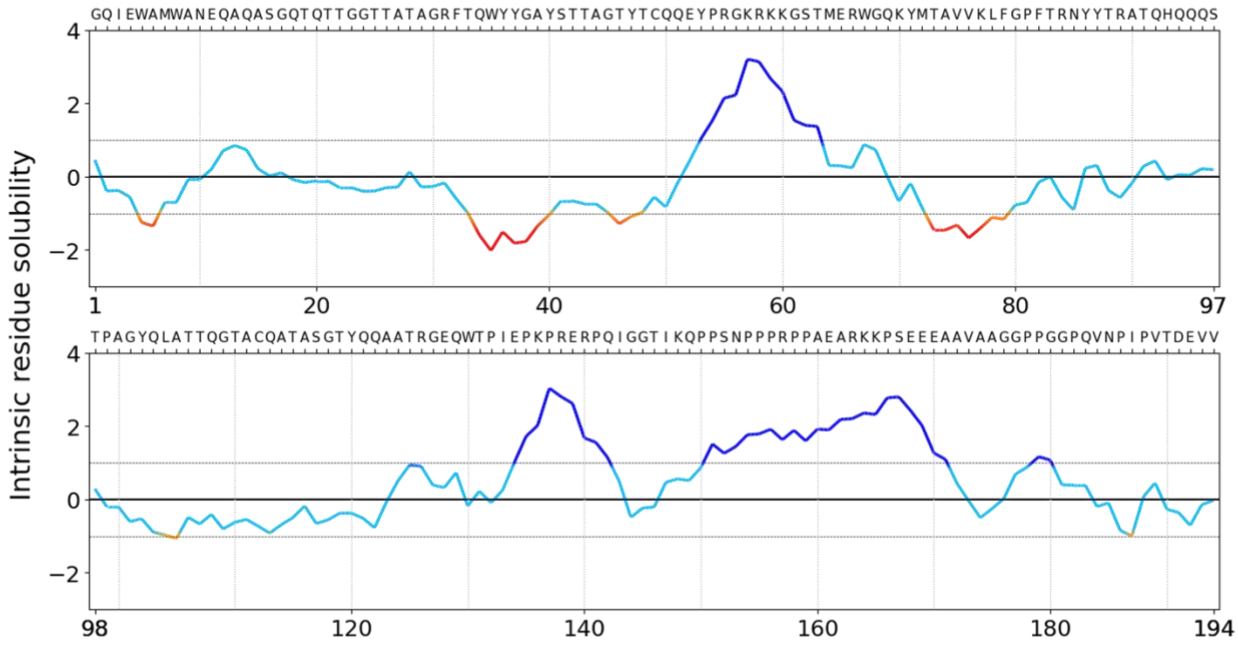

Figure S3. The enlarged individual panels of CamSol intrinsic profiles of eight NADPH oxidases and their QTY analogs with reduced hydrophobicity. The red regions represent highly insoluble aggregation-prone regions of proteins, while blue regions represent highly soluble and aggregation-resistant regions. All eight proteins showed reduced red and increased blue regions after application of the QTY code, suggesting reduced hydrophobicity. A) NOX1^{Native} vs NOX1^{QTY}, B) NOX2^{Native} vs NOX2^{QTY}, C) NOX3^{Native} vs NOX3^{QTY}, D) NOX4^{Native} vs NOX4^{QTY}, E) NOX5^{Native} vs NOX5^{QTY}, F) DUOX1^{Native} vs DUOX1^{QTY}, G) DUOXA1^{Native} vs DUOXA1^{QTY}, H) CYBA^{Native} vs CYBA^{QTY}.

a) NOX1

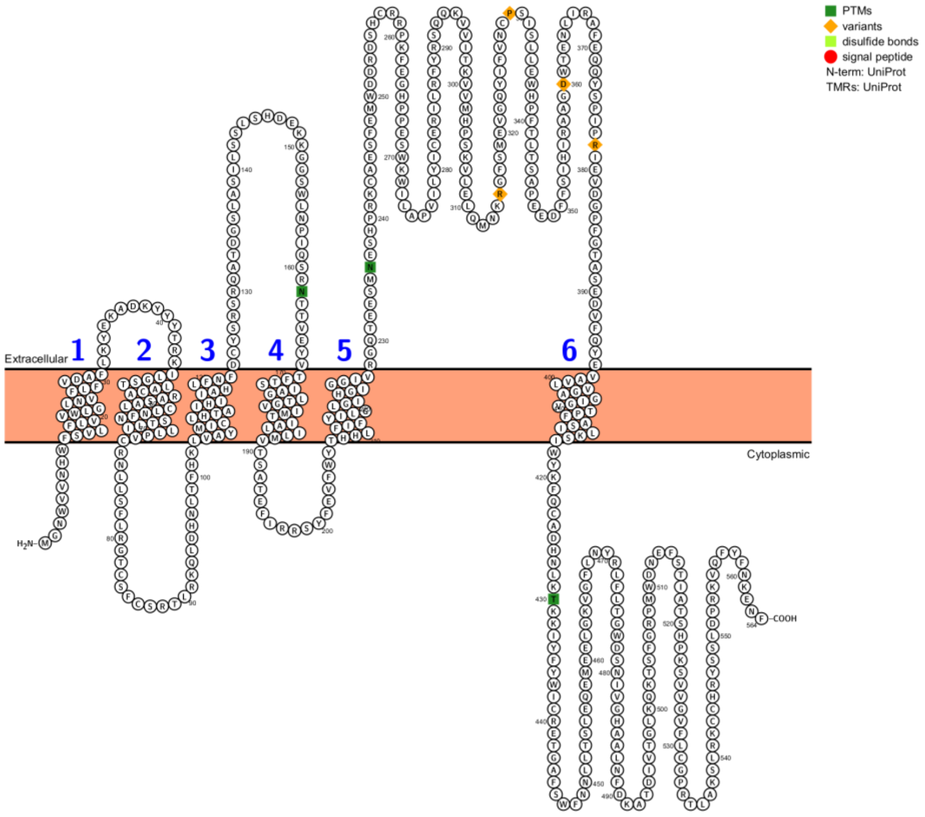
PTMs
variants
disulfide bonds
signal peptide
N-term: UniProt
TMRs: UniProt
Extracellular
Cytoplasmic
1
2
3
4
5
6

b) NOX2

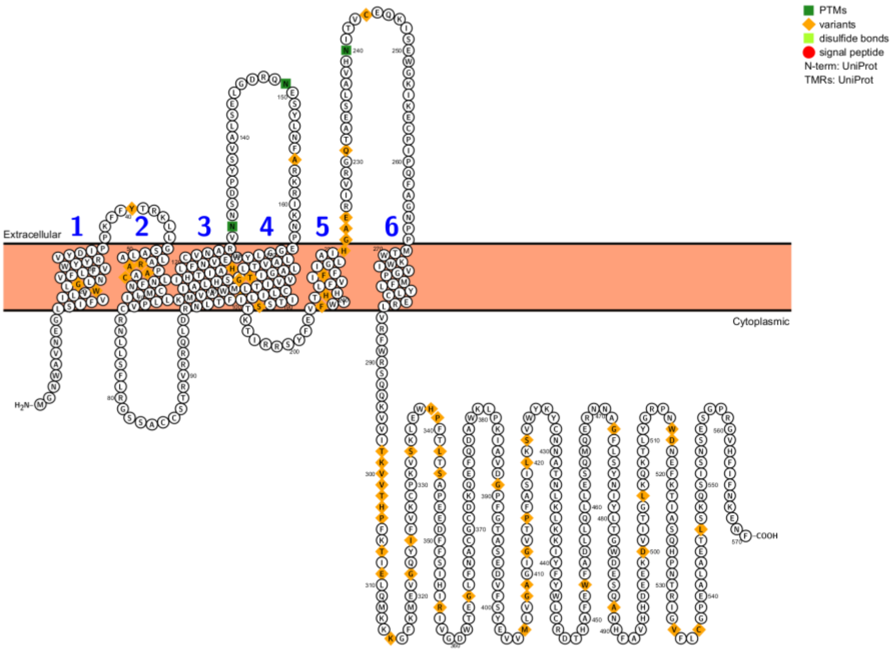
PTMs
variants
disulfide bonds
signal peptide
N-term: UniProt
TMRs: UniProt
Extracellular
Cytoplasmic
1
2
3
4
5
6
H2N-
-COOH

c) NOX3

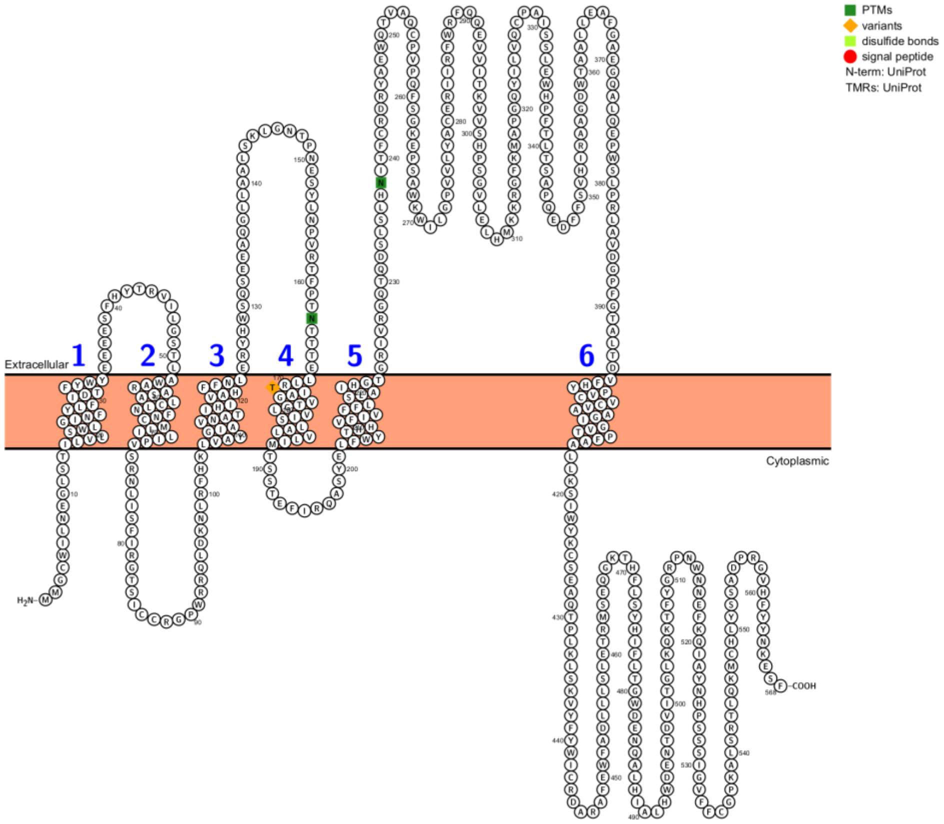
PTMs
variants
disulfide bonds
signal peptide
N-term: UniProt
TMRs: UniProt
Extracellular
Cytoplasmic
1
2
3
4
5
6
H2N-
-COOH

d) NOX4

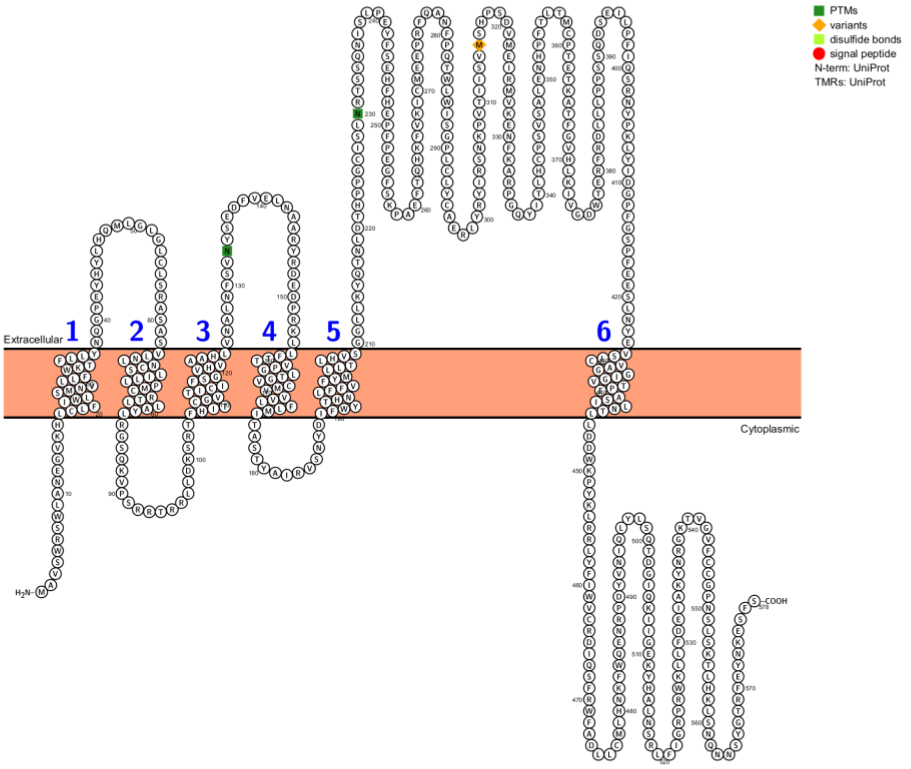
PTMs
variants
disulfide bonds
signal peptide
N-term: UniProt
TMRs: UniProt
Extracellular
1
2
3
4
5
6
Cytoplasmic
H2N-M
S-COOH

e) NOX5

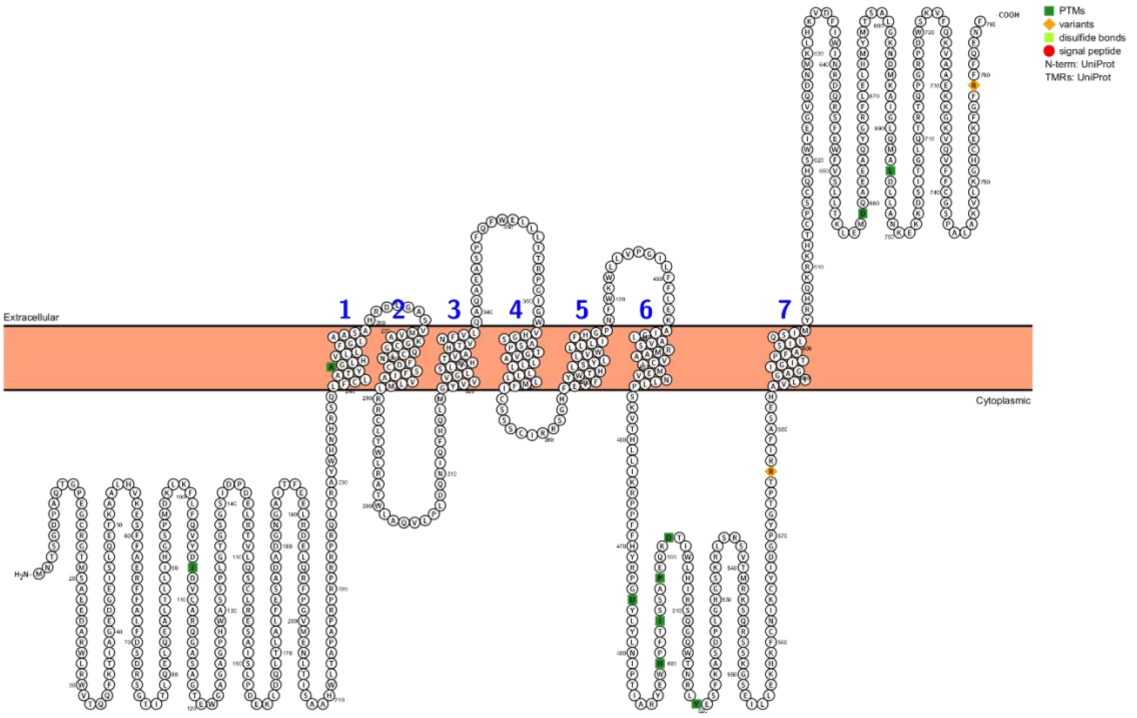
PTMs
variants
disulfide bonds
signal peptide
N-term: UniProt
TMRs: UniProt
Extracellular
1
2
3
4
5
6
7
Cytoplasmic
H2N-M
COOH

f) DUOX1

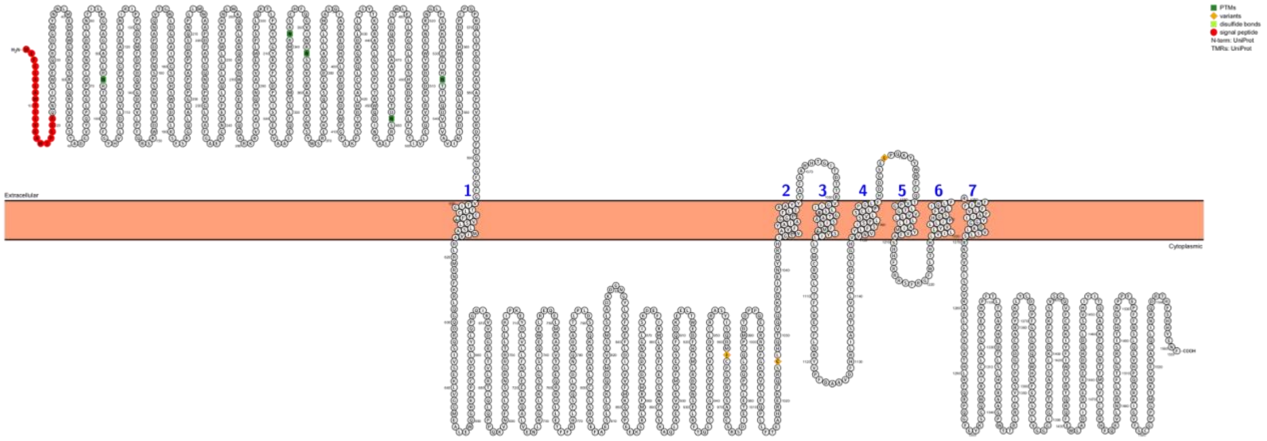

g) DUOXA1

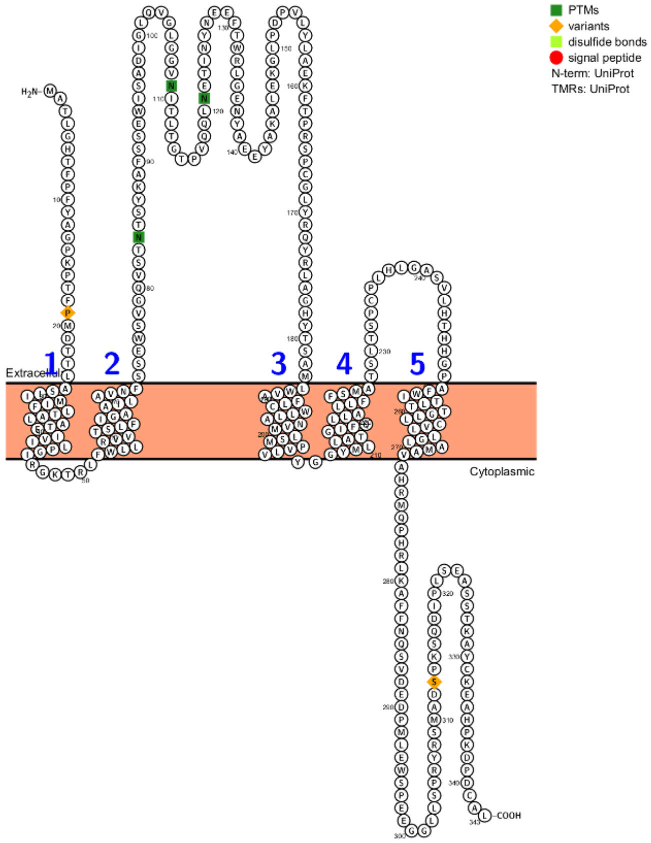

h) CYBA

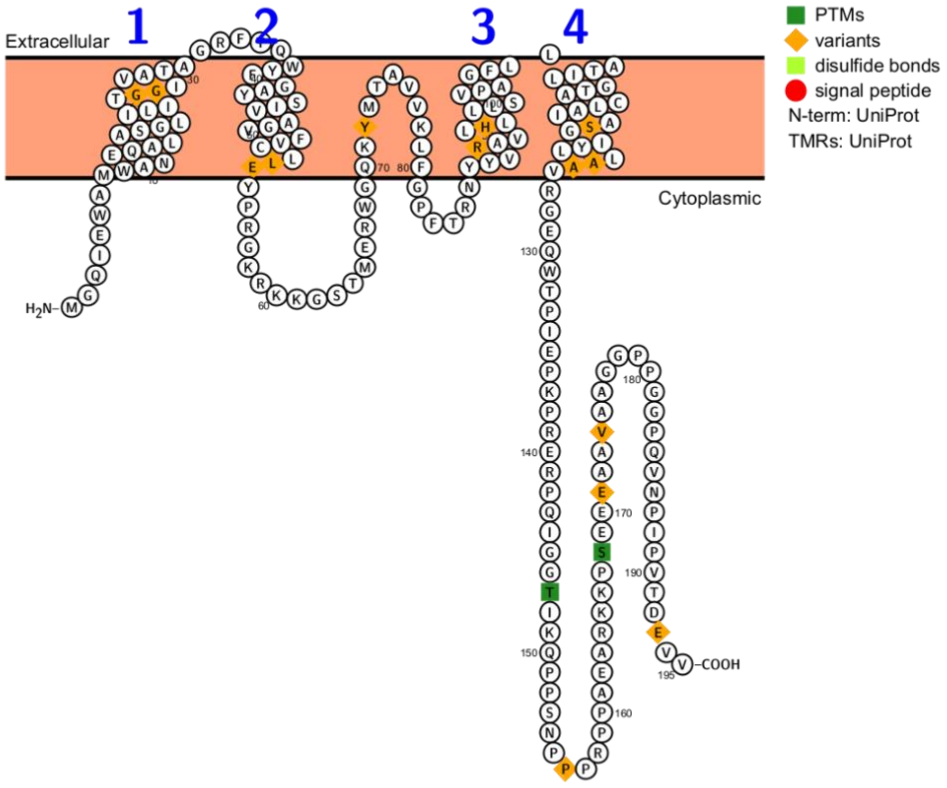

Figure S4. Membrane topology of eight NADPH oxidases. Topological structures were generated via Protter. The cell membrane is colored orange boarded by black lines. Topologies include: a) NOX1, b) NOX2, c) NOX3, d) NOX4, e) NOX5, f) DUOX1, g) DUOXA1, h) CYBA.

a) NOX1^{Native} vs NOX1^{QTY}

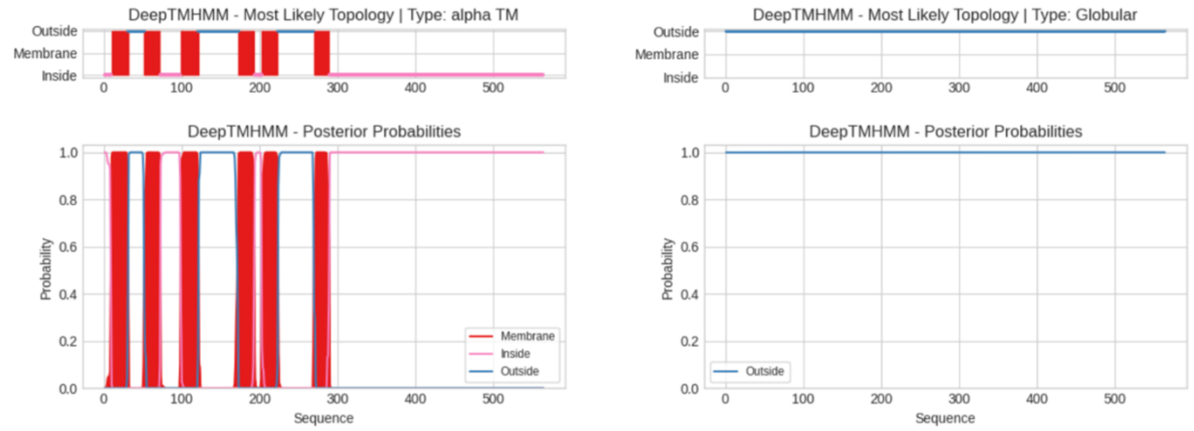

b) NOX2^{Native} vs NOX2^{QTY}

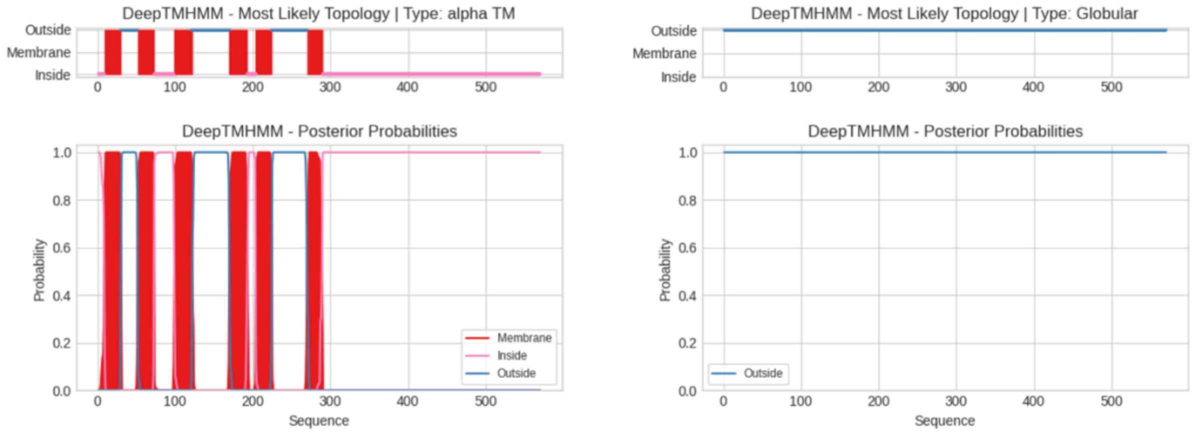

c) NOX3^{Native} vs NOX3^{QTY}

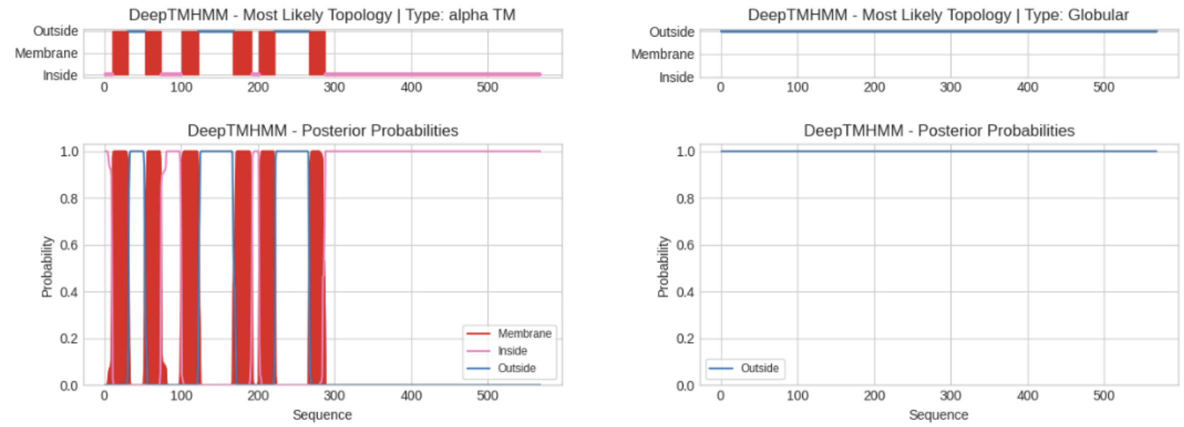

d) NOX4^{Native} vs NOX4^{QTY}

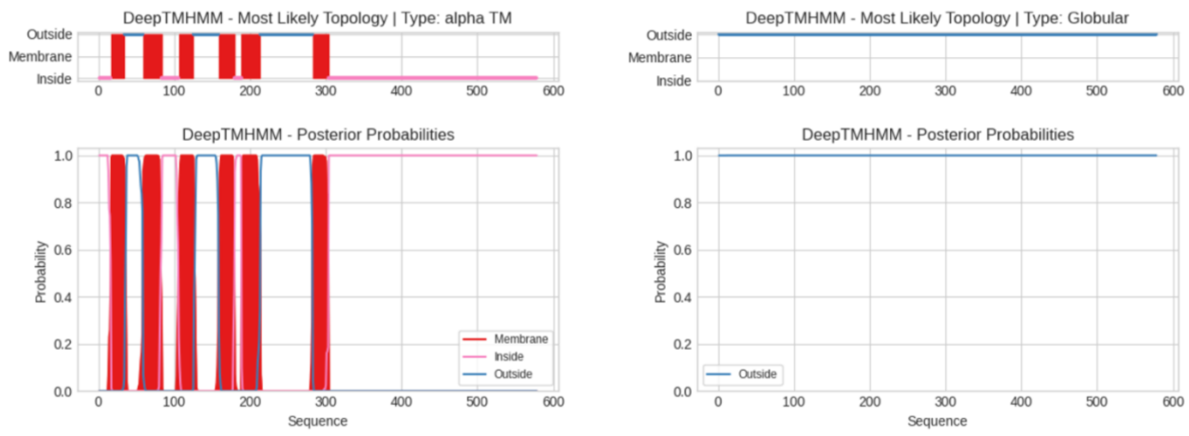

e) NOX5^{Native} vs NOX5^{QTY}

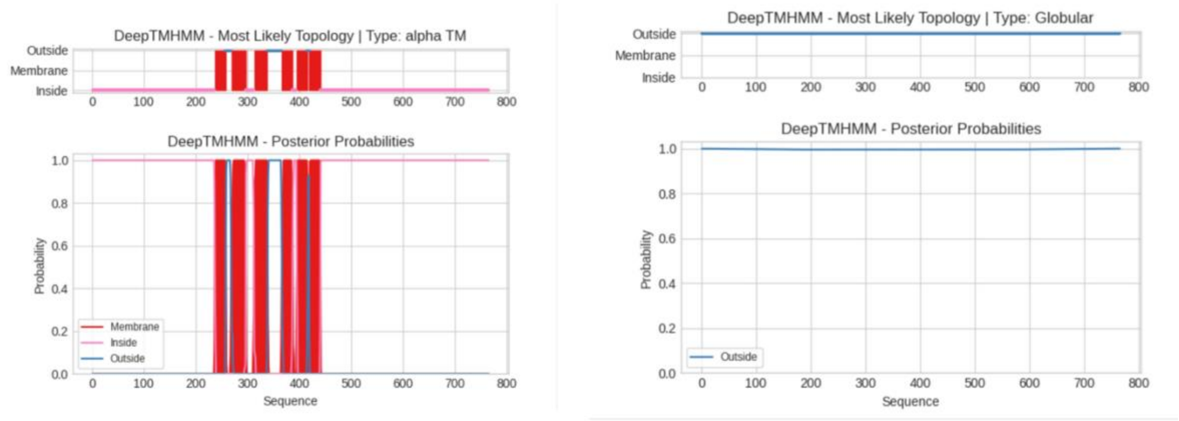

f) DUOX1^{Native} vs DUOX1^{QTY}

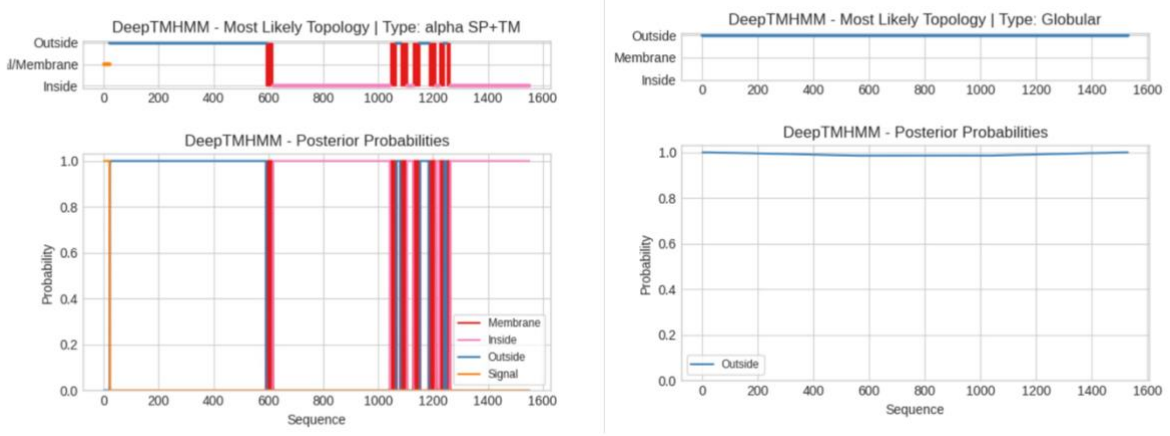

g) DUOXA1^{Native} vs DUOXA1^{QTY}

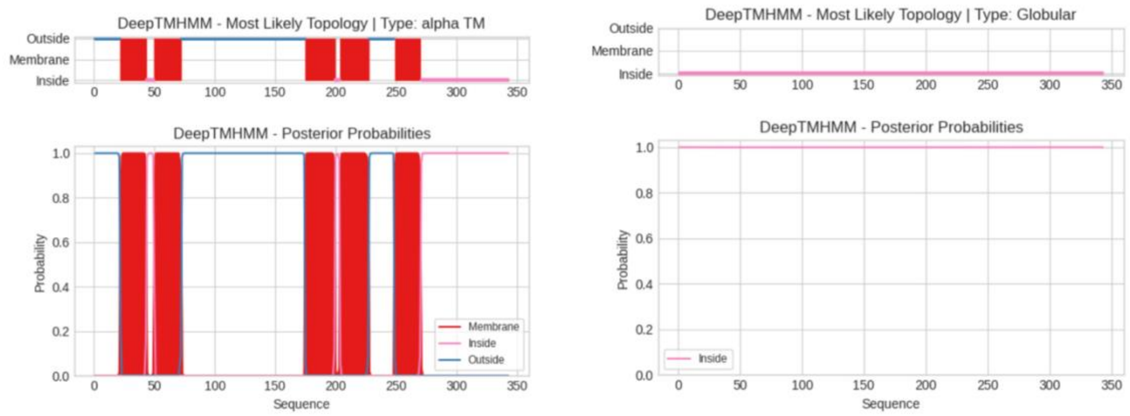

h) CYBA^{Native} vs CYBA^{QTY}

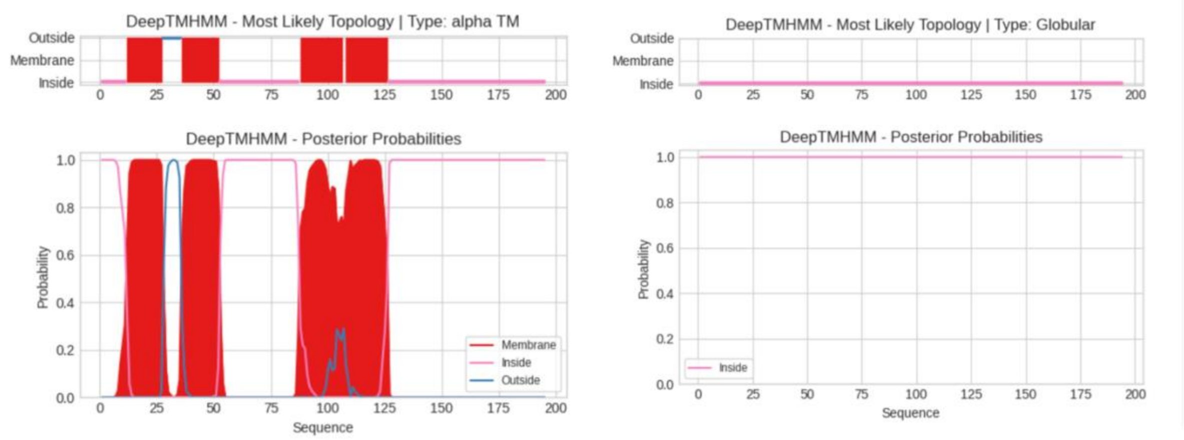

Figure S5. Transmembrane helix predictions of native and QTY analogs. Predictions were generated using DeepTMHMM. Predictions include a) NOX1^{Native} vs NOX1^{QTY}, b) NOX2^{Native} vs NOX2^{QTY}, c) NOX3^{Native} vs NOX3^{QTY}, d) NOX4^{Native} vs NOX4^{QTY}, e) NOX5^{Native} vs NOX5^{QTY}, f) DUOX1^{Native} vs DUOX1^{QTY}, g) DUOXA1^{Native} vs DUOXA1^{QTY}, h) CYBA^{Native} vs CYBA^{QTY}.
