# Supplementary material for: Structural bioinformatic studies of eight integral transmembrane NADPH oxidases and their AlphaFold 3 predicted QTY analogs with reduced hydrophobicity
Source: PLoS One. 2026 Jun 10;21(6):e0347525. doi: 10.1371/journal.pone.0347525 (PMC13252790; doi:10.1371/journal.pone.0347525)
Supplement: Attachment — Submitted filename: Response to Reviewers.docx [file pone.0347525.s003.docx]

Major issues

1. The manuscript confuses PDB codes with UniProt accessions. For example, “NOX2 (PDB: P04839)” lists a UniProt ID, not a PDB code. Please correct throughout (PDB IDs are four-character codes).

**Thank you for the feedback. We have corrected all the PDB IDs.**

2. The text uses “DOXA1”. Should this be “DUOXA1”? And CY24A should be CYBA? Please verify and update consistently.

**Thank you for the feedback. We have corrected DOXA1 to DUOX1, and CY24A to CYBA. DOXA1 is an abbreviation of DUOXA1. CY24A is the antibody identifier, while CYBA is the gene/protein name.**

3. Consider moving “The rationale of the QTY code” to the Methods section for better flow.

**Thank you for the feedback. We have moved “The rationale of the QTY code” to the methods section.**

4. The figures appear low-resolution. Please ensure they meet the journal’s figure specifications (size, dpi, font legibility).

**Thank you for the feedback. We have updated figures with higher resolutions.**

5. Please specify the AlphaFold 3 model version/date, MSA and template settings, complex vs monomer mode, number of recycles, random seeds, and any restraints. Provide raw AF3 outputs (e.g., JSONs, PDBs, logs) to support reproducibility.

**Thank you for the feedback. We have added additional AF3 simulation information. We have specified the AlphaFold 3 parameters (monomer mode, version, MSA and template setting, seeds) in the “AlphaFold 3” section in Methods. Since we only have one protein sequence per entry, we are by default in monomer mode. There are no other user-end input changes, and all other settings are by default.**

6. Without experimental validation, the manuscript cannot conclude that the designed proteins are “water-soluble.” The analyses shown indicate reduced hydrophobicity, which is not equivalent to demonstrated water solubility. To maintain rigor, replace “water-soluble QTY analogs” with “QTY analogs with reduced hydrophobicity.”

**Thank you for the feedback. We have replaced “water-soluble QTY analogs” with “QTY analogs with reduced hydrophobicity” both in the title and in the main text.**

7. Beyond my point #6, please add quantitative evidence that the designs remain stable in water and show how much the hydrophobicity is reduced. To keep the work purely computational, the authors could perform a few-microsecond explicit-solvent MD simulations and/or use of protein solubility and thermostability prediction methods. This would further strengthen the claims.

**Thank you for the feedback. We have added additional quantitative hydrophobicity analysis using the CamSol tool (https://www-cohsoftware.ch.cam.ac.uk/index.php/camsolintrinsic). New section of “Hydrophobicity Analysis” explaining the rationale for the tool in the methods section and the new paragraph in the “Analysis of the hydrophobic surface of native NADPH oxidases and their QTY analogs with reduced hydrophobicity” section are added to address the CamSol tool. Intrinsic solubility scores are also included in Table 2 for comparison between hydrophobicity of native structures and their QTY analogs.**

Minor issues

There are several terminology errors that I noticed. The authors should proofread the manuscript more carefully.

1. pI should be “isoelectric point”, not “isoelectric focusing”.

**Thank you for the feedback. We have changed all “isoelectric focusing” to “isoelectric point”.**

2. RMSD is commonly used as “root-mean-square deviation”, not “residue mean square distance”, although the meaning is almost the same.

**Thank you for the feedback. We have updated RMSD’s full name.**

3. “kDA” should be “kDa”

**Thank you for the feedback. We have updated the manuscript accordingly.**

4. Mixed use of “AlphaFold 3” and “AlphaFold3”.

**Thank you for the feedback. We have updated all to be “AlphaFold 3”.**

5. Mixed use of “PyMOL” and “PyMol”.

**Thank you for the feedback. However, we were unable to find these instances.**

6. You alternate between AF3/AlphaFold 3. Also, sometimes say “AlphaFold predicted” without “3”. Please consider standardize and keep labels uniform.

**Thank you for the feedback. We have clarified that all mentions of “AlphaFold” refer to AlphaFold 3 and kept consistency by using AlphaFold 3 throughout.**

Also, some minor points.

1. References [4] and [31] are duplicated.

**Thank you for the feedback. We have deleted the duplicated reference and updated all footnote numbers.**

2. Use consistent decimal precision for RMSD values. I recommend two decimal points.

**Thank you for the feedback. We have changed all RMSD values to two decimal points.**

3. Once defined, use abbreviations consistently (e.g., pI, Q, T, Y) in subsequent text. For example, in “Notably, the protein isoelectric point (pI) remains roughly unchanged, as the substituted amino acids Q (glutamine), T(threonine), and Y (tyrosine)..”, pI, Q, T, Y have been defined above.

**Thank you for the feedback. We have deleted the abbreviation descriptions.**

4. AF3 predictions for NOX5 are low confidence. Consider treating this as a cautionary case study and discuss limitations explicitly.

**Thank you for the feedback. In the section titled “Superpositions of CryoEM structures with AlphaFold 3–predicted native structures and their QTY analogs with reduced hydrophobicity,” we have added a discussion explaining how the low confidence of NOX5’s AlphaFold 3 prediction likely contributes to the high RMSD observed between its native structure and the AlphaFold-predicted QTY analog. We present this as an important caution: although AlphaFold 3 can serve as a useful reference tool for evaluating the efficacy of the QTY code, its predictions are not perfect. Therefore, the elevated RMSD score may not necessarily indicate a failure of the QTY code but may instead reflect limitations in the AlphaFold 3 prediction itself.**

5. Remove subpanels labeled “No CryoEM structure available” and note the lack of experimental structures in the caption and/or main text instead.

**Thank you for the feedback. We have removed these panels and noted absence of CryoEM structures in captions of Figures 3 and 5.**
