# Supplementary material for: Structural bioinformatic studies of eight integral transmembrane NADPH oxidases and their AlphaFold 3 predicted QTY analogs with reduced hydrophobicity
Source: PLoS One. 2026 Jun 10;21(6):e0347525. doi: 10.1371/journal.pone.0347525 (PMC13252790; doi:10.1371/journal.pone.0347525)
Supplement: Attachment — Submitted filename: 3.26 - Revision.2 - Response to Reviewers.docx [file pone.0347525.s004.docx]

**Major issues**

1. After revision, the term “water-soluble” still appears in figure captions and the main text (e.g., “confirming exceptional structural conservation between native NADPH oxidases and their water-soluble QTY variants”). I am not convinced that the QTY designs are water-soluble without experimental evidence. Water-soluble should be framed explicitly as a hypothesis and the manuscript should be revised accordingly throughout.

- **Thank you for the feedback. We had previously noticed this problem and addressed it. However, it appears that we forgot to make this change across both copies of the document (Manuscript with Tracked Changes and Manuscript without Tracked Changes). We have corrected this issue so both copies are correct.**
- **Additionally, we have also softened the language throughout the text and cautioned the limitations of computation prediction methods without experimental validation.**

2. In the conclusion, the authors claim that “our findings reveal remarkable resemblance between QTY analogs and their native proteins, suggesting minimal functional impact despite significant amino acid substitution.” I am not convinced because the QTY structures are all AlphaFold 3 predictions, not experimental structures. On the other hand, even if the global fold remains similar, function could still be compromised if substitutions perturb key interactions or interfaces with binding partners etc. These statements should be substantially softened, and the limitations of using AF3 structure as a validation should be made more explicit.

- **Thank you for the feedback. We have substantially softened the language in the section and cautioned the limitations of computational prediction methods without experimental validation.**

3. Similarly, I think the statements like “may function as water-soluble reducing agents for controlling ROS levels…” is a far reach.

- **Thank you for the feedback. We have removed this statement from the text.**

4. Panel labels for Figures 3 and 5 should be updated, and figure citations in the captions and main text should be corrected for consistency.

- **Thank you for your feedback. We’ve updated the captions for Figure 3 and 5 accordingly.**

5. In Table 2, where cryo-EM structures are available, RMSDs for QTY analogs should be calculated against the experimental cryo-EM structures, not against AF3 structures. Only when experimental structures are unavailable should AF3 predictions be used, and those cases should be clearly marked as such.

- **Thank you for your feedback. It appears we had not labeled the table correctly. Our RMSD values had previously been calculated against CryoEM structures when available, and AlphaFold 3 predicted native structures when unavailable. We have clarified this in the table caption.**
- **Upon closer inspection, we also noticed some minor errors in the QTY sequences for some of the proteins. We have redone all relevant calculations to account to ensure accuracy.**

6. Related to point 5, I suggest the authors merge Figures 4 and 5 so that QTY structures are compared directly with cryo-EM structures when available. When cryo-EM structures are unavailable, comparison to AF3 predictions is acceptable, but must be clearly marked. Comparing QTY structures to AF3 structures in cases where cryo-EM structures exist is not informative.

- **Thank you for the feedback. We combined Figures 3 and 4 so that QTY structures were compared directly with CryoEM structures when available and compared to AlphaFold 3 predicted native structures when not available. We have also updated the figure caption accordingly.**
- **We have kept Figure 5 as it may serve as a useful comparison for the 3 different superposed structures, when available (AF3 predicted QTY, AF3 predicted native, CryoEM native.**

**Minor issues**

1. I do not recommend using screenshots as figures. The same information can be presented using the AlphaFold server output data and generate figures by the authors. For example, Figure 2 appears to be screenshots and the text is distorted, which undermines clarity and professionalism.

- **Thank you for the feedback. We have remade Figure 2 using an AlphaFold analyzer tool to extract subfigures from the AlphaFold 3 structure files rather than relying on screenshots.**

2. In Figure 6, panel D appears to be mislabeled: NOX4AF3 vs NOX4QTY?

- **Thank you for bringing this to our attention. We had previously noticed this problem and addressed it. However, it appears that we forgot to make this change across both copies of the document (Manuscript with Tracked Changes and Manuscript without Tracked Changes). We have corrected this issue so both copies are correct.**

3. In Figure 6, the residue replacement direction seems reversed; it should read “Q replaces L”, etc.

- **Thank you for bringing this to our attention. We have corrected the order in the text.**

4. In Table 2 there are some extra “-” in the “Name” column.

- **Thank you for bringing this to our attention. We had previously noticed this problem and addressed it. However, it appears that we forgot to make this change across both copies of the document (Manuscript with Tracked Changes and Manuscript without Tracked Changes). We have corrected this issue so both copies are correct.**

5. For reproducibility, I suggest the authors provide the QTY-designed FASTA sequences and the corresponding native/AF3 structure files.

- **Thank you for the feedback. We have uploaded our QTY FASTA sequences as well as our AlphaFold 3 predicted structure files to a GitHub repository. It can be accessed at this link:** [**https://github.com/rickhcheng/qty_proteins**](https://github.com/rickhcheng/qty_proteins)**. We have also mentioned this in the text.**
