# Supplementary material for: Structural bioinformatic studies of eight integral transmembrane NADPH oxidases and their AlphaFold 3 predicted QTY analogs with reduced hydrophobicity
Source: PLoS One. 2026 Jun 10;21(6):e0347525. doi: 10.1371/journal.pone.0347525 (PMC13252790; doi:10.1371/journal.pone.0347525)
Supplement: Peer review history

# Peer review: Decision Letter 0

**DOI:** 10.1371/journal.pone.0347525.r001

**Authors:** Hyun Ho Park

17 Sep 2025

PONE-D-25-44849

Structural bioinformatic studies of eight integral transmembrane NADPH oxidases and their AlphaFold 3 predicted water-soluble QTY analogs

PLOS ONE

Dear Dr. Zhang

Thank you for submitting your manuscript to PLOS ONE. After careful consideration, we feel that it has merit but does not fully meet PLOS ONE’s publication criteria as it currently stands. Therefore, we invite you to submit a revised version of the manuscript that addresses the points raised during the review process.

==============================

Please provide a detailed response to the reviewer’s comment

==============================

Please submit your revised manuscript by Nov 01 2025 11:59PM. If you will need more time than this to complete your revisions, please reply to this message or contact the journal office at plosone@plos.org. When you’re ready to submit your revision, log on to https://www.editorialmanager.com/pone/ and select the ’Submissions Needing Revision’ folder to locate your manuscript file.

Please include the following items when submitting your revised manuscript:

- A rebuttal letter that responds to each point raised by the academic editor and reviewer(s). You should upload this letter as a separate file labeled ’Response to Reviewers’.
- A marked-up copy of your manuscript that highlights changes made to the original version. You should upload this as a separate file labeled ’Revised Manuscript with Track Changes’.
- An unmarked version of your revised paper without tracked changes. You should upload this as a separate file labeled ’Manuscript’.

If you would like to make changes to your financial disclosure, please include your updated statement in your cover letter. Guidelines for resubmitting your figure files are available below the reviewer comments at the end of this letter.

If applicable, we recommend that you deposit your laboratory protocols in protocols.io to enhance the reproducibility of your results. Protocols.io assigns your protocol its own identifier (DOI) so that it can be cited independently in the future. For instructions see: https://journals.plos.org/plosone/s/submission-guidelines#loc-laboratory-protocols. Additionally, PLOS ONE offers an option for publishing peer-reviewed Lab Protocol articles, which describe protocols hosted on protocols.io. Read more information on sharing protocols at https://plos.org/protocols?utm_medium=editorial-email&utm_source=authorletters&utm_campaign=protocols.

We look forward to receiving your revised manuscript.

Kind regards,

Hyun Ho Park

Academic Editor

PLOS ONE

Journal requirements:

When submitting your revision, we need you to address these additional requirements.

1. Please ensure that your manuscript meets PLOS ONE’s style requirements, including those for file naming. The PLOS ONE style templates can be found at

https://journals.plos.org/plosone/s/file?id=wjVg/PLOSOne_formatting_sample_main_body.pdf and

https://journals.plos.org/plosone/s/file?id=ba62/PLOSOne_formatting_sample_title_authors_affiliations.pdf

2. Please include captions for your Supporting Information files at the end of your manuscript, and update any in-text citations to match accordingly. Please see our Supporting Information guidelines for more information: http://journals.plos.org/plosone/s/supporting-information.

If the reviewer comments include a recommendation to cite specific previously published works, please review and evaluate these publications to determine whether they are relevant and should be cited. There is no requirement to cite these works unless the editor has indicated otherwise.

Additional Editor Comments:

Please provide a detailed response to the reviewer’s comment

[Note: HTML markup is below. Please do not edit.]

Reviewers’ comments:

Reviewer’s Responses to Questions

**Comments to the Author**

1. Is the manuscript technically sound, and do the data support the conclusions?

The manuscript must describe a technically sound piece of scientific research with data that supports the conclusions. Experiments must have been conducted rigorously, with appropriate controls, replication, and sample sizes. The conclusions must be drawn appropriately based on the data presented.

Reviewer #1: Partly

**********

2. Has the statistical analysis been performed appropriately and rigorously?

Reviewer #1: N/A

**********

3. Have the authors made all data underlying the findings in their manuscript fully available?

The PLOS Data policy requires authors to make all data underlying the findings described in their manuscript fully available without restriction, with rare exception (please refer to the Data Availability Statement in the manuscript PDF file). The data should be provided as part of the manuscript or its supporting information, or deposited to a public repository. For example, in addition to summary statistics, the data points behind means, medians and variance measures should be available. If there are restrictions on publicly sharing data—e.g. participant privacy or use of data from a third party—those must be specified.

Reviewer #1: No

**********

4. Is the manuscript presented in an intelligible fashion and written in standard English?

PLOS ONE does not copyedit accepted manuscripts, so the language in submitted articles must be clear, correct, and unambiguous. Any typographical or grammatical errors should be corrected at revision, so please note any specific errors here.

Reviewer #1: Yes

**********

5. Review Comments to the Author

Please use the space provided to explain your answers to the questions above. You may also include additional comments for the author, including concerns about dual publication, research ethics, or publication ethics. (Please upload your review as an attachment if it exceeds 20,000 characters)

Reviewer #1: The manuscript applies the QTY code method to design transmembrane proteins with reduced hydrophobicity and predicts structures with AlphaFold 3. The core claim is that extensive QTY substitutions preserve 3D folds and stability while reducing hydrophobicity, suggesting QTY analogs could serve as soluble surrogates for structural biology and downstream discovery.

Although similar approaches have been reported for other proteins, the topic remains interesting because it couples a simple design strategy with AF3-based structural assessment. However, several substantive issues must be addressed to ensure methodological rigor and to align the claims with the evidence presented.

Major issues

1. The manuscript confuses PDB codes with UniProt accessions. For example, “NOX2 (PDB: P04839)” lists a UniProt ID, not a PDB code. Please correct throughout (PDB IDs are four-character codes).

2. The text uses “DOXA1”. Should this be “DUOXA1”? And CY24A should be CYBA? Please verify and update consistently.

3. Consider moving “The rationale of the QTY code” to the Methods section for better flow.

4. The figures appear low-resolution. Please ensure they meet the journal’s figure specifications (size, dpi, font legibility).

5. Please specify the AlphaFold 3 model version/date, MSA and template settings, complex vs monomer mode, number of recycles, random seeds, and any restraints. Provide raw AF3 outputs (e.g., JSONs, PDBs, logs) to support reproducibility.

6. Without experimental validation, the manuscript cannot conclude that the designed proteins are “water-soluble.” The analyses shown indicate reduced hydrophobicity, which is not equivalent to demonstrated water solubility. To maintain rigor, replace “water-soluble QTY analogs” with “QTY analogs with reduced hydrophobicity.”

7. Beyond my point #6, please add quantitative evidence that the designs remain stable in water and show how much the hydrophobicity is reduced. To keep the work purely computational, the authors could perform a few-microsecond explicit-solvent MD simulations and/or use of protein solubility and thermostability prediction methods. This would further strengthen the claims.

Minor issues

There are several terminology errors that I noticed. The authors should proofread the manuscript more carefully.

1. pI should be “isoelectric point”, not “isoelectric focusing”.

2. RMSD is commonly used as “root-mean-square deviation”, not “residue mean square distance”, although the meaning is almost the same.

3. “kDA” should be “kDa”

4. Mixed use of “AlphaFold 3” and “AlphaFold3”.

5. Mixed use of “PyMOL” and “PyMol”.

6. You alternate between AF3/AlphaFold 3. Also, sometimes say “AlphaFold predicted” without “3”. Please consider standardize and keep labels uniform.

Also, some minor points.

1. References [4] and [31] are duplicated.

2. Use consistent decimal precision for RMSD values. I recommend two decimal points.

3. Once defined, use abbreviations consistently (e.g., pI, Q, T, Y) in subsequent text. For example, in “Notably, the protein isoelectric point (pI) remains roughly unchanged, as the substituted amino acids Q (glutamine), T(threonine), and Y (tyrosine)..”, pI, Q, T, Y have been defined above.

4. AF3 predictions for NOX5 are low confidence. Consider treating this as a cautionary case study and discuss limitations explicitly.

5. Remove subpanels labeled “No CryoEM structure available” and note the lack of experimental structures in the caption and/or main text instead.

**********

6. PLOS authors have the option to publish the peer review history of their article (what does this mean?). If published, this will include your full peer review and any attached files.

If you choose “no”, your identity will remain anonymous but your review may still be made public.

**Do you want your identity to be public for this peer review?** For information about this choice, including consent withdrawal, please see our Privacy Policy.

Reviewer #1: No

**********

[NOTE: If reviewer comments were submitted as an attachment file, they will be attached to this email and accessible via the submission site. Please log into your account, locate the manuscript record, and check for the action link "View Attachments". If this link does not appear, there are no attachment files.]

While revising your submission, please upload your figure files to the Preflight Analysis and Conversion Engine (PACE) digital diagnostic tool, https://pacev2.apexcovantage.com/. PACE helps ensure that figures meet PLOS requirements. To use PACE, you must first register as a user. Registration is free. Then, login and navigate to the UPLOAD tab, where you will find detailed instructions on how to use the tool. If you encounter any issues or have any questions when using PACE, please email PLOS at figures@plos.org. Please note that Supporting Information files do not need this step.

# Author comment: Author response to Decision Letter 1

**DOI:** 10.1371/journal.pone.0347525.r002

27 Feb 2026

Major issues

1. The manuscript confuses PDB codes with UniProt accessions. For example, “NOX2 (PDB: P04839)” lists a UniProt ID, not a PDB code. Please correct throughout (PDB IDs are four-character codes).

Thank you for the feedback. We have corrected all the PDB IDs.

2. The text uses “DOXA1”. Should this be “DUOXA1”? And CY24A should be CYBA? Please verify and update consistently.

Thank you for the feedback. We have corrected DOXA1 to DUOX1, and CY24A to CYBA. DOXA1 is an abbreviation of DUOXA1. CY24A is the antibody identifier, while CYBA is the gene/protein name.

3. Consider moving “The rationale of the QTY code” to the Methods section for better flow.

Thank you for the feedback. We have moved “The rationale of the QTY code” to the methods section.

4. The figures appear low-resolution. Please ensure they meet the journal’s figure specifications (size, dpi, font legibility).

Thank you for the feedback. We have updated figures with higher resolutions.

5. Please specify the AlphaFold 3 model version/date, MSA and template settings, complex vs monomer mode, number of recycles, random seeds, and any restraints. Provide raw AF3 outputs (e.g., JSONs, PDBs, logs) to support reproducibility.

Thank you for the feedback. We have added additional AF3 simulation information. We have specified the AlphaFold 3 parameters (monomer mode, version, MSA and template setting, seeds) in the “AlphaFold 3” section in Methods. Since we only have one protein sequence per entry, we are by default in monomer mode. There are no other user-end input changes, and all other settings are by default.

6. Without experimental validation, the manuscript cannot conclude that the designed proteins are “water-soluble.” The analyses shown indicate reduced hydrophobicity, which is not equivalent to demonstrated water solubility. To maintain rigor, replace “water-soluble QTY analogs” with “QTY analogs with reduced hydrophobicity.”

Thank you for the feedback. We have replaced “water-soluble QTY analogs” with “QTY analogs with reduced hydrophobicity” both in the title and in the main text.

7. Beyond my point #6, please add quantitative evidence that the designs remain stable in water and show how much the hydrophobicity is reduced. To keep the work purely computational, the authors could perform a few-microsecond explicit-solvent MD simulations and/or use of protein solubility and thermostability prediction methods. This would further strengthen the claims.

Thank you for the feedback. We have added additional quantitative hydrophobicity analysis using the CamSol tool (https://www-cohsoftware.ch.cam.ac.uk/index.php/camsolintrinsic). New section of “Hydrophobicity Analysis” explaining the rationale for the tool in the methods section and the new paragraph in the “Analysis of the hydrophobic surface of native NADPH oxidases and their QTY analogs with reduced hydrophobicity” section are added to address the CamSol tool. Intrinsic solubility scores are also included in Table 2 for comparison between hydrophobicity of native structures and their QTY analogs.

Minor issues

There are several terminology errors that I noticed. The authors should proofread the manuscript more carefully.

1. pI should be “isoelectric point”, not “isoelectric focusing”.

Thank you for the feedback. We have changed all “isoelectric focusing” to “isoelectric point”.

2. RMSD is commonly used as “root-mean-square deviation”, not “residue mean square distance”, although the meaning is almost the same.

Thank you for the feedback. We have updated RMSD’s full name.

3. “kDA” should be “kDa”

Thank you for the feedback. We have updated the manuscript accordingly.

4. Mixed use of “AlphaFold 3” and “AlphaFold3”.

Thank you for the feedback. We have updated all to be “AlphaFold 3”.

5. Mixed use of “PyMOL” and “PyMol”.

Thank you for the feedback. However, we were unable to find these instances.

6. You alternate between AF3/AlphaFold 3. Also, sometimes say “AlphaFold predicted” without “3”. Please consider standardize and keep labels uniform.

Thank you for the feedback. We have clarified that all mentions of “AlphaFold” refer to AlphaFold 3 and kept consistency by using AlphaFold 3 throughout.

Also, some minor points.

1. References [4] and [31] are duplicated.

Thank you for the feedback. We have deleted the duplicated reference and updated all footnote numbers.

2. Use consistent decimal precision for RMSD values. I recommend two decimal points.

Thank you for the feedback. We have changed all RMSD values to two decimal points.

3. Once defined, use abbreviations consistently (e.g., pI, Q, T, Y) in subsequent text. For example, in “Notably, the protein isoelectric point (pI) remains roughly unchanged, as the substituted amino acids Q (glutamine), T(threonine), and Y (tyrosine)..”, pI, Q, T, Y have been defined above.

Thank you for the feedback. We have deleted the abbreviation descriptions.

4. AF3 predictions for NOX5 are low confidence. Consider treating this as a cautionary case study and discuss limitations explicitly.

Thank you for the feedback. In the section titled “Superpositions of CryoEM structures with AlphaFold 3–predicted native structures and their QTY analogs with reduced hydrophobicity,” we have added a discussion explaining how the low confidence of NOX5’s AlphaFold 3 prediction likely contributes to the high RMSD observed between its native structure and the AlphaFold-predicted QTY analog. We present this as an important caution: although AlphaFold 3 can serve as a useful reference tool for evaluating the efficacy of the QTY code, its predictions are not perfect. Therefore, the elevated RMSD score may not necessarily indicate a failure of the QTY code but may instead reflect limitations in the AlphaFold 3 prediction itself.

5. Remove subpanels labeled “No CryoEM structure available” and note the lack of experimental structures in the caption and/or main text instead.

Thank you for the feedback. We have removed these panels and noted absence of CryoEM structures in captions of Figures 3 and 5.

Attachment Submitted filename: Response to Reviewers.docx

# Peer review: Decision Letter 1

**DOI:** 10.1371/journal.pone.0347525.r003

**Authors:** Hyun Ho Park

5 Mar 2026

PONE-D-25-44849R1

Structural bioinformatic studies of eight integral transmembrane NADPH oxidases and their AlphaFold 3 predicted QTY analogs with reduced hydrophobicity

PLOS One

Dear Dr. Zhang,

Thank you for submitting your manuscript to PLOS ONE. After careful consideration, we feel that it has merit but does not fully meet PLOS ONE’s publication criteria as it currently stands. Therefore, we invite you to submit a revised version of the manuscript that addresses the points raised during the review process.

Please submit your revised manuscript by Apr 19 2026 11:59PM. If you will need more time than this to complete your revisions, please reply to this message or contact the journal office at plosone@plos.org. When you’re ready to submit your revision, log on to https://www.editorialmanager.com/pone/ and select the ’Submissions Needing Revision’ folder to locate your manuscript file.

Please include the following items when submitting your revised manuscript:

- A letter that responds to each point raised by the academic editor and reviewer(s). You should upload this letter as a separate file labeled ’Response to Reviewers’.
- A marked-up copy of your manuscript that highlights changes made to the original version. You should upload this as a separate file labeled ’Revised Manuscript with Track Changes’.
- An unmarked version of your revised paper without tracked changes. You should upload this as a separate file labeled ’Manuscript’.

If you would like to make changes to your financial disclosure, please include your updated statement in your cover letter. Guidelines for resubmitting your figure files are available below the reviewer comments at the end of this letter.

If applicable, we recommend that you deposit your laboratory protocols in protocols.io to enhance the reproducibility of your results. Protocols.io assigns your protocol its own identifier (DOI) so that it can be cited independently in the future. For instructions see: https://journals.plos.org/plosone/s/submission-guidelines#loc-laboratory-protocols. Additionally, PLOS ONE offers an option for publishing peer-reviewed Lab Protocol articles, which describe protocols hosted on protocols.io. Read more information on sharing protocols at https://plos.org/protocols?utm_medium=editorial-email&utm_source=authorletters&utm_campaign=protocols.

We look forward to receiving your revised manuscript.

Kind regards,

Hyun Ho Park

Academic Editor

PLOS One

Journal Requirements:

If the reviewer comments include a recommendation to cite specific previously published works, please review and evaluate these publications to determine whether they are relevant and should be cited. There is no requirement to cite these works unless the editor has indicated otherwise.

**Additional Editor Comments:**

The reviewer still has some concerns regarding the manuscript.

Please prepare and submit a response addressing these concerns by April 4.

Reviewers’ comments:

Reviewer’s Responses to Questions

**Comments to the Author**

1. If the authors have adequately addressed your comments raised in a previous round of review and you feel that this manuscript is now acceptable for publication, you may indicate that here to bypass the “Comments to the Author” section, enter your conflict of interest statement in the “Confidential to Editor” section, and submit your "Accept" recommendation.

Reviewer #1: (No Response)

**********

2. Is the manuscript technically sound, and do the data support the conclusions?

The manuscript must describe a technically sound piece of scientific research with data that supports the conclusions. Experiments must have been conducted rigorously, with appropriate controls, replication, and sample sizes. The conclusions must be drawn appropriately based on the data presented.

Reviewer #1: Partly

**********

3. Has the statistical analysis been performed appropriately and rigorously?

Reviewer #1: No

**********

4. Have the authors made all data underlying the findings in their manuscript fully available?

The PLOS Data policy requires authors to make all data underlying the findings described in their manuscript fully available without restriction, with rare exception (please refer to the Data Availability Statement in the manuscript PDF file). The data should be provided as part of the manuscript or its supporting information, or deposited to a public repository. For example, in addition to summary statistics, the data points behind means, medians and variance measures should be available. If there are restrictions on publicly sharing data—e.g. participant privacy or use of data from a third party—those must be specified.

Reviewer #1: No

**********

5. Is the manuscript presented in an intelligible fashion and written in standard English?

PLOS ONE does not copyedit accepted manuscripts, so the language in submitted articles must be clear, correct, and unambiguous. Any typographical or grammatical errors should be corrected at revision, so please note any specific errors here.

Reviewer #1: Yes

**********

6. Review Comments to the Author

Please use the space provided to explain your answers to the questions above. You may also include additional comments for the author, including concerns about dual publication, research ethics, or publication ethics. (Please upload your review as an attachment if it exceeds 20,000 characters)

Reviewer #1: I still have major concerns that leave me unconvinced by the current results, and I continue to recommend major revision.

Major issues

1. After revision, the term “water-soluble” still appears in figure captions and the main text (e.g., “confirming exceptional structural conservation between native NADPH oxidases and their water-soluble QTY variants”). I am not convinced that the QTY designs are water-soluble without experimental evidence. Water-soluble should be framed explicitly as a hypothesis and the manuscript should be revised accordingly throughout.

2. In the conclusion, the authors claim that “our findings reveal remarkable resemblance between QTY analogs and their native proteins, suggesting minimal functional impact despite significant amino acid substitution.” I am not convinced because the QTY structures are all AlphaFold 3 predictions, not experimental structures. On the other hand, even if the global fold remains similar, function could still be compromised if substitutions perturb key interactions or interfaces with binding partners etc. These statements should be substantially softened, and the limitations of using AF3 structure as a validation should be made more explicit.

3. Similarly, I think the statements like “may function as water-soluble reducing agents for controlling ROS levels…” is a far reach.

4. Panel labels for Figures 3 and 5 should be updated, and figure citations in the captions and main text should be corrected for consistency.

5. In Table 2, where cryo-EM structures are available, RMSDs for QTY analogs should be calculated against the experimental cryo-EM structures, not against AF3 structures. Only when experimental structures are unavailable should AF3 predictions be used, and those cases should be clearly marked as such.

6. Related to point 5, I suggest the authors merge Figures 4 and 5 so that QTY structures are compared directly with cryo-EM structures when available. When cryo-EM structures are unavailable, comparison to AF3 predictions is acceptable, but must be clearly marked. Comparing QTY structures to AF3 structures in cases where cryo-EM structures exist is not informative.

Minor issues

1. I do not recommend using screenshots as figures. The same information can be presented using the AlphaFold server output data and generate figures by the authors. For example, Figure 2 appears to be screenshots and the text is distorted, which undermines clarity and professionalism.

2. In Figure 6, panel D appears to be mislabeled: NOX4AF3 vs NOX4QTY?

3. In Figure 6, the residue replacement direction seems reversed; it should read “Q replaces L”, etc.

4. In Table 2 there are some extra “-” in the “Name” column.

5. For reproducibility, I suggest the authors provide the QTY-designed FASTA sequences and the corresponding native/AF3 structure files.

**********

7. PLOS authors have the option to publish the peer review history of their article (what does this mean?). If published, this will include your full peer review and any attached files.

If you choose “no”, your identity will remain anonymous but your review may still be made public.

**Do you want your identity to be public for this peer review?** For information about this choice, including consent withdrawal, please see our Privacy Policy.

Reviewer #1: No

**********

[NOTE: If reviewer comments were submitted as an attachment file, they will be attached to this email and accessible via the submission site. Please log into your account, locate the manuscript record, and check for the action link "View Attachments". If this link does not appear, there are no attachment files.]

To ensure your figures meet our technical requirements, please review our figure guidelines: https://journals.plos.org/plosone/s/figures

You may also use PLOS’s free figure tool, NAAS, to help you prepare publication quality figures: https://journals.plos.org/plosone/s/figures#loc-tools-for-figure-preparation.

NAAS will assess whether your figures meet our technical requirements by comparing each figure against our figure specifications.

# Author comment: Author response to Decision Letter 2

**DOI:** 10.1371/journal.pone.0347525.r004

1 Apr 2026

Major issues

1. After revision, the term “water-soluble” still appears in figure captions and the main text (e.g., “confirming exceptional structural conservation between native NADPH oxidases and their water-soluble QTY variants”). I am not convinced that the QTY designs are water-soluble without experimental evidence. Water-soluble should be framed explicitly as a hypothesis and the manuscript should be revised accordingly throughout.

• Thank you for the feedback. We had previously noticed this problem and addressed it. However, it appears that we forgot to make this change across both copies of the document (Manuscript with Tracked Changes and Manuscript without Tracked Changes). We have corrected this issue so both copies are correct.

• Additionally, we have also softened the language throughout the text and cautioned the limitations of computation prediction methods without experimental validation.

2. In the conclusion, the authors claim that “our findings reveal remarkable resemblance between QTY analogs and their native proteins, suggesting minimal functional impact despite significant amino acid substitution.” I am not convinced because the QTY structures are all AlphaFold 3 predictions, not experimental structures. On the other hand, even if the global fold remains similar, function could still be compromised if substitutions perturb key interactions or interfaces with binding partners etc. These statements should be substantially softened, and the limitations of using AF3 structure as a validation should be made more explicit.

• Thank you for the feedback. We have substantially softened the language in the section and cautioned the limitations of computational prediction methods without experimental validation.

3. Similarly, I think the statements like “may function as water-soluble reducing agents for controlling ROS levels…” is a far reach.

• Thank you for the feedback. We have removed this statement from the text.

4. Panel labels for Figures 3 and 5 should be updated, and figure citations in the captions and main text should be corrected for consistency.

• Thank you for your feedback. We’ve updated the captions for Figure 3 and 5 accordingly.

5. In Table 2, where cryo-EM structures are available, RMSDs for QTY analogs should be calculated against the experimental cryo-EM structures, not against AF3 structures. Only when experimental structures are unavailable should AF3 predictions be used, and those cases should be clearly marked as such.

• Thank you for your feedback. It appears we had not labeled the table correctly. Our RMSD values had previously been calculated against CryoEM structures when available, and AlphaFold 3 predicted native structures when unavailable. We have clarified this in the table caption.

• Upon closer inspection, we also noticed some minor errors in the QTY sequences for some of the proteins. We have redone all relevant calculations to account to ensure accuracy.

6. Related to point 5, I suggest the authors merge Figures 4 and 5 so that QTY structures are compared directly with cryo-EM structures when available. When cryo-EM structures are unavailable, comparison to AF3 predictions is acceptable, but must be clearly marked. Comparing QTY structures to AF3 structures in cases where cryo-EM structures exist is not informative.

• Thank you for the feedback. We combined Figures 3 and 4 so that QTY structures were compared directly with CryoEM structures when available and compared to AlphaFold 3 predicted native structures when not available. We have also updated the figure caption accordingly.

• We have kept Figure 5 as it may serve as a useful comparison for the 3 different superposed structures, when available (AF3 predicted QTY, AF3 predicted native, CryoEM native.

Minor issues

1. I do not recommend using screenshots as figures. The same information can be presented using the AlphaFold server output data and generate figures by the authors. For example, Figure 2 appears to be screenshots and the text is distorted, which undermines clarity and professionalism.

• Thank you for the feedback. We have remade Figure 2 using an AlphaFold analyzer tool to extract subfigures from the AlphaFold 3 structure files rather than relying on screenshots.

2. In Figure 6, panel D appears to be mislabeled: NOX4AF3 vs NOX4QTY?

• Thank you for bringing this to our attention. We had previously noticed this problem and addressed it. However, it appears that we forgot to make this change across both copies of the document (Manuscript with Tracked Changes and Manuscript without Tracked Changes). We have corrected this issue so both copies are correct.

3. In Figure 6, the residue replacement direction seems reversed; it should read “Q replaces L”, etc.

• Thank you for bringing this to our attention. We have corrected the order in the text.

4. In Table 2 there are some extra “-” in the “Name” column.

• Thank you for bringing this to our attention. We had previously noticed this problem and addressed it. However, it appears that we forgot to make this change across both copies of the document (Manuscript with Tracked Changes and Manuscript without Tracked Changes). We have corrected this issue so both copies are correct.

5. For reproducibility, I suggest the authors provide the QTY-designed FASTA sequences and the corresponding native/AF3 structure files.

• Thank you for the feedback. We have uploaded our QTY FASTA sequences as well as our AlphaFold 3 predicted structure files to a GitHub repository. It can be accessed at this link: https://github.com/rickhcheng/qty_proteins. We have also mentioned this in the text.

Attachment Submitted filename: 3.26 - Revision.2 - Response to Reviewers.docx

# Peer review: Decision Letter 2

**DOI:** 10.1371/journal.pone.0347525.r005

**Authors:** Hyun Ho Park

5 Apr 2026

Structural bioinformatic studies of eight integral transmembrane NADPH oxidases and their AlphaFold 3 predicted QTY analogs with reduced hydrophobicity

PONE-D-25-44849R2

Dear Dr. ZHANG,

We’re pleased to inform you that your manuscript has been judged scientifically suitable for publication and will be formally accepted for publication once it meets all outstanding technical requirements.

Within one week, you’ll receive an e-mail detailing the required amendments. When these have been addressed, you’ll receive a formal acceptance letter and your manuscript will be scheduled for publication.

An invoice will be generated when your article is formally accepted. Please note, if your institution has a publishing partnership with PLOS and your article meets the relevant criteria, all or part of your publication costs will be covered. Please make sure your user information is up-to-date by logging into Editorial Manager at Editorial Manager® and clicking the ‘Update My Information’ link at the top of the page. For questions related to billing, please contact billing support.

If your institution or institutions have a press office, please notify them about your upcoming paper to help maximize its impact. If they’ll be preparing press materials, please inform our press team as soon as possible -- no later than 48 hours after receiving the formal acceptance. Your manuscript will remain under strict press embargo until 2 pm Eastern Time on the date of publication. For more information, please contact onepress@plos.org.

Kind regards,

Hyun Ho Park

Academic Editor

PLOS One

Reviewers’ comments:

Reviewer’s Responses to Questions

**Comments to the Author**

1. If the authors have adequately addressed your comments raised in a previous round of review and you feel that this manuscript is now acceptable for publication, you may indicate that here to bypass the “Comments to the Author” section, enter your conflict of interest statement in the “Confidential to Editor” section, and submit your "Accept" recommendation.

Reviewer #1: All comments have been addressed

**********

2. Is the manuscript technically sound, and do the data support the conclusions?

The manuscript must describe a technically sound piece of scientific research with data that supports the conclusions. Experiments must have been conducted rigorously, with appropriate controls, replication, and sample sizes. The conclusions must be drawn appropriately based on the data presented.

Reviewer #1: Yes

**********

3. Has the statistical analysis been performed appropriately and rigorously?

Reviewer #1: (No Response)

**********

4. Have the authors made all data underlying the findings in their manuscript fully available?

The PLOS Data policy requires authors to make all data underlying the findings described in their manuscript fully available without restriction, with rare exception (please refer to the Data Availability Statement in the manuscript PDF file). The data should be provided as part of the manuscript or its supporting information, or deposited to a public repository. For example, in addition to summary statistics, the data points behind means, medians and variance measures should be available. If there are restrictions on publicly sharing data—e.g. participant privacy or use of data from a third party—those must be specified.

Reviewer #1: Yes

**********

5. Is the manuscript presented in an intelligible fashion and written in standard English?

PLOS ONE does not copyedit accepted manuscripts, so the language in submitted articles must be clear, correct, and unambiguous. Any typographical or grammatical errors should be corrected at revision, so please note any specific errors here.

Reviewer #1: Yes

**********

6. Review Comments to the Author

Please use the space provided to explain your answers to the questions above. You may also include additional comments for the author, including concerns about dual publication, research ethics, or publication ethics. (Please upload your review as an attachment if it exceeds 20,000 characters)

Reviewer #1: (No Response)

**********

7. PLOS authors have the option to publish the peer review history of their article (what does this mean?). If published, this will include your full peer review and any attached files.

If you choose “no”, your identity will remain anonymous but your review may still be made public.

**Do you want your identity to be public for this peer review?** For information about this choice, including consent withdrawal, please see our Privacy Policy.

Reviewer #1: No

**********

# Editor report: Acceptance letter

**DOI:** 10.1371/journal.pone.0347525.r006

**Authors:** Hyun Ho Park

PONE-D-25-44849R2

PLOS One

Dear Dr. Zhang,

I’m pleased to inform you that your manuscript has been deemed suitable for publication in PLOS One. Congratulations! Your manuscript is now being handed over to our production team.

At this stage, our production department will prepare your paper for publication. This includes ensuring the following:

* All references, tables, and figures are properly cited

* All relevant supporting information is included in the manuscript submission,

* There are no issues that prevent the paper from being properly typeset

You will receive further instructions from the production team, including instructions on how to review your proof when it is ready. Please keep in mind that we are working through a large volume of accepted articles, so please give us a few days to review your paper and let you know the next and final steps.

Lastly, if your institution or institutions have a press office, please let them know about your upcoming paper now to help maximize its impact. If they’ll be preparing press materials, please inform our press team within the next 48 hours. Your manuscript will remain under strict press embargo until 2 pm Eastern Time on the date of publication. For more information, please contact onepress@plos.org.

You will receive an invoice from PLOS for your publication fee after your manuscript has reached the completed accept phase. If you receive an email requesting payment before acceptance or for any other service, this may be a phishing scheme. Learn how to identify phishing emails and protect your accounts at https://explore.plos.org/phishing.

If we can help with anything else, please email us at customercare@plos.org.

Thank you for submitting your work to PLOS ONE and supporting open access.

Kind regards,

PLOS ONE Editorial Office Staff

on behalf of

Dr. Hyun Ho Park

Academic Editor

PLOS One
